# Reduced Schiff Base Copper(II) Complexes Derived from 2-, 3-, and 4-Methoxybenzylamine: DNA Binding and Cleavage, BSA Binding, Cytotoxic and SOD Mimetic Properties

**DOI:** 10.3390/ijms27198650

**Published:** 2026-09-27

**Authors:** Lucia Lintnerová, Peter Herich, Jana Korec, Barbora Svitková, Flóra Jozefíková, Karol Lušpai, Jindra Valentová

**Affiliations:** 1 Department of Chemical Theory of Drugs, Faculty of Pharmacy, Comenius University in Bratislava, Odbojárov 10, 832 32 Bratislava, Slovakia; peter.herich@stuba.sk (P.H.); flora.jozefikova@stuba.sk (F.J.); 2 Department of Physical Chemistry, Faculty of Chemical and Food Technology, Slovak University of Technology, Radlinského 9, 812 37 Bratislava, Slovakia; karol.luspai@stuba.sk; 3 Cancer Research Institute, Biomedical Research Center, Slovak Academy of Sciences, Dúbravská cesta 9, 845 05 Bratislava, Slovakia; barbora.svitkova@savba.sk; 4 Department of Inorganic Chemistry, Faculty of Chemical and Food Technology, Slovak University of Technology, Radlinského 9, 812 37 Bratislava, Slovakia

**Keywords:** reduced Shiff base, copper complexes, DNA binding, BSA binding, DNA cleavage, antimitochondrial activity, radical scavenging

## Abstract

A series of reduced Schiff base ligands derived from salicylaldehyde (**1**) and methoxybenzylamines (**2a–c**) were prepared and used to prepare three copper(II) complexes, **5a** (2-methoxyderivative), **5b** (3-methoxyderivative), and **5c** (4-methoxyderivative). They were characterized by spectral methods and X-ray crystallography. The complexes showed DNA cleavage activity, with 30% cleavage at 3 × 10^−3^ M and up to 94.5% at 5 × 10^−3^ M. Absorption titration studies with ct-DNA and EB-DNA displacement methods revealed strong binding of the prepared complexes, with complexes **5b** and **5c** favored. Viscosity measurements and thermal degradation of DNA studies indicate DNA groove binding. All complexes exhibit a moderately strong binding affinity to serum albumin. The complexes performed very well in the resazurin assay monitoring mitochondrial activity, with significant cytotoxic effects (around 95% already at 1 × 10^−4^ M). The MTT assay confirmed the complexes’ cytotoxicity against cancer cell lines (A549, HepG2) compared with HEL fibroblasts. All complexes showed remarkable selectivity for HepG2 cells, with selectivity indices (HEL vs. HepG2) up to 3.05 for complex **5a**, and complex **5b** achieved the lowest IC_50_ (33.0 ± 5.3 μM) against HepG2 cells. From a regioisomerism perspective, complex **5a** outperformed the others in DNA cleavage; complex **5b,** with the 3-methoxy group, showed the most interesting cytotoxicity results, and both complexes **5b** and **5c** showed good DNA-binding properties.

## 1. Introduction

The coordination compounds of various Schiff base ligands represent a highly studied group of compounds. The condensation reaction of various amines and carbonyl compounds allows for the synthesis of custom-made structures. At the same time, the Schiff base imine group introduces a strong metal-binding center. This enables the generation of highly active compounds. Schiff base ligands where the C=N bond is conjugated to aromatic rings have become desirable in drug design for their planar features [1,2,3].

Reduced Schiff bases are one derivatization pathway for Schiff bases. They are a class of secondary amines with greater chemical stability than Schiff bases. However, because the C=N bond is reduced, the structure loses some of its planar character. After reduction, the structure becomes more flexible, allowing changes in the geometry of the central atom’s coordination sphere and greater adaptability to targets. This can, for example, improve binding of the metal center to DNA and its cleavage [4,5,6]. Easier access to the metal center could also benefit redox reactions on biomacromolecules. This could enable stronger interaction with DNA and DNA cleavage, ROS generation in the cell or even in mitochondria, and lead to cytotoxicity and apoptotic cell death [7,8]. Alternatively, SOD mimetic properties and radical scavenging require a change in the oxidation number of the metal atom. This can happen more effectively and at a higher rate if some geometry changes are possible [9,10,11].

The reduced Schiff bases also combine an amine and an aldehyde or ketone within the structure. Frequently used amine fragments in the reduced Schiff base ligand are derived from amino acids [6,10,12,13,14,15] or an amine containing a metal-binding pyridine or an aromatic part with N or O functional group [8,9,16,17,18,19]. The aldehyde part often contains an ortho-substituted aromatic fragment derived, for example, from salicylaldehyde or analogous naphthalene or pyridine carbaldehyde derivatives [4,17,20]. The ortho position of a hydroxyl or amine group is highly suitable for chelate binding to copper or other metals [5,15,19,21,22,23]. Reduced Schiff bases have also been modified on the amine nitrogen to introduce another, usually *N*-carrying, fragment for metal binding and chelate ring formation [24,25,26,27]. These structural variations allow tuning of the coordination properties of the ligands with possible di-, tri-, or tetradentate coordination, sometimes also combined with an *N*-heteroaromatic secondary ligand [10,11,12,14].

Structural tuning also enables the preparation of complexes with diverse chemical, physical, and biological activities. Complexes of reduced Schiff bases of copper and other metals have been reported for antibacterial, antifungal, antiradical, anticancer, cytotoxic, antioxidant, antidiabetic, and enzyme-mimicking properties [7,8,9,11,23,28,29,30]. Their anticancer and cytotoxic activities were associated with DNA-binding and DNA-cleaving properties [2,4,6,18,19,31,32], as well as cell-cycle arrest, apoptosis induction, and inhibition of cell proliferation [8,9,10,14,17,27] and proteasome inhibition [16,33].

The ability of metal complexes to interact with DNA has been known since the discovery of the first platinum-based chemotherapeutics, such as cisplatin. But complexes based on other transition metals, mainly copper, reveal a less toxic, more biocompatible alternative. Additionally, the broader list of activities of copper complexes—mainly due to the role of copper in metalloenzymes and other biological systems—improves the potential of copper-based drugs. Copper alters metabolism in cancer cells; recent studies have suggested several pathways, such as inhibition of proteasome activity, modulation of ROS levels, apoptosis induction targeting caspases, DNA damage, or angiogenesis [8,13,31,33,34,35,36]. Although the mechanism of action is not fully clear, cancer cells have higher copper content than normal cells. As a result, copper-based drugs may be more selective for cancer cells [1,19,24].

Reduced Schiff base copper complexes can bind DNA via noncovalent interactions such as intercalation, groove binding, or electrostatic interaction with the phosphate-sugar DNA backbone [11,18,19,32]. Covalent coordination bonding similar to platinum-based complexes is also possible [37]. In addition to binding, the complexes can cleave DNA, introducing another class of DNA metallonucleases capable of hydrolytic or oxidative cleavage [6,18]. ROS generators such as hydrogen peroxide or oxygen [26,32,36] or a reducing agent such as ascorbic acid [24] can assist DNA cleavage. Reduced Schiff base copper(II) complexes, which can interact with DNA by binding and/or cleaving, were shown to be antitumor agents in vitro and in vivo testing [16,17].

The copper center is often associated with oxidation–reduction reactions because it readily changes its oxidation number between II and I under aqueous, biocompatible conditions. Copper complexes are often referred to as SOD mimetics; in other words, radical scavengers able to give and/or take one electron [30,38,39]. They mimic superoxide dismutase (SOD) enzymes by converting superoxide (O_2_^−•^) to oxygen (O_2_) by oxidation and to peroxide anion (O_2_^2−^) by reduction [31,40,41]. Reactive oxygen species (ROS) such as superoxide, hydroxyl radical, or hydrogen peroxide form naturally in the organism, mainly in mitochondria. They also play a role in immunity and defense processes against pathogens. ROS overproduction due to external sources (infections, increased uric acid production, auto-oxidation of thiols, hyperglycemia, etc.) can lead to pathological states and tissue damage [7,30,42,43]. This can also cause cytotoxicity in cancer cells. SOD mimetics, thanks to their both antioxidant and prooxidant properties, could also serve as ROS generators [9,18] and act, for example, as DNA cleavers in the presence of ROS [4,6].

In our previous research, we prepared a series of copper(II) complexes from Schiff bases of salicylaldehyde (**1**) and 2-, 3-, and 4-methoxybenzylamines (**2a–c**). The research monitored structural properties and biological activities similar to those in this study [44]. However, in this study we focused on the reduced Schiff ligands **4a–c** and their copper(II) complexes (**5a–c**). Reduced Schiff bases, as mentioned above, represent an interesting structural variation that brings different properties and features compared to Schiff bases and is worth studying. The various activities of the complexes were studied from the point of view of regioisomerism of the methoxy group (**a**: 2-methoxy, **b**: 3-methoxy, **c**: 4-methoxyderivative) on the benzylamine part of the ligand molecule. For this purpose, DNA binding and DNA cleavage assays, cell viability, and tumor cell cytotoxicity MTT (3-[4,5-dimethylthiazol-2-yl]-2,5 diphenyl tetrazolium) assays were performed. Bovine serum albumin (BSA) binding properties of the prepared complexes were measured to determine potential drug delivery to solid tumors. Alternatively, their SOD mimetic activities were studied using the INT (2-(4-iodophenyl)-3-(4-nitrophenyl)-5-phenyl-2H-tetrazolium) radical scavenging method.

## 2. Results and Discussion

### 2.1. Synthesis

The synthesis of Schiff bases **3a–c** started from salicylaldehyde (**1**) and 2-, 3-, and 4-methoxybenzylamine (**2a–c**). The reaction was refluxed in dry methanol for 1–2 h (Scheme 1). Reaction progress was monitored by color change to yellow and by thin layer chromatography (TLC). Schiff bases were not isolated in solid form but were directly reduced using NaBH_4_ to obtain the reduced Schiff bases **4a–c** after stirring at room temperature for 30–60 min. The procedure was performed analogously for all three compounds. The reduction of the double bond was observed by a color change to an almost colorless product. The solvent was removed by evaporation to dryness; the organic product was isolated by extraction (solid/liquid) into chloroform and further purified by column flash chromatography on silica gel. Compounds **4a** and **4b** were clear beige oils, and **4c** solidified into a white solid.

The complexes of reduced Schiff bases **4a–c** were prepared in methanol using copper(II) acetate. Complexation occurred readily, and we isolated green powders of **5a–c** in good yield with satisfactory elemental analysis. The remaining reaction mixtures were left to evaporate at room temperature. To promote further crystallization, a few drops of distilled water were added to the mixtures, and after 1–3 days, all three complexes produced dark green crystals used for X-ray analysis.

Besides structure analysis and EPR spectra, the complexes **5a–c** were characterized by spectral methods (UV-Vis, IR, elemental analysis), and the free ligands **4a–c** were also characterized by ^1^H and ^13^C NMR. NMR spectroscopy revealed the reduction of the Schiff bases. In the ^1^H spectrum of the Schiff base **3c,** the hydrogen in the Schiff base group (-CH=N-) has the chemical shift 8.382 ppm (Figure S1-1). After the reduction, a secondary amine group with two hydrogens (-CH_2_-N-) can be seen with chemical shifts in the range 3.99–3.75 ppm (**4c** spectra in Figure S1-2). The ^13^C NMR also confirmed the presence of an sp^3^-hybridized carbon bound to the secondary amine with shifts around 52 ppm. IR spectroscopy was used to characterize the ligands **4a–c** and the copper(II) complexes **5a–c**. The spectra of the reduced Schiff bases **4a–c** are mutually very similar. The main differences between the ligand and complex spectra are the shift of the N-H peak from 3240 to 3340 cm^−1^ to lower values around 3200 cm^−1^. Also, the carboxylic groups from the acetates introduced during complexation appear around 1560 and 1400 cm^−1^. The C-O bond signals are similar for both complexes and ligands, but a shift in the frequency of the phenolic C-O bond can be seen—from around 1250 cm^−1^ in the ligands to around 1340 cm^−1^ in the complexes. Figures S1-12, S1-13, and S1-14 show the IR spectra. The UV-Vis spectra of the complexes **5a–c** show four absorption peaks. The complexes show a low-intensity d-d transition band around 710–720 nm, a charge transfer band around 400 nm, and two high-absorbance peaks in the 250–290 nm range corresponding to intraligand n-π* and π-π* transitions. Elemental analyses of the ligands and complexes are in agreement with calculated values and, in the case of the complexes, also with the X-ray analysis data.

### 2.2. X-Ray Structure of Prepared Complexes

All three complexes crystallized from methanol/water mixtures as dark green crystals suitable for X-ray analysis. Complexes **5b** and **5c** crystallized as very similar binuclear complexes with 2:2:2 ligand:copper:acetate ratios. In comparison, **5b** also contained one asymmetric water molecule per four binuclear units bound to one copper center. Complex **5a** is a polymer of binuclear units and also has the 2:2:2 ratios; the acetate ligands serve as bridges between neighboring copper centers.

Complex **5a** crystallized in the monoclinic *P*n acentric space group (No. 7) as a polymer of binuclear units. The molecular structure for complex **5a** is shown in Figure 1 (polymer chain detail), and its crystallographic data are summarized in Table 1. The unit cell contains two independent molecular polymers (Figure S2-1). Complex **5a** consists of two reduced Schiff base ligands and two acetate anion ligands bonded over central atoms. Complex **5a** is formed from binuclear molecules linked via a bridging acetate bound to the Cu2 center. The copper center Cu1 has a disordered square-planar arrangement (geometrical index τ_4_ = 0.17/τ′_4_ = 0.16), and the Cu2 central atom arrangement is a weakly disordered square-pyramid (geometrical index τ_5_ = 0.15). Selected bond lengths (with marked equatorial and axial positions) and angles are in Table 2.

A system of intermolecular and intramolecular hydrogen bonds and interactions stabilizes the crystal structure of complex **5a** (Figure S2-1). Figure S2-2 and Table S2-1 report the parameters of these hydrogen bonds and interactions.

Complex **5b** crystallized in the monoclinic *P*2_1_/n space group (No. 14) as one independent molecule (Figure 2) with four molecules in the unit cell (Figure S2-3). Complex **5b** consists of two reduced Schiff base ligands, acetate anions, and a disordered water molecule. The polyhedron around the central atom (Cu1) is a disordered square pyramid (with geometrical index τ_5_ = 0.14), and the central atom (Cu2) is disordered square-planar (with geometrical index τ_4_ = 0.25/τ′_4_ = 0.23); selected bond lengths (with marked equatorial and axial positions) and angles are given in Table 3. The water molecule is in a special position with quarter occupancy. Table 1 presents the crystallographic data.

A system of intermolecular hydrogen bonds and interactions stabilizes the crystal structure of complex **5b** (Figure S2-3). Figure S2-4 and Table S2-2 report the hydrogen-bond and interaction parameters.

The complex **5c** crystallized in the triclinic *P*-1 centrosymmetric space group (No. 2) as one independent molecule (Figure 3—symmetric binuclear complex) with one molecule in the unit cell (Figure S2-5). Table 1 summarizes the crystallographic data. The complex **5c** consists of two reduced Schiff base ligands and two acetate anions bonded to the Cu1 and Cu1* central atoms. The polyhedron around the central atom Cu1 is a weakly disordered square-planar (geometrical index τ_4_ = 0.20/τ′_4_ = 0.18) with selected bond lengths (with marked equatorial and axial positions) and angles summarized in Table 4.

A system of intermolecular hydrogen bonds and interactions stabilizes the crystal structure of complex **5c** (Figure S2-5). Figure S2-6 and Table S2-3 report the hydrogen-bond and interaction parameters.

### 2.3. EPR Spectral Characteristics

An X-band EPR spectrum of complex **5a** observed at 300 K exhibits signals at 500 G, 3200 G, and 4200 G. The signal at 4200 G can be attributed to the parallel component of the triplet Δm_s_ = ±1 transition, g‖, in the triplet state of the molecule containing two paramagnetic copper centers in interaction. For a triplet (S = 1) state, the formally forbidden Δm_s_ = ±2 transition appears at roughly half the field of the “full-field” Δm_s_ = ±1 transition. In the absence of ZFS, this would fall near ~1600 G for X-band. The signal at 500 G is, however, probably a half-field signal of the triplet state from the dimer, and the large downfield shift to ~500 G indicates a moderate zero-field splitting (dipolar coupling D with a small contribution of E), which mixes the m_s_ sublevels and redistributes transition energies. The signal at 3200 G is a mixture of the monomeric copper defect (the sharp signal) and the overlapping perpendicular component of the triplet Δm_s_ = ±1 transition, g⊥. The structural asymmetry (square planar vs. square pyramidal) breaks local symmetry and enhances the anisotropic exchange, because the two Cu sites have different g-tensors and different spin–orbit coupling matrices. This inequivalence feeds directly into a larger effective D ≈ 0.26 cm^−1^ and a non-negligible small rhombic parameter E ≈ 0.01 cm^−1^.

With an increase in temperature from 300 to 340 K (Figure S1-15), no change in the spectra was observed. By cooling to 100 K (Figure 4), the monomeric signal sharpened and turned into a standard axial copper signal with resolved 4-line Cu splitting. The dimeric signal significantly decreased in intensity, which indicates the depopulation of the triplet S = 1 state. This is characteristic of antiferromagnetic coupling (J < 0) and a singlet ground state (the triplet state is thermally excited). At low temperature, the parallel component of the S = 1 signal shifts to higher field (lower g-value), which is a direct spectroscopic signature of an increasing zero-field splitting upon cooling, driven probably by lattice contraction that shortens the Cu···Cu distance and tightens the μ-O bridge geometry. This is entirely consistent with the antiferromagnetically coupled, structurally asymmetric binuclear Cu(II) model and adds valuable quantitative information about the rigidity and thermal response of the exchange pathway.

According to the crystal structure, complex **5b** consists exclusively of binuclear molecules (only two copper atoms per molecule). EPR spectra measured at 300 K exhibit a half-field S = 1 triplet signal shifted close to zero (100 G), indicating an exceptionally large zero-field splitting (high ∣D∣ value), stronger than in complex **5a**. The ZFS mixes the spin states so strongly that the resonance condition is drastically altered. We also observed a perpendicular triplet component at 3200 G, a parallel triplet component at 4600 G, and a very small signal at 5600 G, probably the high-field parallel triplet component (g∥+). By increasing the temperature to 360 K (Figure S1-16, left), the triplet half-field signal as well as the triplet parallel signal decreases, but the triplet perpendicular signal remains unchanged. The sample probably contains a significant proportion of monomeric impurity insensitive to temperature change. At low temperature (100K) (Figure 4), the half-field signal exhibits hyperfine structure, and all signals lose their intensity and sharpen. Only the small signal at 5600 G disappeared almost completely, probably becoming forbidden at low temperature. Because the Δm_s_ = ±2 transition has a compressed field scale, the nuclear hyperfine interactions (I = 3/2 for ^65^Cu/^65^Cu) can be exceptionally well-resolved when lines sharpen at low temperature. Other confirmation is measurement in parallel mode, where forbidden transitions are more intense and allowed transitions are damped, compared to standard perpendicular EPR mode (Figure S1-16, right). For a binuclear complex with two equivalent copper nuclei, we observed a characteristic 7-line hyperfine pattern. The decrease in the intensity at low temperatures for triplet signals suggests an antiferromagnetically coupled dimer (J < 0). By raising the temperature, it should initially populate the triplet state, causing the triplet EPR intensity to increase. However, if room temperature (300 K) is already at or above the temperature of maximum triplet intensity (kT ≈ ∣2J∣), further heating into the 300–360 K range places the spectrum on the downslope of the intensity curve, where the Curie law (1/T) thermal depopulation dominates over Boltzmann population. This implies a relatively weak antiferromagnetic coupling for complex **5b** compared to complex **5a**.

EPR spectra of complex **5c** with square planar geometry of both copper atoms exhibit at room temperature monomeric impurity at 3200 G, triplet Δm_s_ = ± 2 signal at 600 G (probably moderate ZFS), perpendicular triplet signal at 2900 G and two overlapping parallel triplet signals around 4500–5000 G. At low temperature (Figure 4), half field triplet signal shift to lower field 500 G, parallel B triplet components shift to almost 6000 G and perpendicular S = 1 component shift slightly left to 2800 G is observed. The signal at 3200 G (monomeric copper) just sharpens. At 100 K, the signal also decreases, suggesting a strong antiferromagnetic coupling and a singlet ground state. By increasing the temperature (Figure S1-17), middle components like the perpendicular triplet signal component and monomeric copper changed significantly, exhibiting thermal hysteresis, which may reflect a structural change or an order–disorder phase transition.

The EPR spectra of the three copper complexes reflect the relationship between structure and magnetic exchange. Complex **5a**, a polymeric complex with mixed square and pyramidal sites, exhibits coexisting distinct monomeric and triplet signals at room temperature. Cooling suppresses the triplet intensity, indicating an antiferromagnetic singlet ground state, while isolated monomers sharpen. Complex **5b**, a discrete asymmetric dimer, shows a full triplet manifold with resolved hyperfine structure at low temperatures, suggesting moderate coupling and structural flexibility influencing zero-field splitting. Complex **5c**, consisting of symmetric square-planar dimers, displays the strongest antiferromagnetic exchange. Square-planar geometry provides optimal orbital overlap for superexchange, resulting in the strongest antiferromagnetic coupling among the samples. Its triplet signals shift dramatically and lose intensity upon cooling, further confirming a robust singlet ground state. Symmetric square-planar arrangements typically facilitate stronger superexchange than mixed geometries, while polymeric structures introduce magnetic heterogeneity. Temperature-dependent intensity loss clearly confirms antiferromagnetic interactions, with structural symmetry correlating to exchange coupling strength. Geometric distortion significantly modulates zero-field splitting parameters, whereas connectivity determines final exchange magnitude.

### 2.4. DNA Interaction Studies

DNA is a major target of many anticancer drugs and is often a nonspecific target of cytotoxic compounds. Interference with DNA can disrupt transcription and replication, which are essential processes for cell growth and division [45]. Direct DNA binding is therefore considered a principal mechanism responsible for the antitumor activity of metal-based drugs [36].

In this study, the interaction of prepared reduced Schiff base complexes **5a–c** with calf thymus DNA was evaluated. We investigated the interaction between the complexes and DNA using several complementary techniques: UV-Vis absorption titrations, fluorescence emission with the ethidium bromide (EB) displacement method, viscosity studies, and thermal degradation of DNA.

#### 2.4.1. DNA Binding Study by Absorption Titration

The UV–Vis spectroscopic titration method compares the absorption spectra of the free metal complex with those obtained in the presence of DNA.

The absorption spectra of a fixed concentration of complexes **5a–c** (1 × 10^−4^ M) were detected in the presence of increasing amounts of ct-DNA (representatively shown for complex **5a** in Figure 5; spectra for **5b–c** can be found in Figure S4-1). In the UV–Vis spectra, changes in the intra-ligand absorption band were monitored, with absorption maxima at λ_max_ = 273 nm for all complexes; the methoxy group position did not affect λ_max_ in these measurements. This band intensity decreased from 18 to 24%. Table 5 shows the spectral absorption characteristics of the studied complexes.

Intercalation into DNA may shift the absorption maxima (bathochromic/red shifts or hypsochromic/blue shifts). In the absorption spectra of our complexes, no shifts in the absorption maxima were observed. This suggests that the complexes interact with DNA through groove binding rather than intercalation.

The DNA-binding constants (K*_b_*) of the complexes were calculated from the Wolfe–Shimer equation (Equation (1)) and respective plots (Figure 5 and Figure S4-1). The K*_b_* values are higher for complexes **5b** and **5c** (3.8–3.9 × 10^4^ M^−1^) than for **5a**. The K*_b_* values in 10^4^ M^−1^ range suggest strong binding of the complexes to DNA. However, these binding constants are lower than those typically observed for classical DNA intercalators (EB, K*_b_* = 1.23 × 10^5^) [46]. Published binuclear complexes of reduced Schiff bases derived from salicylaldehyde and aminopropanol exhibited comparable DNA-binding affinity (K*_b_* = 1.1–5.6 × 10^4^). Evaluation of their binding directly to nucleotides revealed that the complexes can bind to DNA through a coordination bond as well as within the DNA groove [37]. An example of stronger binding to DNA was reported in a series of copper(II) complexes with the reduced Schiff base ligand 2-(3,5-di-tert-butyl-2-hydroxybenzylamino)-2-benzyl-acetic acid) and heterocyclic co-ligands. The K*_b_* values of 1.3–3 × 10^5^ suggest that these metal complexes interact with ct-DNA by intercalation [14].

#### 2.4.2. Competitive Studies with EB-DNA

The ethidium bromide (EB) displacement method is often used to determine the ability of metal complexes to bind to DNA. EB is a typical DNA intercalator, and its insertion between adjacent DNA base pairs results in the formation of an EB-DNA adduct that shows an intense fluorescence emission in the range 590–620 nm when the solution is excited at 515 nm. Introducing a complex that has an affinity for DNA leads to a reduction in the emission intensity of the EB-DNA adduct. This occurs because the complex competes with ethidium bromide (EB) for the same binding sites on the DNA molecule, displacing EB and decreasing fluorescence. The addition of an increased concentration of the studied complexes led to a moderate decrease in the intensity of the emission band of the EB-DNA complex at 614 nm. Figure 6 shows the representative fluorescence emission spectra of **5a**. The quenching of EB-DNA fluorescence was evaluated by calculating the percentage decrease in fluorescence intensity (∆ I/I_0_)_,_ the Stern–Volmer quenching constant (*K_SV_*), and the quenching rate constant (*k_q_*).

The corresponding Stern–Volmer plots (Figure S4-2) provide clear evidence of the interaction between the tested complexes and DNA. The final quenching of the fluorescence reached 38–45% of the initial EB-DNA product (Figure 6, Table 6).

The calculated *K_SV_* constant (3.4–4.3 × 10^3^) confirmed the moderate activity of complexes **5a–c** for EB-DNA fluorescence quenching. The EB displacement ability of the studied reduced Schiff base complexes was mutually comparable; however, the best effect was achieved in the complex containing a methoxy substituent in the para position (**5c**). In addition, calculated values of the quenching rate constant *k*_q_ were higher than the value of 10^10^ M^−1^ s^−1^, indicating a static quenching mechanism that leads to the formation of a new adduct; i.e., between DNA and the complex [47].

For effective intercalators that displace EB from DNA and cause a significant reduction in the fluorescence intensity of the EB-DNA adduct, K*_SV_* values typically range from 10^4^ to 10^6^ M^−1^ [48]. Based on the *K_SV_* values obtained, the tested complexes can be classified as average groove binders, indicating relatively low affinity for DNA compared with classical intercalators. The moderate intercalative mode (K*_SV_* = 2.8 − 4.13 × 10^4^ M^−1^) was described in structurally similar copper(II) complexes of a reduced Schiff base ligand derived from 4-hydroxybenzaldehyde and glycine [49]. The strong intercalation ability into DNA was exhibited by complexes containing a reduced Schiff base based on benzene-1,4-dicarboxaldehyde and 2-methylalanine ligand with *N*-donor heterocyclic base auxiliary ligands (phen, dpq, dppz), with K*_SV_* values on the order of 10^5^ [11].

#### 2.4.3. Viscosity Measurements

Viscosity measurements further verify the interaction mode of metal complexes with ct-DNA. When molecules bind through intercalation, they insert between DNA base pairs, increasing the helix length and, consequently, the viscosity. In contrast, non-classical modes of interaction such as groove-binding and electrostatic interactions can bend or kink the DNA structure, reducing its length and viscosity [47].

The viscosity measurements of DNA solutions were carried out in the presence of increasing concentrations of complexes (Figure 7). Adding complexes **5a–c** resulted in only a slight increase or no significant change in DNA viscosity. These findings indicated that all complexes may interact with DNA predominantly through a groove-binding mode.

#### 2.4.4. Thermal Denaturation Studies

DNA denaturation refers to the separation of double-stranded calf thymus DNA (ct-DNA) into two single strands. The temperature at which 50% of the DNA duplex converts to single-stranded DNA is known as the melting temperature (T_m_). The T_m_ value is typically determined from the midpoint of the DNA melting curve obtained through spectroscopic measurements.

In this study, we determined the melting temperature of ct-DNA by monitoring its absorbance at 260 nm over a temperature range of 40–95 °C. Figure 8 shows the behavior of thermal denaturation of the ct-DNA solutions in the absence and presence of three complexes.

The T_m_ value, determined from the first derivative of the denaturation curve, was 68.5 °C for DNA under our experimental conditions. The T_m_ values of ct-DNA increased by 2.6, 1.02, and 2.2 °C in the presence of complexes **5a**, **5b**, and **5c**, respectively, indicating that these complexes can stabilize the DNA double helix. Nevertheless, the literature reports that typical intercalators generally produce a much larger increase in DNA melting temperature (ΔT_m_). In contrast, non-intercalative binding modes, such as groove binding or external/electrostatic association with DNA, are characterized by lower ΔT_m_ values (<3 °C) [50]. Therefore, the relatively small T_m_ changes observed in this study suggest that the studied complexes interact with DNA predominantly through groove or external binding rather than intercalation. These findings agree well with the viscosity measurement results.

### 2.5. DNA Cleavage

The cleaving ability of copper complexes can be determined by the standard gel electrophoresis method [51]. The gel electrophoresis separation of supercoiled DNA plasmid pBR322 induced by the prepared copper(II) complexes is shown in Figure 9. All three copper(II) complexes (**5a–c**) cleaved the supercoiled form of DNA (Form I) to an open-circular, partly cleaved form (Form II) and showed various DNAse activities.

The concentration range of the prepared complexes (1–5 × 10^−3^ M) was motivated by several considerations. Similar Schiff base complexes have previously been reported to exhibit moderate DNA-cleaving activity rather than highly efficient artificial nucleases. Consequently, detectable DNA cleavage is often observed only at millimolar concentrations [44,52,53]. In the present study, the DNA cleavage activity of our complexes was evaluated in the absence of auxiliary reagents that typically promote oxidative DNA cleavage, such as ascorbate, hydrogen peroxide, thiols, or other reducing agents. Without these additives, DNA cleavage is generally limited and therefore requires higher complex concentrations to achieve detectable effects. As groove binders, these complexes may efficiently induce hydrolytic DNA cleavage. When bound within the minor groove, they are positioned sufficiently close to facilitate the formation of a coordination interaction between the copper center and the hydroxylated ligands from nucleic acid [54]. Moreover, the metal center may act as a Lewis acid and promote phosphodiester hydrolysis [55].

Alternatively, a redox-active copper center may promote in situ ROS formation or oxidative cleavage [56,57,58,59]. When the complexes are located in the minor groove, any ROS generated are very near the DNA molecule, and cleavage can occur even though the complex is only groove-bound [60]. However, the Cu complexes often exhibit antioxidant or antiradical behavior, suggesting relatively low ROS production. Increasing the concentration might increase the total amount of ROS generated and, therefore, the observed cleavage [61]. This may explain why the cleavage activity of complex **5a** increased markedly at 5 × 10^−3^ M (Figure 9).

The DNA cleaving activities were quantified using gelQUANT software (version 4.3.9, Cleaver Scientific, Rugby, UK). The data are expressed as the percentage (%) of the amount of pDNA that has passed through the effect of the nuclease activity of the copper complex from the uncleaved supercoiled form I to the cleaved form II (open circular pDNA) (Table 7). Each applied sample was 20 μL and contained 250 ng of pDNA. Therefore, each percentage can also be converted to the mass of pDNA converted to form II. For example, for complex **5c** at 1 × 10^−3^ M, 26.4% of pDNA was cleaved to form II (66 ng of pDNA); at 3 × 10^−3^ M, it was 30.2% (75.5 ng of pDNA). The quantification allows us to compare the specific complexes more precisely. As was obvious from the electrophoresis images, complex **5a** showed the best activity at 5 × 10^−3^ M (94.5% cleavage to form II). The activities of complexes **5a** and **5c** at 1 × 10^−3^ M and of all three complexes at 3 × 10^−3^ M were in a similar range (24–31%), but at 5 × 10^−3^ M major differences were evident, with the order **5a** > **5c** > **5b**.

### 2.6. Interactions with Bovine Serum Albumin (BSA)

Understanding how a new drug interacts with serum albumins is a critical step in drug development. Albumins significantly influence its transport, distribution, metabolism, and excretion. Due to its strong structural similarity to human serum albumin, bovine serum albumin (BSA) is commonly used as a model system for studying intermolecular interactions between drugs and proteins [62].

Fluorescence spectroscopy provides an efficient approach to investigate metal-complex interactions with BSA. BSA’s intrinsic fluorescence originates from its aromatic amino acid residues; namely, tryptophan, tyrosine, and phenylalanine. Because tryptophan possesses a substantially higher fluorescence quantum yield than the other residues, BSA fluorescence is mainly attributed to Trp-134 and Trp-213 located in subdomains IB and IIA, respectively [63].

In our study, the fluorescence spectra of BSA were recorded in the absence and presence of increasing concentrations of the studied metal complexes. The fluorescence quenching of BSA was monitored at the maximum emission (332–336 nm) upon excitation at 280 nm. The resulting quenching data were evaluated by Stern–Volmer constants (*K_SV_*), quenching rate constant (*k*_q_) (Figure S5-1), binding constants (*K_BSA_*), and the number of binding sites (*n*) determined from Scatchard equations (Equation (S5-2), Figures S5-2 and S5-3). The values are summarized in Table 8. Figure 10 and Figure S5-1 show changes in the BSA emission spectra.

The fluorescence quenching of BSA with complexes reached 35–37%. The *K_SV_* values (2.3–2.9 × 10^4^ M^−1^) suggest a moderately strong affinity for BSA. However, the presented values of the quenching constant *k*_q_ of the complexes (10^12^ M^−1^s^−1^) significantly exceed 10^10^ M^−1^s^−1^—the maximum scatter collision quenching constant of various quenchers with biopolymers, indicating a static quenching mechanism [64].

In terms of binding affinity and fluorescence quenching, complex **5b** was less effective than complexes **5a** and **5c**, which exhibited comparable activities.

The calculated binding constant *K_BSA_* values for the studied complexes (10^4^ M^−1^) indicate an affinity that is considered favorable for serum albumin-mediated drug delivery (with *K_SV_* within 10^2^–10^8^; binding constant *K_BSA_* within 10^4^–10^6^) [65]. Similar binding constants and *K_SV_* values (10^4^–10^5^ M^−1^, occasionally 10^6^ M^−1^) were reported for copper complexes with various ligand structures [24,44,52,57].

### 2.7. Cytotoxicity Based on Mitochondrial Activity

The resazurin-based model that monitors mitochondrial activity is widely used as an indicator of cell viability, proliferation, and cytotoxicity. This method is based on the reduction of resazurin to resorufin by mitochondrial enzymes, such as NADPH dehydrogenase, which transfers electrons from NADPH+ and H+ to resazurin. Resazurin reduction correlates with the number of live bacterial, mammalian, or fungal cells. Furthermore, *S. cerevisiae* is an established model system for eukaryotic organisms [66,67,68]. Of course, yeast cannot fully replace mammalian cells because mammals have a more complex cellular environment and more complicated genetic interactions [69]. For this reason, concentrations used across toxicity models vary. Each cellular model, whether derived from yeast or human cells, may show different sensitivity to the tested compounds, making it difficult to predict effects on specific cell types. However, they share conserved cellular processes with high homology to human cell lines. They proliferate rapidly and are even used as a model to study the effects of anticancer agents [70]. Using *S. cerevisiae* in the resazurin redox test is a good alternative to conventional cell viability testing techniques.

The absorbance values obtained for reduced and oxidized resazurin (measured at 570 nm and 600 nm) are listed in Table 9. The reduction percentage was calculated using Equation (7). The complexes showed excellent cytotoxic activity ranging from 92 to 98% depending on the concentration of the added complex (Table 9, Figures S6-2, S6-3 and S6-4). Complexes **5a–c** are already highly effective in cytotoxicity at a 1 × 10^−4^ M concentration and behave very similarly.

### 2.8. Cytotoxicity Against Cancer Cells Measured by MTT Tests

The cytotoxicity of the prepared copper(II) complexes was assessed using the MTT assay on human cell lines: A549 (human alveolar basal epithelial adenocarcinoma), HepG2 (human liver cancer), and HEL (human lung fibroblast, normal). Cytotoxicity was measured across various sample concentrations at 24 and 72 h; however, cytotoxicity was poorly observable at 24 h but became apparent at 72 h, especially against HepG2 cells. We calculated the IC_50_ values based on the measured data for all prepared complexes and the standard cisplatin (Figure 11 and Table 10). The IC_50_ profiles of all three complexes show the best activity against HepG2 cells, while the IC_50_ values against A549 and HEL are somewhat similar. Complex **5b** showed the lowest IC_50_ against HepG2 (33.0 ± 5.3 μM), but it was much higher against HEL cells (81.7 ± 3.3 μM), with a selectivity index (SI = IC_50_ HEL/IC_50_ HepG2) of 2.48. An even more remarkable difference between the IC_50_ of HepG2 and HEL cells can be attributed to complex **5a** (44.7 ± 6.4 μM vs. 136.5 ± 16.8 μM, SI = 3.05). Complex **5c** also maintains this trend while being less selective (42.4 ± 4.7 μM vs. 88.4 ± 16.3 μM, SI = 2.08). Cisplatin, however, remained more active and selective not only against HepG2 (SI = 4.28) but also against A549 cells (SI = 2.61), even though the IC_50_ against HEL is also low. By contrast, the complexes have much higher IC_50_ values against A549 cells than cisplatin.

The high cytotoxicity of complex **5b** might seem unusual compared with the DNA cleavage results. The DNA cleavage data highlight complex **5a**, but only at 5 × 10^−3^ M. At lower concentrations, the complexes achieve similar DNA cleavage percentages. This may reflect a different mechanism of action or a combination of mechanisms. Complex **5b** has the best binding constant (*K_b_* = 3.90 × 10^4^) determined by absorption titration, but the EB displacement is lowest. Thus, although DNA cleavage is limited, its DNA-binding ability could be linked to cytotoxicity by preventing or slowing DNA replication or transcription. Copper complexes of Schiff bases and other ligands that bind/cleave DNA have been shown to halt the cell cycle and induce apoptosis [71,72], as detected by changes in anti-/pro-apoptotic proteins such as Bcl-2 or Bax [73].

Compared to the high variability of activities within the MTT test, the resazurin assay monitoring mitochondrial activity in *S. cerevisiae* cells shows that all three complexes behave similarly (around 95% cell death at 1 × 10^−4^ M). So, another alternative pathway could involve mitochondrial activity [74]. Compared with pDNA and ct-DNA studies, the cytotoxicity experiments (resazurin and MTT assays) involve more complicated models with living cells. Activity is affected by cellular uptake processes driven by membrane permeability, as well as by intracellular processes and binding to alternative targets such as proteins. Accumulation of copper complexes in cells can lead to copper-induced cell death (cuproptosis), which is associated with mitochondrial damage and halting/slowing the citric acid cycle. Copper can do this by binding to specialized sulfur-containing proteins or lipoylated proteins in the tricarboxylic acid (TCA) cycle, resulting in protein aggregation, loss of iron–sulfur cluster proteins, proteotoxic stress, and ultimately cell death. Mitochondrial damage is related to mitochondrial membrane potential loss. This causes mitochondrial morphological changes and halts mitochondrial processes. This again leads to cell death [75,76,77,78,79].

Copper complexes are also widely studied as radical scavengers, but as they accumulate in a cell, they can generate ROS, increasing oxidative stress and interfering with cellular metabolism [80]. The ability of complexes to release/absorb one electron while copper changes oxidation state between II and I (SOD mimetic activity) was therefore another interesting study aim. This process can be studied using radical scavenging models capable of giving or taking one electron to/from an SOD mimetic [29,81].

### 2.9. SOD Mimetic Activity

The radical scavenging ability of the prepared complexes was determined by the INT method using a tetrazolium dye. This method is based on a competitive reaction between the measured compound (complex) and 2-(4-iodophenyl)-3-(4-nitrophenyl)-5-phenyl-2H-tetrazolium chloride (INT) with the superoxide anion radical (from KO_2_). INT reacts with the single electron from O_2_^−•^ and produces blue-green formazan, detected as light absorption at 500 nm. Copper complexes with good SOD mimetic (radical scavenging) activity also react with O_2_^−•^, and as a result of this, absorption at 500 nm is decreased.

The first antiradical activity measurement was the determination of electron transfer inhibition percentage on INT at specific concentrations (Table 11). We selected two initial solution concentrations of the complexes in DMSO (4 × 10^−3^ M and 1 × 10^−3^ M). The initial solutions (0.5 mL) were mixed with the other agents (INT and KO_2_ solution) and additional DMSO to a final volume of 3.5 mL. The resulting concentrations were c_1_ = 5.72 × 10^−4^ M and c_2_ = 1.43 × 10^−4^ M. Complex **5b** showed the best percentages at c_1_ (around 96%) and at c_2_ (around 24%). Complexes **5a** and **5c** showed lower but still relatively high activity at c_1_ (around 74–81%). The complexes were compared with the model compound cystamine, which was used as a standard for superoxide antiradical scavenging because of its free thionyl group, which is also present in glutathione.

To improve the comparability of the SOD mimetic activity, SOD-IC_50_ concentrations were determined by performing a series of measurements with decreasing concentrations (Table 11, Figure S7-1). SOD-IC_50_ values confirmed that complex **5b** was the most effective radical scavenger, followed closely by **5c**. Complex **5a** is somewhat worse than the other two, which might be caused by steric hindrance of the ortho-methoxy group from approaching the copper center.

### 2.10. SAR of the Prepared Complexes

In DNA binding experiments, complexes **5b** and **5c** show better binding constants K*_b_* = 3.84–3.90 × 10^4^ M^−1^ in the absorption titration method, and complex **5c** was best in the EB displacement assay (Stern–Volmer quenching constant *K_SV_* = 4.3 × 10^3^ M^−1^ with 43.4% fluorescence quenching). In contrast, DNA cleavage studies highlight complex **5a**, but only at the highest concentration (5 × 10^−3^ M). The activities of complexes **5a** and **5c** at 1 × 10^−3^ M and of all three complexes at 3 × 10^−3^ M were in a similar range (24–31%). Overall, the DNA cleavage activity order would be **5a** > **5c** > **5b**.

The cytotoxicity studies monitoring mitochondrial activity of *S. cerevisiae* cells show that all three complexes behave similarly, with activities ranging around 95% at 1 × 10^−4^ M. But the MTT assays revealed large differences in activities, with **5b** reaching the lowest IC_50_ values against HepG2 and A549 cells, and the best HEL/HepG2 selectivity index of 3.05 was found for complex **5a**. The cytotoxicity of **5b** seems inconsistent with the DNA cleavage measurements but agrees with DNA binding to ct-DNA. The studied complexes seem to rely on different mechanisms of action or a combination of mechanisms. For example, **5b** and **5c** bind DNA better, while **5a** cleaves DNA more effectively. At the cellular level, cytotoxic effects are influenced by differences in uptake ability across cell lines. Intracellular processes, such as mitochondrial activity, or alternative biological targets, such as proteins linked to the TCA cycle, may also drive differences in cytotoxicity responses.

Comparison of the radical scavenging results with cytotoxicity showed **5b** as the best superoxide radical scavenger, followed by **5c**, and **5a** was the least active. What is important to note is that radical scavenging is linked to the ability to absorb and release one electron from or to a reactive species, so a reduction of the superoxide (O_2_^−•^) can occur to produce a peroxide anion (O_2_^2−^), and the oxidation can form an oxygen molecule (O_2_). So, just as SOD enzymes do, copper complexes should be able to undergo Cu^II^ to Cu^I^ and back redox reactions. This may not only remove radicals from mixtures but also produce them under the right conditions. In our case, DNA cleavage may also be affected by the SOD-mimetic activity of the complexes, with the worst superoxide scavenger, **5a**, being the best at DNA cleavage. This was mainly visible at 5 × 10^−3^ M, where accumulation near DNA can lead to effective DNA cleavage.

Overall, choosing one of the three complexes is not possible because each showed different measured activities. Complexes **5b** and **5c** might cause cytotoxicity probably through DNA binding, while **5a** is the best DNA cleaver. In contrast, **5b** appears to be the best radical scavenger and affects oxidative stress in the cell more than the other two complexes. Other mechanisms of action, such as interference with mitochondrial function, as suggested by the resazurin and MTT cytotoxicity assays, are also possible.

## 3. Materials and Methods

### 3.1. Instrumentation and Materials Used in Synthesis

All chemicals for synthesis were reagent grade and were used as received from Sigma-Aldrich (Merck, Darmstadt, Germany), Acros Organics (Thermo Fisher Scientific Inc., Waltham, MA, USA), Merck (Merck, Darmstadt, Germany), and Alfa-Aesar (Thermo Fisher Scientific Inc., Waltham, MA, USA), apart from methanol, which was dried in a three-step approach: predrying with calcium oxide, reflux with magnesium activated by iodine for 2–3 h, and distillation into dried 4Å molecular sieves.

All NMR spectra were measured on a Varian Gemini 2000 spectrometer (Varian Medical Systems, Palo Alto, CA, USA) at working frequencies of 300 MHz for ^1^H NMR and 75 MHz for ^13^C NMR. Spectra were measured in CDCl_3_, using TMS as an internal standard. For the NMR signal assignment, carbons 1–6 are located on the phenolic ring (Ar), with 1 being the carbon where the aminomethyl group is located; 7–8 belong to the two carbons in the linker between the aromatic rings; 9 is the methoxy group; and the methoxyphenyl has its own numbering (1′–6′, Ar’). Infrared spectra were recorded on a Nicolet 6700 FT-IR spectrophotometer (Thermo Fisher Scientific Inc., USA) in the range 500–4000 cm^−1^, and the samples were in solid or oily form. Elemental analysis was measured using the Flash 2000 CHNS-O analyzer (Thermo Fisher Scientific Inc., Waltham, MA, USA). UV-Vis spectra were measured in DMSO using a Genesys 10S UV-Vis spectrometer (Thermo Fisher Scientific Inc., Waltham, MA, USA). The melting point was determined on a Koffler block equipped with a microscope (Thermo Fisher Scientific Inc., Waltham, MA, USA). HRMS spectra for stability studies were measured on an Orbitrap LTQ XL (Thermo Fisher Scientific Inc., Waltham, MA, USA).

### 3.2. General Procedure for Synthesis of the Ligands (**4a–c**)

In a two-neck flask equipped with a reflux condenser and a septum, a mixture of 2-, 3-, or 4-methoxybenzylamine (**2a–c**) (0.66 g, 4.79 mmol, 1 mol equiv) in 20 mL of dry methanol was prepared. The solution of salicylaldehyde (**1**) (0.59 g, 4.79 mmol, 1 mol equiv) in 5 mL of dry methanol was added through the septum, and the entire reaction mixture was heated under reflux while monitored by TLC (hexane:ethyl acetate = 19:1). After 1–2 h, the reaction reached completion, producing intermediate Schiff bases **3a–c**, which were not isolated. The mixture was cooled down to rt. Solid NaBH_4_ (0.22 g, 5.75 mmol, 1.2 mol equiv) was added, and the mixture was stirred for 1 h at rt. The color changed from yellow to white/colorless. Methanol was evaporated, and the solid was extracted into CHCl_3_ (three times, 15 mL) to remove inorganic impurities. CHCl_3_ was evaporated to afford a beige solid or oil. The product was purified by flash chromatography on a short silica gel column (eluent mixture:hexane:ethyl acetate = 19:1).

**2-[(2-Methoxy-benzylamino)-methyl]-phenol (4a, L^1^)** was prepared as a colorless oil weighing 1.10 g (94%). ^1^H NMR (300 MHz, CDCl_3_, ppm) δ: 7.26 (ddd,1H, *J*(3′,4′) = 8.9 Hz, *J*(4′,5′) = 7.6 Hz, *J*(4′,6′) = 1.0 Hz, C4′-H), 7.17 (ddd, 1H, *J* = 7.7 Hz, *J* = 7.2 Hz, *J*(4,6) = 1.3 Hz, C4-H), 6.97 (dd, 1H, *J*(5′,6′) = 7.2 Hz, *J*(4′,6′) = 1.0 Hz, C6′-H), 6.82–6.89 (m, 4H, C3-H, C5-H, C6-H, C3′-H), 6.77 (dd, 1H, *J*(5′,6′) = 7.6 Hz, *J*(4′,5′) = 7.2 Hz, C5′-H), 3.99 (s, 2H, C7-H_2_ or C8-H_2_), 3.81 (s, 3H, C9-H_3_), 3.78 (s, 2H, C7-H_2_ or C8-H_2_). ^13^C NMR (75 MHz, CDCl_3_, ppm) δ: 159.9 (C2′), 158.2 (C2), 129.7 (C6 and C6′), 128.8 and 128.5 (C4 and C4′), 122.2 (C1), 120.6 (C5), 119.0 (C1′), 116.4 (C5′), 113.9 and 112.9 (C3 and C3′), 55.2 (C9), 52.5 and 51.8 (C7 and C8). Elemental analysis calculated for C_15_H_17_NO_2_ (M_r_ = 243.30): C 74.05; H 7.04; N 5.76%; measured: C 73.85; H 7.14; N 5.71%. IR (solid, cm^−1^): 3342 (w, N-H), 3009 (w, C-H), 2939 (w, C-H), 2838 (w, CH_2_), 1589 (s, N-H), 1493 (s, C-N), 1473 (m, C-H), 1462 (m, C-H), 1435 (w), 1416 (m, C-N), 1345 (w), 1288 (w), 1259 (s, C-O), 1245 (s, C-O), 1179 (w), 1152 (w), 1119 (m), 1092 (m, C-N), 1084 (w), 1031 (s, C-O), 975 (m, C-H), 936 (w), 863 (m), 838 (s, C-H), 801 (m), 755 (s, C-H), 735 (w), 607 (m).

**2-[(3-Methoxy-benzylamino)-methyl]-phenol (4b, L^2^)** was obtained as an almost colorless oil weighing 1.09 g (93%). ^1^H NMR (300 MHz, CDCl_3_, ppm) δ: 7.28 (ddd, 1H, *J* = 7.9 Hz, *J* = 7.6 Hz, *J* = 1.3 Hz, Ar), 7.11–7.16 (m, 2H, Ar + Ar′), 6.91–6.96 (m, 2H, Ar + Ar′), 6.87 (d, 1H, *J* = 8.6 Hz, Ar′), 6.80 (d, 1H, *J* = 7.6 Hz, Ar′), 6.73 (dd, 1H, *J* = 7.6 Hz, *J* = 7.3 Hz, Ar), 3.98 (s, 2H, C7-H_2_ or C8-H_2_), 3.81 (bs, 5H, C9-H_3_ and C7-H_2_ or C8-H_2_). ^13^C NMR (75 MHz, CDCl_3_, ppm) δ: 159.0 (C4′), 157.8 (C2), 130.6 a 129.0 (C6 a C5′), 128.62 (C4), 128.56 (C1′), 126.4 (C5), 120.4 (C6′), 118.5 (C1), 116.5 (C3), 112.7 (C2′), 110.4 (C4′), 55.2 (C9), 51.5 (C8), 48.3 (C7). Elemental analysis calculated for C_15_H_17_NO_2_ (M_r_ = 243.30): C 74.05; H 7.04; N 5.76%; measured: C 73.79; H 7.19; N 5.59%. IR (solid, cm^−1^): 3319 (w, N-H), 3004 (w, C-H), 2835 (m, C-H), 1586 (s, N-H), 1489 (s, C-N), 1457 (m, C-H), 1437 (w), 1411 (w), 1257 (s, C-O, both), 1187 (w), 1153 (s, C-N), 1085 (w), 1038 (m, C-O), 994 (w), 931 (w), 846 (w), 781 (w), 754 (s, C-H), 720 (w), 694 (s), 611 (w).

**2-[(4-Methoxy-benzylamino)-methyl]-phenol (4c, L^3^)** was obtained as an oil that turned into a white solid upon standing at room temperature weighing 1.15 g (98%). M.p. 65–67 °C. ^1^H NMR (300 MHz, CDCl_3_, ppm) δ: 7.14–7.22 (m, 3H, C4-H, C2′-H a C6′-H), 6.91 (dd, 1H, *J*(5,6) = 7.6 Hz, *J*(4,6) = 1.3 Hz, C6-H), 6.83–6.90 (m, 3H, C3-H, C5′-H, C3′-H), 6.77 (ddd, 1H, *J*(4,5 and 5,6) = 7.6 and 7.3 Hz, *J*(3,5) = 1.0 Hz, C5-H), 3.98 (s, 2H, C7-H_2_ or C8-H_2_), 3.80 (s, 3H, C9-H_3_), 3.75 (s, 2H, C7-H_2_ or C8-H_2_). ^13^C NMR (75 MHz, CDCl_3_, ppm) δ: 159.0 (C4′), 158.2 (C2), 130.5 (C1′), 129.6 (C2′ a C6′), 128.7 and 128.4 (C4 and C6), 122.3 (C1), 119.0 (C5), 116.4 (C3), 114.6 (C3′ and C5′), 55.3 (C9), 51.9 and 51.7 (C7 and C8). Elemental analysis calculated for C_15_H_17_NO_2_ (Mr = 243.30): C 74.05; H 7.04; N 5.76%; measured: C 73.71; H 7.24; N 5.66%. IR (solid, cm^−1^): 3322 (s, N-H), 3075 (w, C-H), 3005 (m, C-H), 2937 (w), 2907 (w), 2841 (w), 1590 (m, N-H), 1510 (s, C-N), 1475 (m), 1463 (s, C-H), 1426 (m), 1417 (w), 1360 (w), 1304 (w), 1260 (s, C-O), 1242 (s, C-O), 1230 (m), 1174 (m, C-O), 1155 (w), 1115 (w), 1104 (w), 1088 (s, C-N), 1051 (w), 1026 (s, C-O), 985 (s, C-H), 947 (w), 918 (w), 827 (m), 777 (s, C-H), 759 (m), 719 (w), 639 (w), 620 (s).

### 3.3. General Procedure of Synthesis of Copper(II) Complexes (**5a–c**)

The solution of Cu(OAc)_2_ ∙ H_2_O (164 mg, 0.822 mmol, 1 mol equiv) in 20 mL of methanol was prepared by slight heating and filtration and added to the solution of the reduced Schiff base ligand **4a–c** (200 mg, 0.822 mmol, 1 mol equiv) in 10 mL of methanol. The mixture was heated at around 50–60 °C for another 10–15 min and left to cool to rt. The product precipitated from solution after 1–2 h. The product was isolated by vacuum filtration and washed with diethyl ether. The complexes were recrystallized from a methanol/water mixture by allowing it to stand at rt for 1–3 days with enabled evaporation. The dark green crystals were isolated by vacuum filtration.

**[Cu_2_ L^1^_2_ (AcO)_2_]_n_ (5a)** was isolated as a green powder in 140 mg (47%). Elemental analysis calculated for the binuclear complex C_34_H_38_Cu_2_N_2_O_8_ (M_r_ = 729.76): C 55.96; H 5.25; N 3.84%; measured: C 55.67; H 5.06; N 3.64%. IR (solid, cm^−1^): 3298 (w), 3194 (s, N-H), 3011 (w), 2951 (w), 2837 (w), 1599 (w), 1587 (m, N-H), 1561 (s, COO), 1483 (s, C-N), 1452 (m), 1438 (m), 1397 (s, COO), 1355 (w), 1336 (m, C-O), 1271 (s, C-O), 1247 (s), 1148 (w), 1113 (w), 1086 (w), 1050 (w), 1033 (s, C-O), 1006 (w), 933 (m), 920 (w), 886 (m), 881 (w), 860 (w), 846 (w), 782 (w), 751 (s, C-H), 738 (m), 726 (m), 681 (s, Cu-O), 609 (m, Cu-N). UV-Vis (nm): 258, 278, 398, 721.

**[Cu_2_ L^2^_2_ (AcO)_2_] ∙ 0.25 H_2_O (5b)** was obtained as a green powder in 147 mg (49%). Elemental analysis calculated for C_34_H_38,5_Cu_2_N_2_O_8,25_ (M_r_ = 734.25): C 55.61; H 5.28; N 3.82%; measured: C 55.28; H 5.34; N 3.54%. IR (solid, cm^−1^): 3442 (s, coord.water), 3231 (s, N-H), 3016 (w), 2933 (m), 2833 (w), 1629 (s), 1598 (m, sec. N-H), 1560 (s, COO), 1481 (s), 1468 (w), 1420 (s, COO), 1343 (m, C-O), 1295 (w), 1269 (w), 1256 (s, C-O), 1238 (w), 1171 (m), 1152 (w), 1110 (m), 1081 (w), 1048 (m), 1029 (s), 952 (w), 932 (w), 894 (m), 881 (m), 847 (m), 787 (m), 752 (s, C-H), 734 (w), 683 (s, Cu-O), 671 (w), 627 (m, Cu-N). UV-Vis (nm): 261, 279, 402, 709.

**[Cu_2_ L^3^_2_ (AcO)_2_] (5c)** was obtained as a green powder in 246 mg (82%). Elemental analysis calculated for C_34_H_38_Cu_2_N_2_O_8_ (Mr = 729.76): C 55.96; H 5.25; N 3.84%; measured: C 55.99; H 5.25; N 3.75%. IR (solid, cm^−1^): 3162 (m, N-H), 3007 (w), 2936 (w), 2868 (w), 2839 (w), 1611 (w), 1599 (m, N-H), 1563 (s, COO), 1512 (s), 1485 (m), 1444 (m), 1427 (m, COO), 1412 (w), 1341 (m, C-O), 1302 (w), 1264 (m), 1251 (s, C-O), 1183 (w), 1176 (w), 1127 (w), 1114 (m), 1031 (s, C-O), 936 (m), 924 (m), 888 (m), 872 (m), 845 (w), 834 (w), 816 (m), 781 (m), 753 (s, C-H), 731 (s), 683 (s, Cu-O), 641 (w), 623 (s, Cu-N). UV-Vis (nm): 262, 279, 398, 715.

### 3.4. Stability Studies in Solutions

The stability of the complexes was studied using UV-Vis spectral studies. All prepared complexes were sufficiently soluble for all measurements. First, the solutions of the complexes in DMSO and aqueous citrate buffer solution were studied for up to 72 h (Figures S1-3 to S1-8). We conducted UV-Vis spectroscopic studies to confirm complex formation by comparing the spectra of the two separate solutions containing the free ligand in one and the copper salt in the second cuvette (placed together in the detection area) with the complex solution in both cuvettes after mixing and after 1 h. This showed that ligand binding to copper also occurred in DMSO (Figure S1-9). MS studies of the dissolved complexes revealed that, as expected, the complexes dissociate in solution to mononuclear species (Figures S1-10 to S1-12).

### 3.5. X-Ray Structure Analysis

Diffraction measurements for all complexes were performed on a STOE STADIVARI diffractometer (STOE & Cie GmbH, Darmstadt, Germany) equipped with a Dectris Pilatus 300 K detector, with a Genix3D Cu HF source (Cu-Kα, λ = 1.54186 Å) at 100 K using a nitrogen gas open-flow cooler Cobra (Oxford Cryosystems, Long Hanborough, UK). Data reduction was achieved using the X-Area software package (version 1.84.1, STOE & Cie GmbH, Darmstadt, Germany). The crystal structures of complexes **5a–c** were solved in OLEX2 software (version 1.5) [82] using the SHELXT-2015 program via Intrinsic Phasing [83] and refined with SHELXL-2015 by a least-squares procedure on F^2^ [84]. All non-hydrogen atoms were refined with anisotropic thermal parameters. The positions of all H atoms were optimized under the constraint of riding on their parent atoms, with C–H (aromatic) bond length of 0.95 Å, C–H (aliphatic) bond length of 0.99 Å, C–H(methyl group) bond length of 0.98Å, N–H(amine group) bond length of 1.00Å with hydrogen O–H bond length of 0.89Å. Hydrogen temperature factors are Uiso(H) = 1.2 Ueq(C) for the methyl group, Uiso(H) = 1.5 Ueq(C) for the methyl group, and Uiso(H) = 1.5 Ueq(O) for the water molecule. The DIAMOND program (version 2.1e) [85] and MERCURY program (version 4.1) [86] were used for the molecular graphics.

#### Accession Codes

CCDC 2367652, 2576586 and 2576587 contain the supplementary crystallographic data for this paper. These data can be obtained free of charge via www.ccdc.cam.ac.uk/data_request/cif (accessed on 25 September 2026), or by e-mailing data_request@ccdc.cam.ac.uk, or by contacting The Cambridge Crystallographic Data Centre, 12 Union Road, Cambridge CB2 1EZ, UK; fax: +44 1223 336033.

### 3.6. EPR Spectra Measurement

EPR spectra were recorded using an X-band CW EPR spectrometer (Bruker EMX Plus; Bruker, Mannheim, Germany) with an ER4116DM dual-mode resonator (version 2.11; Bruker, Mannheim, Germany) that can operate in both TE102 mode (perpendicular) and TE012 mode (parallel). Most of the measurements were performed in TE102 perpendicular mode. The instrument was operated using Bruker Acquisition proprietary software(version 2.11). For elevated-temperature measurements, we used a standard Bruker temperature unit. Measurements at low temperatures were performed in boiling nitrogen (77K) in a Dewar finger, using a nitrogen gas evaporator and temperature unit setup from Bruker. For solid-state measurements, a small amount of solid-state powder was placed into a thin-wall quartz EPR tube (internal diameter 3 mm).

### 3.7. DNA Cleavage Assay

The ability of copper(II) complexes to cleave DNA was assessed by monitoring the conversion of supercoiled plasmid DNA (Form I) to open-circular DNA (Form II) by agarose gel electrophoresis to separate the cleavage products [87,88].

In general, 250 ng of pBBR 322 DNA plasmid (in 2.5 × 10^−2^ M Tris-HCl buffer solution, pH 7.5) was treated with different concentrations (1 × 10^−3^, 3 × 10^−3^, 5 × 10^−3^ M) of the copper(II) complex (dissolved in DMSO) for 3 h at 37 °C. After that, each reaction was quenched by adding 4 μL of a Smart Glow Loading Dye^®^ (loading buffer solution with 0.01% Safe Green dye, 50% glycerol, and 0.25 M EDTA, pH 8.0) and the sample subjected to electrophoresis (MyGel Mini electrophoresis system^®^, Accuris Instruments, Edison, NJ, USA) on a 0.9% agarose gel containing a TAE buffer (4 × 10^−2^ M Tris, 2 × 10^−2^ M acetic acid, and 1 × 10^−3^ M EDTA) at a voltage of 55 V/40 mA for approximately 1.5 h. As a “running buffer”, TAE buffer (4 × 10^−2^ M Tris, 2 × 10^−2^ M acetic acid, and 1 × 10^−3^ M EDTA) was used. The resulting gels were visualized under UV light and scanned with a mobile device, an Apple iPhone 11 cam 26 mm f1.8. The DNA cleavage activities were quantified using gelQUANT software (version 4.3.9, Cleaver Scientific, Rugby, UK).

### 3.8. DNA Binding Study by Absorption Titration

The interactions of prepared complexes **5a–c** with ct-DNA were studied by UV-Vis-monitored titration. Measurements were performed in citrate buffer (1.5 × 10^−3^ M sodium citrate and 0.15 M NaCl at pH 7.0) at room temperature. The Cu(II) complexes were dissolved in DMSO, from which working solutions were prepared by diluting with citrate buffer at a concentration of 1 × 10^−4^ M. Increasing concentrations of ct-DNA were then added to a buffer/DMSO solution (<1% DMSO) of the corresponding complex [89].

For the values of the binding strength of the interaction between the complex and the DNA molecule, binding constants K*_b_* were calculated by the ratio of the slope to the y-intercept in plots of [DNA]/(ε_A_ − ε_f_) versus [DNA], according to the Wolfe–Shimer equation (Equation (1)):

(1) $$\frac{\left[DNA\right]}{\left(\varepsilon_{A}-\varepsilon_{f}\right)}=\frac{\left[DNA\right]}{\left(\varepsilon_{b}-\varepsilon_{f}\right)}+\frac{1}{K_{b}(\varepsilon_{b}-\varepsilon_{f})}$$

where [DNA] is the concentration of DNA in base pairs, *ε_f_* is the extinction coefficient for the free complex at the corresponding λ_max_, *ε_A_* = A_obsd_/[compound], and *ε_b_* is the extinction coefficient for the complex in the fully bound form.

Control measurements with DMSO showed no changes in the ct-DNA spectra.

### 3.9. EB-DNA Displacement Method

Competitive fluorescence binding experiments were carried out to assess the displacement of ethidium bromide (EB) from the EB–DNA complex.

The EB-DNA adduct was prepared by adding 20 μM EB and 54 μM ct-DNA in citrate buffer (pH = 7.0). The possible intercalating effect of the compounds was studied by adding increasing concentrations of the corresponding complexes **5a–c** (c = 1 × 10^−3^ M) to a solution of the DNA-EB complex. The added complex concentration ranged from 0 to 200 μM. Fluorescence emission spectra were recorded in the range 550–800 nm with an excitation wavelength of 515 nm. The complexes showed no fluorescence emission bands in their own solution or in the presence of ct-DNA or EB. Quenching of the EB-DNA emission band by compounds **5a–c** was calculated via the Stern–Volmer equation (Equations (2)–(4)).

The extent of the inner-filter effect can be roughly estimated with the following equation:

(2) $$I_{corr}=I_{meas}\times 10^{\frac{{\varepsilon (\lambda }_{exc})cd}{2}}\times 10^{\frac{{\varepsilon (\lambda }_{em})cd}{2}}$$

where *I_corr_* = corrected intensity, *I_meas_* = the measured intensity, c = the concentration of the quencher, d = the cuvette (1 cm), and *ε_(λexc)_* and *ε_(λem)_* = the ε of the quencher at the excitation and the emission wavelengths, respectively, as calculated from the UV-Vis spectra of the complexes. The inner filter effect was evaluated with Equation (2), was found to be negligible and could not affect the measurements.

The quenching of the EB-DNA emission band by compounds was calculated via the Stern–Volmer equation:

(3) $$\frac{Io}{I}=1+k_{q}\tau_{0}\left[Q\right]=1+K_{SV}\left[Q\right]$$

where *Io* = the initial fluorescence intensity of the EB-DNA adduct, *I* is the fluorescence intensity of the EB-DNA adduct after the addition of the quencher (studied complexes), *k_q_* = the quenching rate constant, *K_SV_* = the Stern–Volmer constant, *τ_o_* = the average fluorescence lifetime of EB-DNA adduct (23 × 10^−9^ s), [Q] = the concentration of the quencher, *K_SV_* (in M^−1^) can be obtained by the slope of the Stern–Volmer plot, and *k_q_* (in M^−1^ s^−1^) may be calculated from Equation (4):

(4) $$K_{SV}=k_{q}\tau_{0}$$

### 3.10. Viscosimetric Studies

During the viscosity assay, the viscosity of the DNA solution (0.1 mM) was measured in the presence of increasing concentrations of the complexes in a buffer solution (15 mM sodium citrate and 0.15 M NaCl at pH = 7.0) at a constant temperature of 25 °C. The measurements were carried out using an ALPHA L Fungilab rotational viscometer (IKA, Staufen im Breisgau, Germany) equipped with an 18 mL ELVAS spindle at 60 rpm. The relation between the relative solution viscosity (η/η_0_) and DNA length (I/I_0_) is given by Equation (1), where η and η_0_ are the viscosities of DNA in the presence and absence of the studied complex.

Changes in the viscosity of the DNA solution (1 × 10^−4^ M) were measured in the presence of increasing concentrations of the compounds **5a–c** in a buffer solution (15 mM sodium citrate and 0.15 M NaCl at pH = 7.0) at a constant temperature of 25 °C. Measurements were carried out using an ALPHA L Fungilab rotational viscometer (IKA, Staufen im Breisgau, Germany) equipped with an 18 mL ELVAS spindle, at 60 rpm. The relation between the relative solution viscosity (η/η_0_) and DNA length (I/I_0_) is given by Equation (1), where η and η_0_ are the viscosities of DNA in the presence and absence of the studied complex.

(5) $${\left(\frac{\eta }{\eta_{0}}\right)}^{1/3}=\frac{I}{I_{0}}$$

### 3.11. Thermal Degradation Studies

Thermal denaturation of ct-DNA was monitored using a Jasco V 750 UV-Vis spectrophotometer equipped with a temperature control system. The absorbances of ct-DNA (c = 7.5 × 10^−4^ M) at 40–95 °C in the absence and presence of the complexes (c = 1 × 10^−4^ M) were recorded at 260 nm in phosphate buffer with a heating rate of 1 °C. The absorbance at 260 nm was monitored for either the ct-DNA solution (c = 7.5 × 10^−4^ M) or a mixture of ct-DNA with complexes **5a–c** in phosphate buffer at a heating rate of 1 °C. The melting temperatures (T_m_) were evaluated as the maximum of the first derivative plots of the melting curves.

### 3.12. Bovine Serum Albumin (BSA) Binding Studies

Albumin binding studies of complexes **5a–c** were determined using tryptophan fluorescence quenching assays with BSA (30 μM) in buffer solution containing 15 mM trisodium citrate and 0.15 M NaCl (pH 7.0). The quenching of the emission intensity of tryptophan residues of BSA at 336 nm was monitored using increasing concentrations of complexes **5a–c** [44]. The concentration of added complexes ranged from 0 to 2 × 10^−5^ mol mL^−1^. The fluorescence emission spectra were recorded in the range 300–420 nm with an excitation wavelength of 280 nm. The values of the Stern–Volmer constant *K_SV_* (in M^−1^), the BSA-quenching constant *k_q_* (in M^−1^ s ^−1^), and the BSA-binding constant *K* (in M^−1^) for the interaction of the compounds with BSA have been derived from the Stern–Volmer equations (Equations (2)–(4)).

The BSA binding constants *K* (in M^−1^) for the interaction of the compounds with BSA were derived from the Scatchard equation:

(6) $$\frac{\Delta I/Io}{\left[Q\right]}=nK-K\frac{\Delta I}{Io}$$

The BSA binding constant *K* (in M^−1^) can be calculated from the slope of the Scatchard plots, and the number of binding sites per albumin (*n*) is given by the ratio of the y-intercept to the slope.

In these experiments (UV-Vis and fluorescence measurements), we applied a dilution correction to the working standard solution to prevent dilution from affecting peak intensity.

### 3.13. Resazurin Cytotoxicity Assay

The assay is based on the ability of living cells to reduce blue resazurin (7-hydroxy-3*H*-phenoxazin-3-one 10-oxide) to pink resorufin (7-hydroxy-3H-phenoxazin-3-one), which can be measured by UV-Vis spectrophotometry or fluorometry (AlamarBlue^TM^,Thermo Fisher Scientific Inc., Waltham, MA, USA). Living cells have active *N*-methylphenazinium methosulfate that catalyzes the redox reaction in mitochondria of NADH and blue resazurin to produce pink resorufin (Scheme S6-1) [90,91,92,93]. This test is used as an oxidation–reduction indicator in cell viability assays for yeast and mammalian cells. Bioreduction of the dye by viable cells reduces the oxidized form (blue) and concomitantly increases the fluorescent intermediate (pink), indicating the degree of cytotoxicity caused by the test material [90,91]. The resazurin-based assay correlates well with reference viability assays such as formazan-based assays (MTT/XTT) and tritiated thymidine-based techniques [66].

*S. cerevisiae* cells (CCY 21-4-64; Libáň, Czech Republic) were cultured at 28 °C/24 h on Sabouraud’s dextrose agar with chloramphenicol (Merck, Germany). After 24 h, *S. cerevisiae* cells were inoculated into sterile liquid YPD medium (Merck, Germany), and cultivation was again carried out at 28 °C for 24 h. In the exponential growth phase, cells were centrifuged (8.000 g/3 min), washed twice with sterile physiological solution (0.9% NaCl), and pipetted at a concentration of 1 × 10^9^ cells/mL into sterile microtubes (modified procedure from Bitacura et al.) [66]. Sterile physiological solution and copper complexes at a concentration of 1 × 10^−3^ M in ratios of 0.9:0.1, 0.7:0.3, and 0.5:0.5 were added to the centrifuged biomass to a total volume of 1 mL. Thus, the final complex concentrations in the samples were 1 × 10^−4^ M, 3 × 10^−4^ M, and 5 × 10^−4^ M. The individual samples were cultured in an incubator at 28 °C for 3 h. After 3 h, 0.1 mL of Resazurin_OX_ (resazurin-based in vitro toxicology assay kit, Merck, Darmstadt, Germany) was added to each sample, and the samples were cultured for 2 h. After incubation, the biomass was separated from the supernatant by centrifugation (8.000 g/5 min). The separated solution was diluted in a 1:1 ratio with physiological solution and subjected to measurement of UV spectra by the spectrophotometric method according to the recommended protocol from the kit manufacturer (Merck, Darmstadt, Germany) on a GENESYS 10S UV-Vis spectrophotometer (Thermo Fisher Scientific Inc., Waltham, MA, USA) in the range of 500–800 nm, using a 1 cm long cuvette.

The cell’s innate metabolic rate was measured using an established formula for percentage reduction, using the appropriate molar extinction coefficients (Table 12). The percentage was calculated by the following formula:

(7) $$Red(\%)=\frac{{Ɛ}_{OX\_600\;nm}\times A_{570\;nm\_sample}-{Ɛ}_{OX\_570\;nm}\times A_{600\;nm\_sample}}{{Ɛ}_{RED\_570\;nm}\times A_{600\;nm\_C-}-{Ɛ}_{RED\_600\;nm}\times A_{570\;nm\_C-}}\times 100$$

### 3.14. MTT Cytotoxicity Assay

The cytotoxicity of the prepared copper(II) complexes in human cell lines was evaluated using the 3-(4,5-dimethylthiazol-2-yl)-2,5-diphenyltetrazolium bromide (MTT) assay following the protocol by Mosmann. Briefly, cells (A549, HepG2, and HEL) were seeded in plastic 96-well plates at a density of 1.5–2 × 10^4^ cells per well. Cells were treated for 72 h with **5a–c** and cisplatin dissolved in DMSO and incubated at 37 °C in a 5% CO_2_ atmosphere. After treatment, the samples were washed with phosphate-buffered saline (PBS), then incubated with 1 mg/mL MTT for 4 h. The MTT was then removed, and the formazan crystals were dissolved with dimethyl sulfoxide for 30 min. The photometric evaluation (at 540 nm and 690 nm) was carried out using an xMark™ Microplate Absorbance Spectrophotometer (Bio-Rad, Hercules, CA USA) [95].

#### Cell Lines

Cells A549 (ATCC^®^ CCL-185), HepG2 (ATCC^®^ HB-8065), and HEL299 (ATCC^®^ CCL-137) were obtained from Lambda Life (Bratislava, Slovakia). A549 cells were kept in Dulbecco’s Modified Eagle’s Medium (DMEM) supplemented with 10% fetal bovine serum (FBS) and 1% antibiotics (penicillin and streptomycin). HepG2 cells were kept in Williams medium supplemented with 10% fetal bovine serum (FBS) and 1% antibiotics (penicillin and streptomycin). HEL299 cells were kept in Minimum Essential Medium (MEM) supplemented with 10% fetal bovine serum (FBS), 1% antibiotics (penicillin and streptomycin), and 1% non-essential amino acids (NEAA). Cells were cultured in a humidified incubator at 37 °C with 5% CO_2_. Once the cells reached approximately 80% confluence, they were harvested and subcultured after trypsin treatment.

### 3.15. INT Radical Scavenging Assay

The method is based on 2-(4-iodophenyl)-3-(4-nitrophenyl)-5-phenyl-2H-tetrazolium chloride (INT), which can react with an electron from a radical (superoxide radical anion from KO_2_ in our case). The reaction produces a blue-green formazan, which is detected as light absorption at 500 nm. Radical scavengers (complexes **5a–c**) compete with INT for radicals and reduce the formation of formazan and light absorption at 500 nm.

Antiradical activity measurements were performed on a Synergy HT BioTek spectrophotometer (Agilent Technologies, Santa Clara, CA, USA). DMSO (analytical reagent grade) was used as a solvent, 2-(4-Iodophenyl)-3-(4-nitrophenyl)-5-phenyl-2*H*-tetrazolium chloride (INT) and KO_2_ were purchased from Alfa-Aesar (Thermo Fisher Scientific Inc., Waltham, MA, USA). The initial INT solution was prepared in a sodium tetraborate buffer solution with pH adjusted to 7.1 using HCl [29,39,44].

For each measurement, an initial solution of the sample at a specific concentration was prepared (4 × 10^−3^ M and 1 × 10^−4^ M) in DMSO, as well as initial solutions of INT (4 M) in tetraborate buffer (pH = 7.1) and a saturated solution of KO_2_ in DMSO. After adding INT and KO_2_ solutions and additional DMSO, the concentrations of the measured complexes decreased sevenfold (0.5 mL of initial sample solution in 3.5 mL of overall mixture). Each measurement was averaged across the 3 sample solutions, with standard deviation (more information in Supplementary Materials). For the determination of SOD-IC_50_, a series of measurements with decreasing sample concentration were evaluated. As the negative logarithm of complex concentration decreased, the % inhibition also decreased in a linear trend. The linear trend equation was used to determine SOD-IC_50_ values as the concentration causing 50% inhibition of INT-formazan formation, indicating the complexes’ radical scavenging efficiency.

## 4. Conclusions

Reduced Schiff base ligands (**4a–c**) derived from salicylaldehyde and 2-, 3-, and 4-methoxybenzylamines were prepared and used to prepare three copper(II) complexes (**5a**: 2-methoxyderivative, **5b**: 3-methoxyderivative, **5c**: 4-methoxyderivative). X-ray structure analysis determined the binuclear O,N-bidentate structure of the complexes, revealing a common binuclear arrangement in all three complexes, with acetate ligands binding either by both oxygens to one copper and/or to two neighboring copper centers forming a polymer (in complex **5a**).

The prepared complexes were used in DNA binding studies by absorption titration and EB-DNA displacement methods. The DNA binding studies showed similar binding constants (*K_b_* =1.25–3.90 × 10^4^ M^−1^), which are lower than for EB (1.23 × 10^5^ M^−1^), and similar Stern–Volmer quenching constants in the EB displacement method (*K_SV_* = 3.4–4.3 × 10^3^ M^−1^) and fluorescence quenching up to 38–43%. Complex **5c** showed better *K_b_* and EB displacing properties than **5b**, while both have very similar DNA binding constants, higher than in the case of **5a**. The mode of binding for all complexes was determined by viscosity and DNA degradation studies. The results show that groove binding is the most likely binding mode.

The DNA cleavage (pDNA model) studies showed that complex **5a** performed best, with 94.5% one-strand cleavage to form II, followed by **5c** (64.3%) and **5b** (37.2%) at 5 × 10^−3^ M. However, at lower concentrations, the activities of all three complexes were mostly around 24–36%.

The cytotoxicity of the complexes was studied using a cell survival model monitoring mitochondrial activity in *S. cerevisiae* (Resazurin model) and the MTT assay on cancer cell lines (HepG2 and A549) versus HEL fibroblast cells. All three complexes showed very high antimitochondrial activity (around 95% cell death at 1 × 10^−4^ M), with no major differences among the complexes. The MTT assay uncovered a similar activity profile for all three complexes. All three complexes showed impressive selectivity towards HepG2 cells, with selectivity indices (HEL/HepG2) of 3.05 for **5a**, 2.48 for **5b**, and 2.08 for **5c**. Complex **5b** showed the lowest IC_50_ against HepG2 at 33.0 ± 5.3 μM, followed by the other complexes with IC_50_ around 42–45 μM. Compared with cisplatin, the IC_50_ against HepG2 (16.0 ± 3.4 μM) is close to that of **5b**. The IC_50_ of cisplatin against noncancerous fibroblasts HEL is much lower than for the complexes **5a–c** at 68.4 ± 0.3 μM, but the selectivity index (HEL/HepG2) for cisplatin is 4.28. The IC_50_ values against A549 cells were close to those against HEL cells.

The position of the methoxy group on the ligands influenced the activities of the copper(II) complexes. Complex **5b** with *meta* substitution showed the highest binding constants for ct-DNA, was the most cytotoxic of the prepared complexes in the MTT assay, and was the best superoxide scavenger with an SOD-IC_50_ of 152 ± 5 µM. Complex **5c**, with a para-methoxy group, displaced EB from DNA the best, and *ortho*-methoxy-derived complex **5a** showed significantly higher DNA cleavage.

Our study showed the usefulness of copper complexes of the reduced Schiff base ligands. They showed interesting DNA binding, DNA cleavage, cytotoxic, and SOD mimetic properties. The selectivity indices show promise for targeting HepG2 colon cancer cells over noncancerous HEL cells. The possible reversible binding to BSA showed promise for delivery of the complexes in the organism, but pharmacokinetics of the complexes require additional research.

## Figures and Tables

**Scheme 1:**
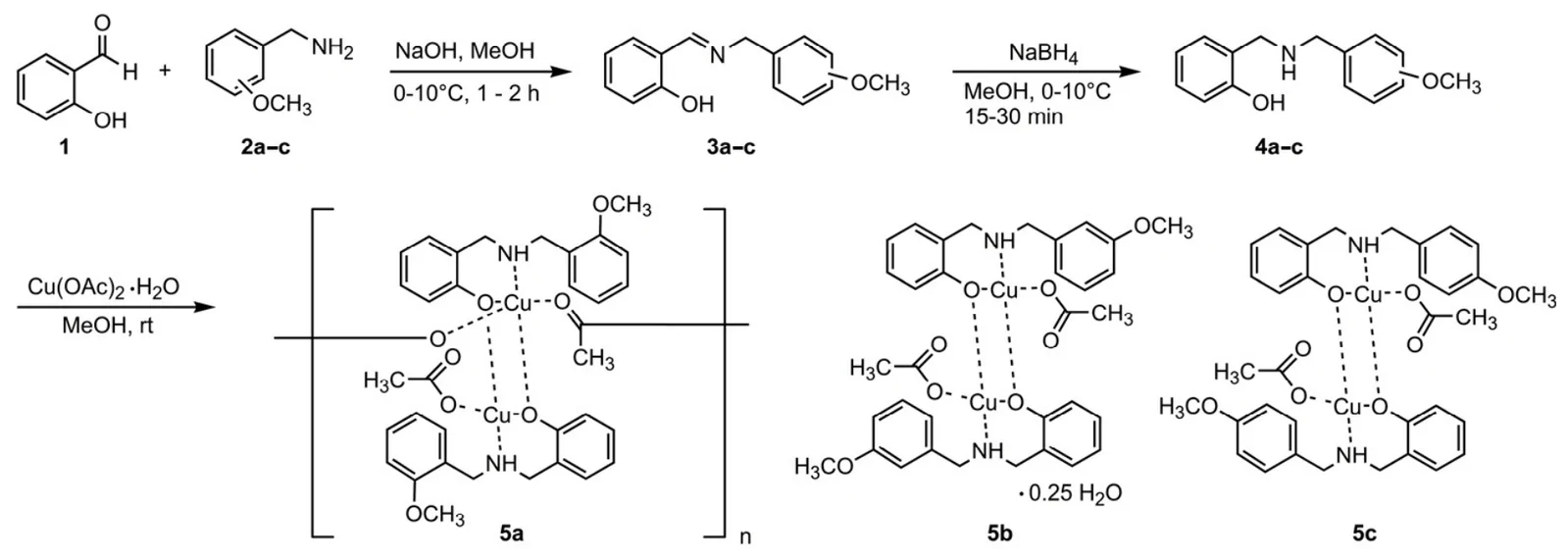
Overall synthesis of reduced Schiff bases **4a–c** and the copper(II) complexes **5a–c** (**a**: 2-methoxy-, **b**: 3-methoxy-, **c**: 4-methoxyderivative). The dashed bonds mark coordination bonds.

**Figure 1:**
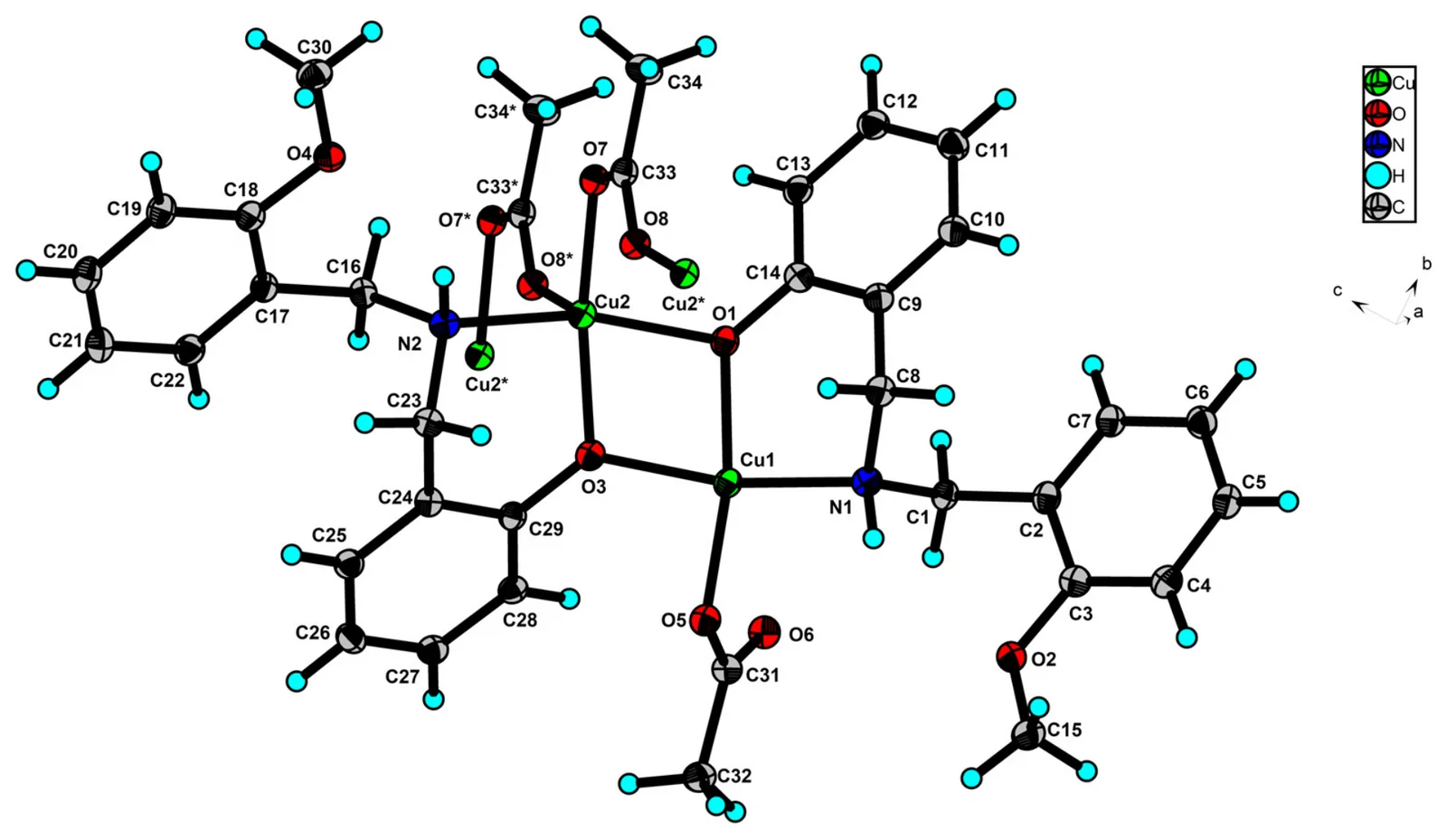
Molecular structure of complex **5a** (with polymer detail) showing the atom labeling scheme without hydrogen atom labels for clarity. Displacement ellipsoids are drawn at the 50% probability level. * marks the polymer structure.

**Figure 2:**
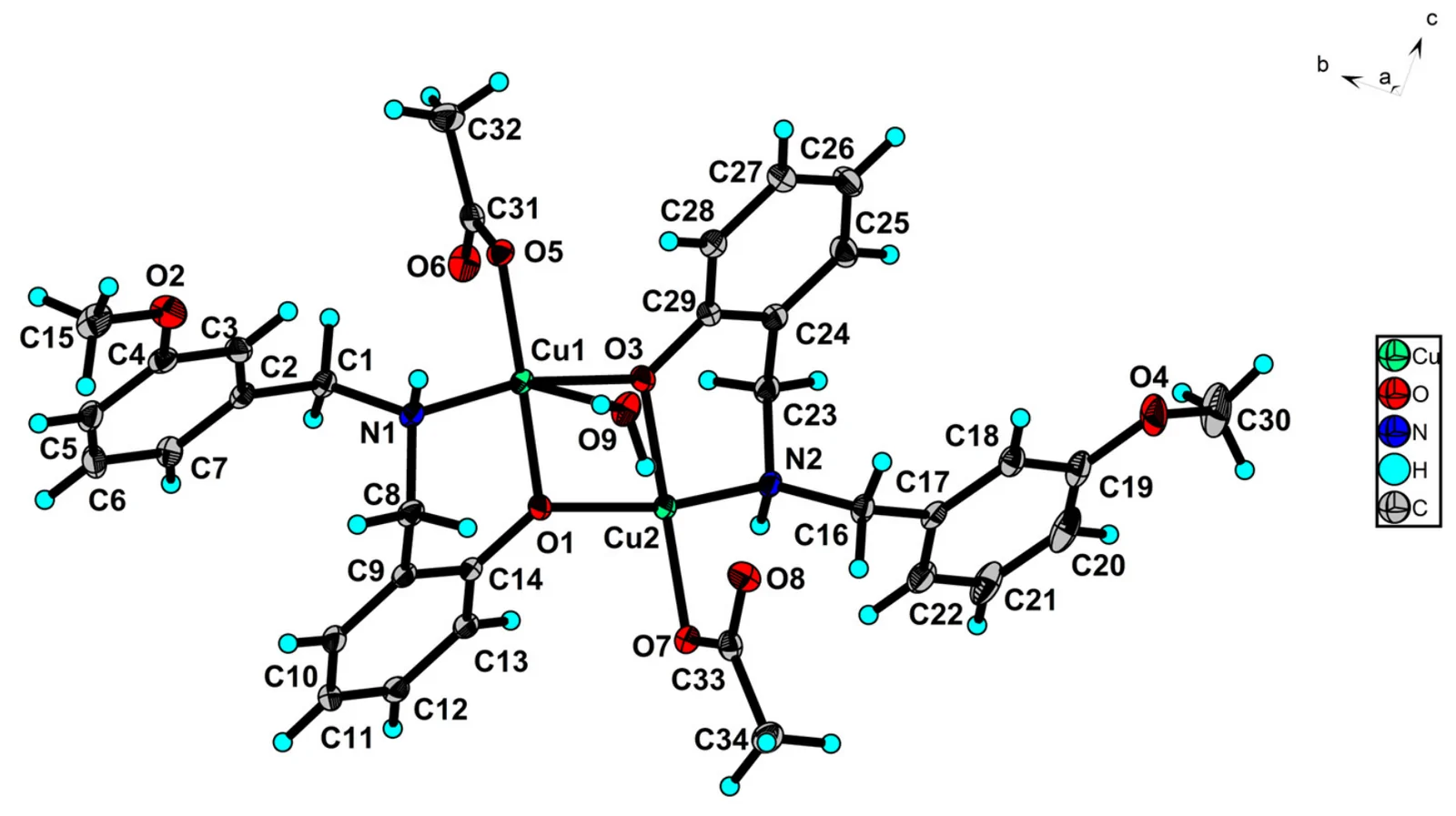
The molecular structure (as a binuclear complex) of complex **5b** showing the atom labeling scheme without hydrogen atom labels for clarity. Displacement ellipsoids are drawn at the 50% probability level.

**Figure 3:**
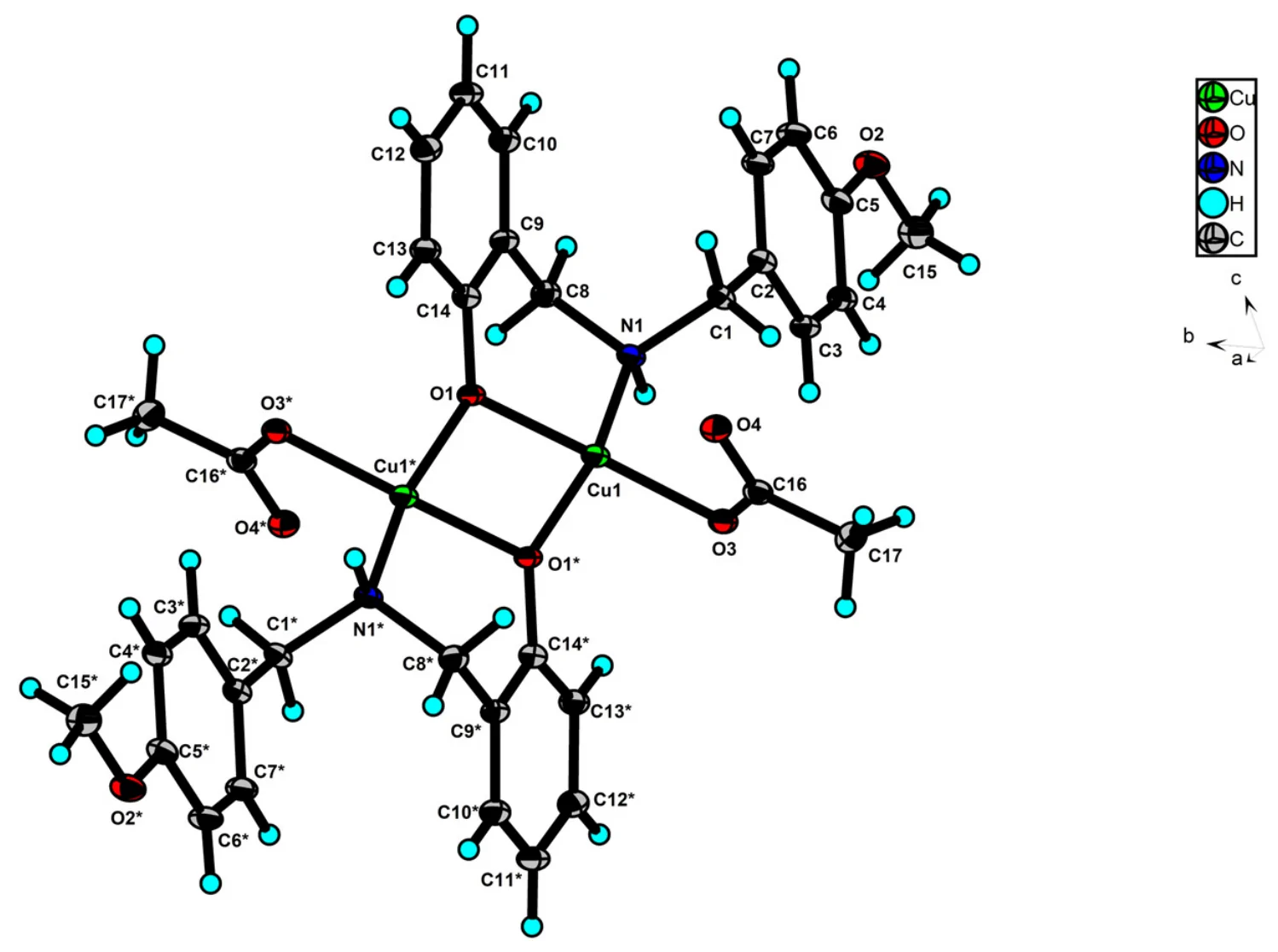
The molecular structure of complex **5c**—a symmetric binuclear complex—showing the atom-labeling scheme without hydrogen atom labels for clarity. Displacement ellipsoids are drawn at the 50% probability level. * marks the asymmetric structure.

**Figure 4:**
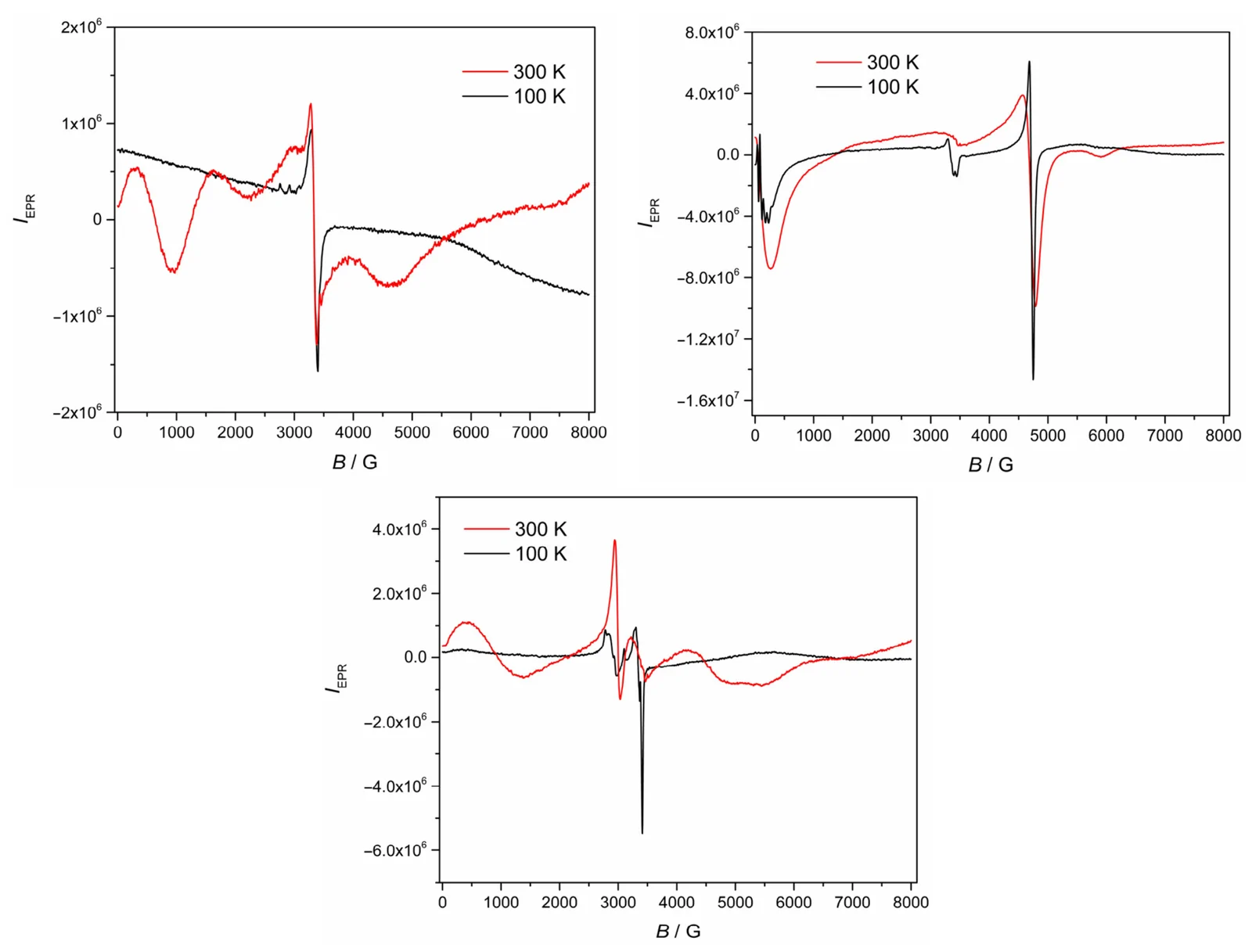
EPR spectra of complexes **5a** (upper left), **5b** (upper right), and **5c** (below) recorded in perpendicular mode at 100K and 300K.

**Figure 5:**
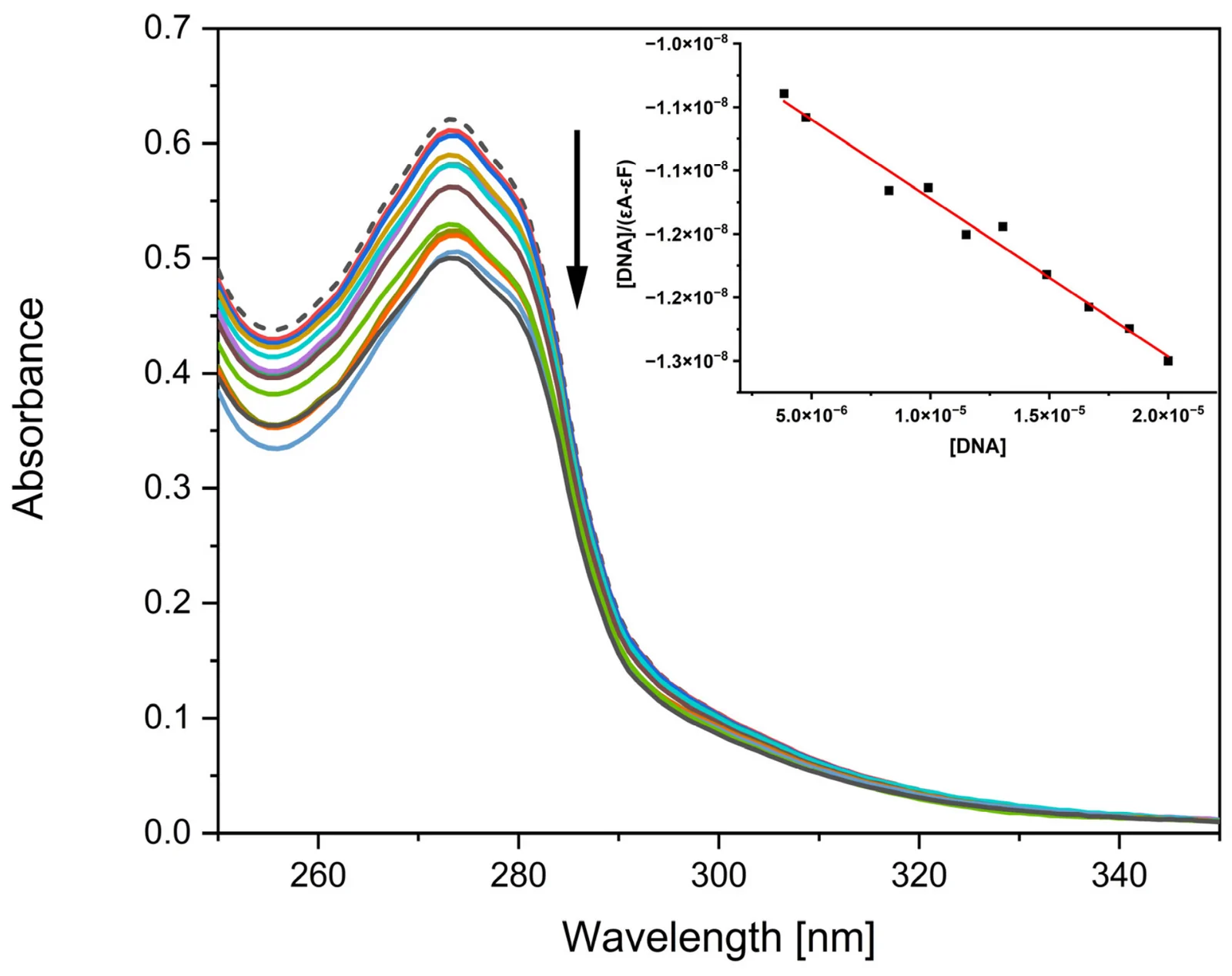
UV-Vis spectra of complex **5a** in the absence and presence of increasing amounts of ct-DNA (*r* = [DNA]/[complex] = 0–2.1). The arrows show intensity changes as increasing amounts of ct-DNA are added (every concentration increment of 2 × 10^−6^ M decreased the absorbance, which is indicated by different curve colors). Insert graph: Plots [DNA]/(ε_A_-ε_f_) *versus* [DNA] according to the Wolfe–Shimer equation.

**Figure 6:**
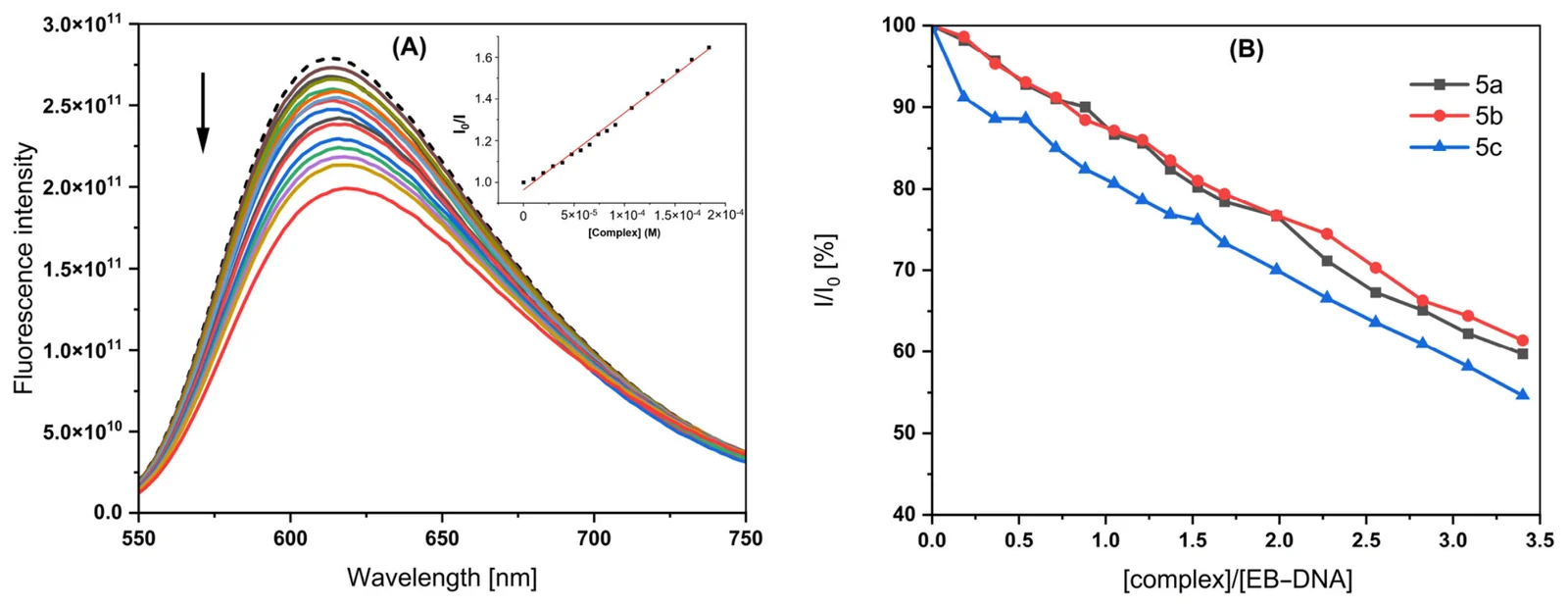
(**A**) Emission fluorescence spectra of EB-DNA in the buffer solution in the presence of increasing amounts of complex **5a**. The concentration of the added complex ranged from 0 to 2 × 10^−4^ M (each concentration increment of 1.2 × 10−5 M decreased the fluorescence intensity, as indicated by the different colors of the curves). The arrow indicates the changes in fluorescence with increasing amounts of the complex. The insert graph shows the plot of I_0_/I vs. [complex]. (**B**) Plot of EB-DNA relative fluorescence intensity (I/I_0_, % at λ = 614 nm vs. concentration ratio complex/EB-DNA for **5a–c**.

**Figure 7:**
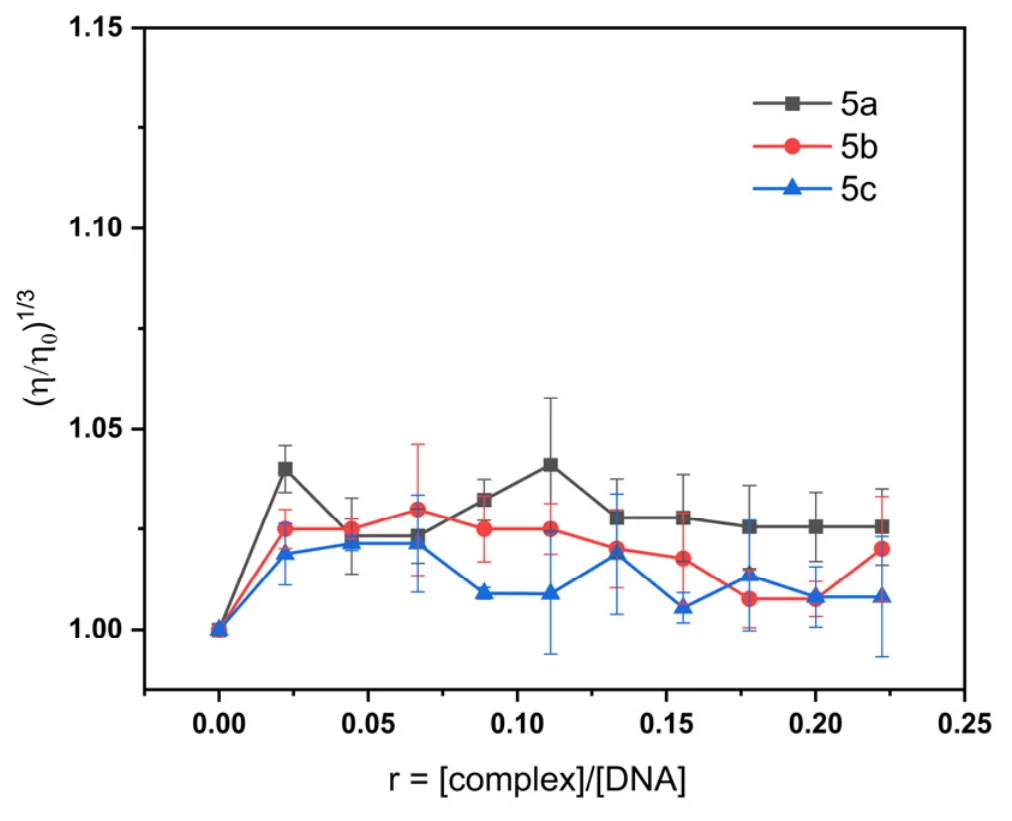
Viscosity measurements of ct-DNA in the buffer solution in the presence of studied complexes **5a–c**, under the condition of increasing the concentration ratio [complex]/[DNA]; [DNA] = 100 µM, [complex] = 0–22 µM.

**Figure 8:**
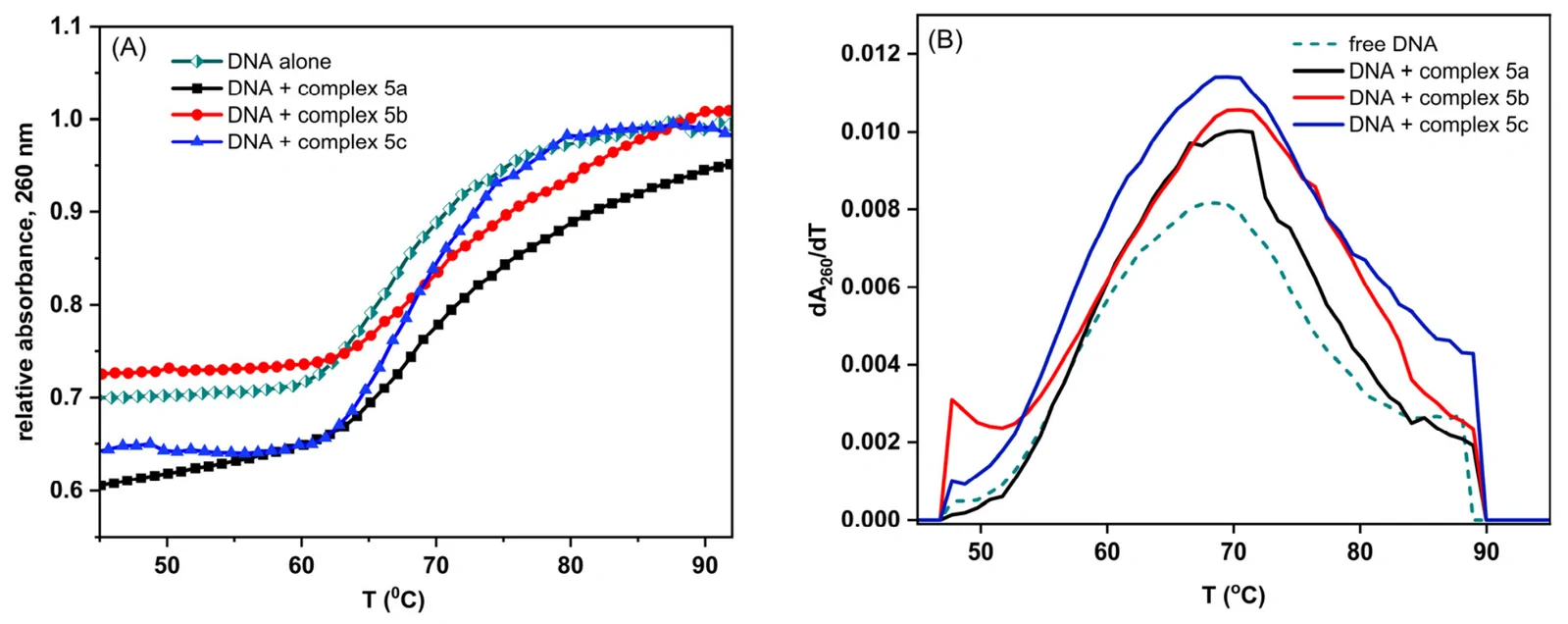
(**A**) Melting curves of free ct-DNA (c = 7.5 × 10^−4^ M) and ct-DNA with complexes **5a–c** (c = 1 × 10^−4^ M). (**B**) The first derivatives of melting curves of ct-DNA in the presence of complexes. (Tm = 68.5 °C for free DNA, Tm = 71.1 °C, 69.5 °C, and 70.7 °C for **5a**, **5b**, and **5c**, respectively).

**Figure 9:**
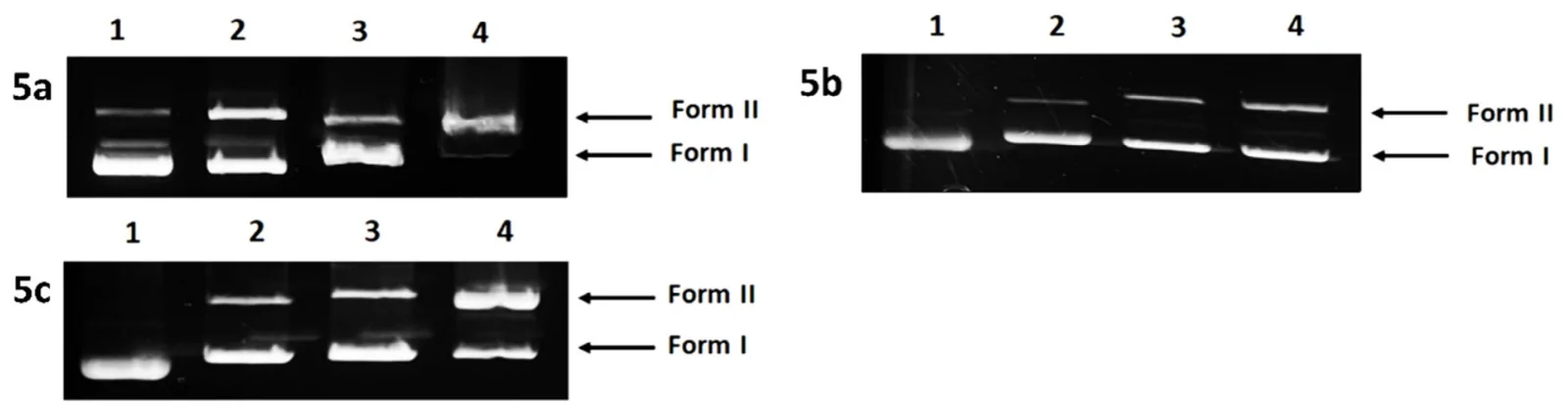
Agarose gel electrophoresis patterns of the cleavage of the pBR322 DNA plasmid by the copper complexes. Line 1—Control (undigested DNA plasmid), Line 2—DNA plasmid digested with copper complex at a concentration of 1 × 10^−3^ M, Line 3—DNA plasmid digested with copper complex at a concentration of 3 × 10^−3^ M, Line 4—DNA plasmid digested with copper complex at a concentration of 5 × 10^−3^ M. Form I—supercoiled plasmid DNA (undigested plasmid), Form II—open-circular plasmid DNA (partly digested plasmid).

**Figure 10:**
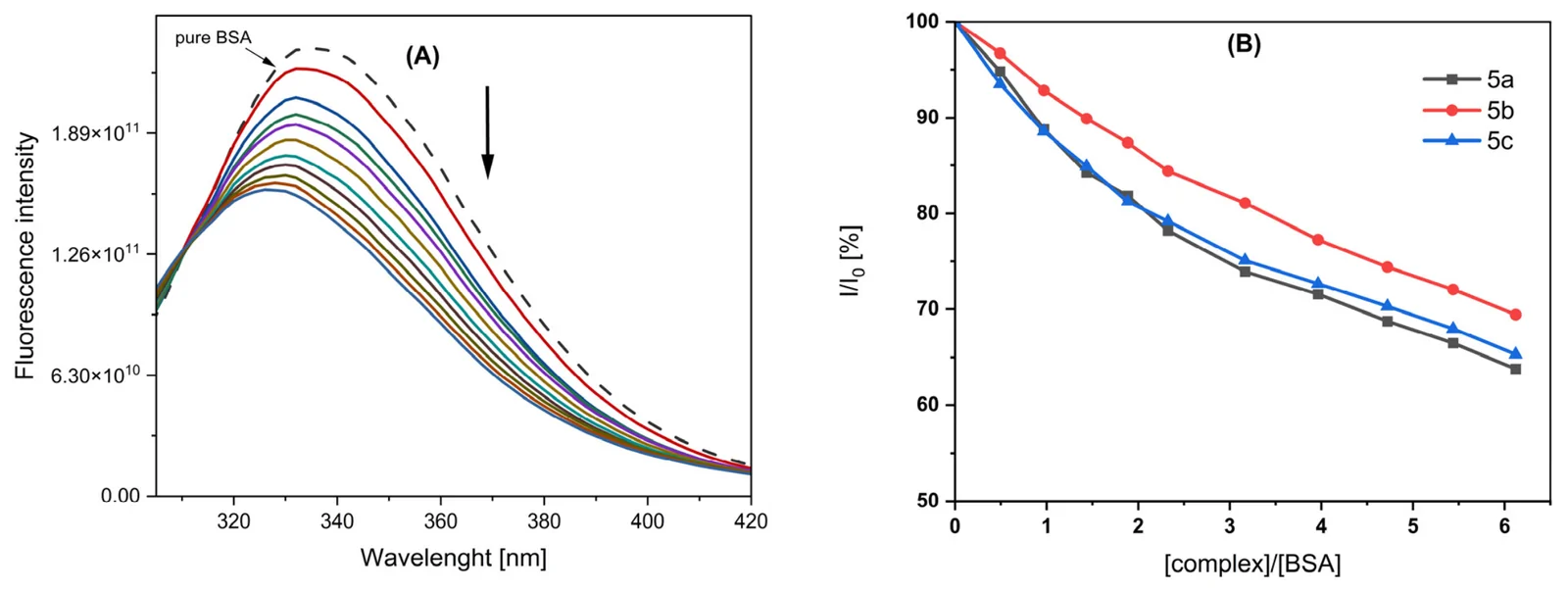
(**A**) Representative fluorescence emission spectra of BSA in buffer solution in the presence of increasing concentrations of complex **5a**. The arrow indicates changes in fluorescence as complex concentration increases. The concentration of added complexes ranged from 0 to 1.6 × 10^−5^ M (concentration increment of 1.6 × 10^−6^ M indicated by different colors of the curves). (**B**) Graphical dependence of relative BSA fluorescence intensity in % at *λ* = 336 nm vs. concentration ratio [complex]/BSA for complexes **5a–c**.

**Figure 11:**
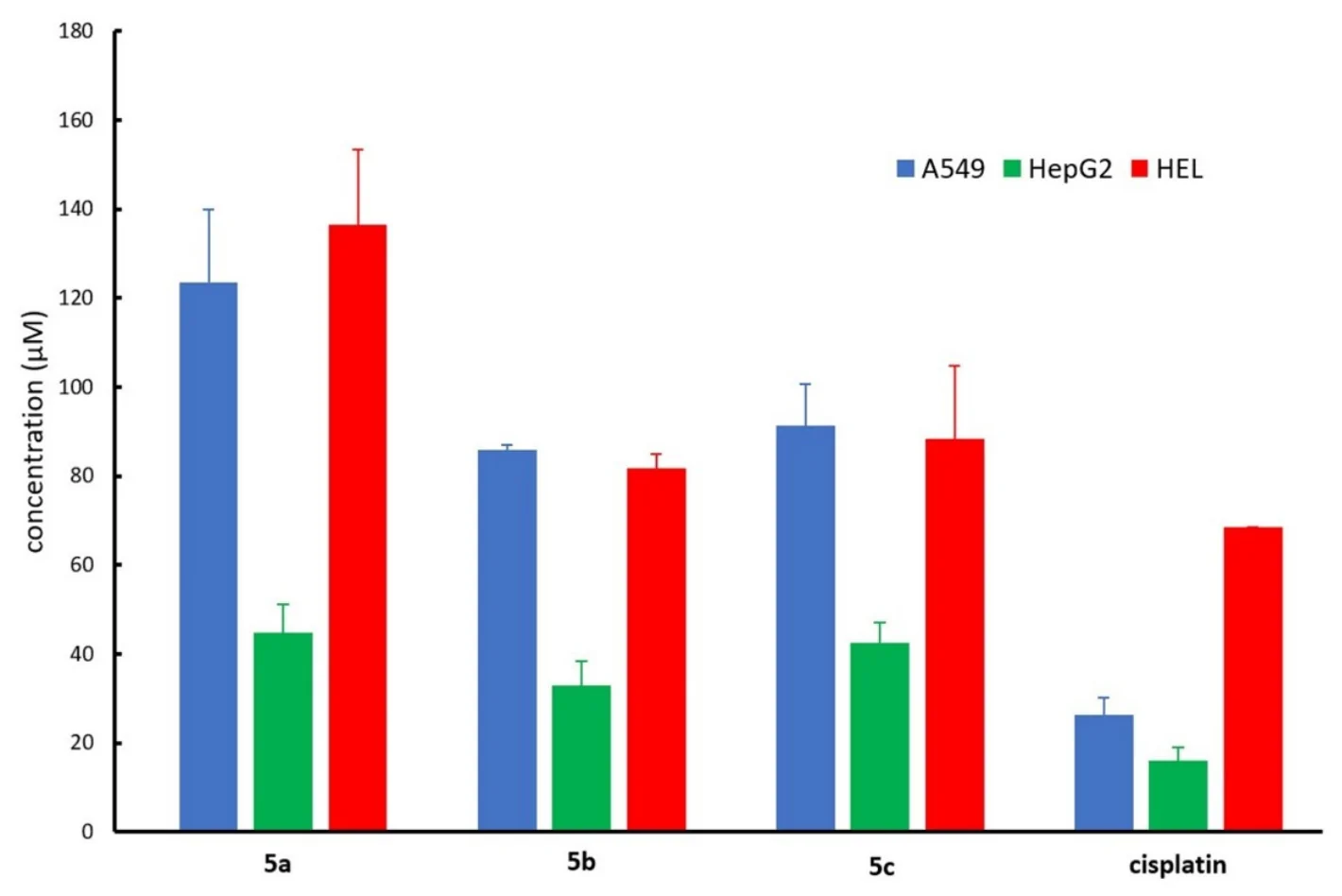
IC_50_ concentrations for cytotoxicity against cancer cells A549 and HepG2 and noncancerous HEL cells.

**Table 1:** Crystal data and structure refinement for complexes **5a–c**.

Compound	5a	5b	5c
Empirical formula	C_34_H_38_ Cu_2_N_2_O_8_	C_34_H_38.5_Cu_2_N_2_O_8.25_	C_34_H_38_Cu_2_N_2_O_8_
Temperature [K]	150 (1)	100 (1)	150 (1)
Wavelength [Å]	1.54186	1.54186	1.54186
Crystal system	Monoclinic	Monoclinic	Triclinic
Space group	*P*n	*P*2_1_/n	*P*-1
Unit cell dimensions [Å], [°]	a = 4.9823 (2) b = 18.9202 (8) c = 17.0362 (6) β = 90.673 (3)	a = 10.9914 (1) b = 18.4756 (2) c = 16.1150 (2) β = 92.028 (1)	a = 5.1854 (4) b = 11.4767 (6) c = 14.7309 (7) α = 69.597 (5) β = 81.266 (5) γ = 81.950 (6)
Formula weight	729.74	734.25	729.74
Volume [Å^3^]	1605.83 (11)	3270.47 (6)	808.53 (9)
Z/Calculated density [Mg/m^3^]	2/1.509	4/ 1.491	1/1.499
Absorption coeff. [mm^−1^]	2.092	2.060	2.078
F(000)	756.0	1522.0	378.0
Crystal size [mm]	0.110 × 0.050 × 0.020	0.220 × 0.107 × 0.045	0.150 × 0.063 × 0.020
Theta range for data collection	4.67 to 144.58°	7.282 to 144.5°	6.442 to 133.136°
Index ranges	−6 ≤ h ≤ 4 −21 ≤ k ≤ 23 −17 ≤ l ≤ 21	−13 ≤ h ≤ 6 −22 ≤ k ≤ 22 −19 ≤ l ≤ 19	−6 ≤ h ≤ 4 −13 ≤ k ≤ 13 −17 ≤ l ≤ 16
Reflections collected/Independent reflections	47,861/4890 [R_(int)_ = 0.0922, R_σ_ = 0.0452]	101,713/6339 [R_(int)_ = 0.0314, R_σ_ = 0.0137]	30,945/2841 [R_(int)_ = 0.1128, R_σ_ = 0.0543]
Refinement method	Full-matrix least-squares on F2		
Data/restraints/parameters	4890/2/419	6339/0/429	2841/0/210
Goodness-of-fit on F^2^	1.069	1.084	1.055
Final R indices [I > 2σ(I)]	R_1_ = 0.0566, wR_2_ = 0.1364	R_1_ = 0.0328 wR_2_ = 0.0923	R_1_ = 0.0595 wR_2_ = 0.1379
R indices (all data)	R_1_ = 0.0777 wR_2_ = 0.1552	R_1_ = 0.0375 wR_2_ = 0.0953	R_1_ = 0.0917 wR_2_ = 0.1642
Largest diff. peak and hole [e.Å^−3^]	0.74/−0.30	0.42/−0.76	0.63/−0.95
Flack parameter	−0.05 (4)	-	-

**Table 2:** Selected bond lengths and angles for complex **5a**.

Atom	Atom	Length/Å	Position	Atom	Atom	Length/Å	Position
Cu1	O5	1.932 (7)	eq.	Cu2	O7	1.941 (6)	eq.
Cu1	O3	1.953 (7)	eq.	Cu2	O3	1.943 (7)	eq.
Cu1	O1	1.952 (7)	eq.	Cu2	O1	1.989 (7)	eq.
Cu1	N1	1.996 (8)	eq.	Cu2	N2	2.038 (8)	eq.
Cu1	Cu2	3.0421 (17)	between	Cu2	O8 *	2.291 (6)	ax.
Atom	Atom	Atom	Angle/°	Atom	Atom	Atom	Angle/°
O5	Cu1	Cu2	129.3 (2)	O3	Cu1	N1	166.7 (3)
O5	Cu1	O3	94.2 (3)	O1	Cu1	Cu2	39.9 (2)
O5	Cu1	O1	169.1 (3)	O1	Cu1	O3	76.1 (3)
O5	Cu1	N1	95.7 (3)	O1	Cu1	N1	93.2 (3)
O3	Cu1	Cu2	38.6 (2)	N1	Cu1	Cu2	128.8 (2)
O8 *	Cu2	Cu1	111.08 (17)	O7	Cu2	N2	91.0 (3)
O7	Cu2	Cu1	130.2 (2)	O3	Cu2	Cu1	38.8 (2)
O7	Cu2	O8 *	99.3 (2)	O3	Cu2	O8 *	93.3 (3)
O7	Cu2	O3	166.5 (3)	O3	Cu2	O1	75.5 (3)
O7	Cu2	O1	96.7 (3)	O3	Cu2	N2	92.5 (3)
O1	Cu2	Cu1	39.04 (19)	N2	Cu2	Cu1	122.7 (2)
O1	Cu2	O8 *	104.0 (3)	N2	Cu2	O8 *	95.6 (3)
O1	Cu2	N2	157.4 (3)	Cu2	O3	Cu1	102.7 (3)
				Cu1	O1	Cu2	101.0 (3)

Symmetry transformations used to generate equivalent atoms: (*) 1 − x, 1 − y, −z.

**Table 3:** Selected bond lengths and angles for complex **5b**.

Atom	Atom	Length/Å	Position	Atom	Atom	Length/Å	Position
Cu1	Cu2	3.0399 (4)	ax.	Cu1	O9	2.262 (5)	between
Cu1	O5	1.9330 (13)	eq.	Cu2	O1	1.9428 (13)	eq.
Cu1	O1	1.9357 (13)	eq.	Cu2	O7	1.9593 (13)	eq.
Cu1	O3	1.9549 (14)	eq.	Cu2	O3	1.9401 (14)	eq.
Cu1	N1	1.9989 (15)	eq.	Cu2	N2	1.9827 (15)	eq.
Atom	Atom	Atom	Angle/°	Atom	Atom	Atom	Angle/°
O5	Cu1	O1	172.99 (6)	O1	Cu1	O3	76.73 (6)
O5	Cu1	O3	101.59 (6)	O1	Cu1	O9	87.36 (15)
O5	Cu1	O9	98.70 (15)	O3	Cu1	O9	72.96 (15)
O5	Cu1	N1	90.44 (6)	N1	Cu1	Cu2	128.93 (4)
O1	Cu1	N1	92.46 (6)	O1	Cu1	Cu2	38.48 (4)
N1	Cu1	O9	95.78 (15)	O5	Cu1	Cu2	140.10 (4)
O3	Cu1	N1	164.51 (7)	O3	Cu1	Cu2	38.52 (4)
O9	Cu1	Cu2	74.06 (14)	O1	Cu2	Cu1	38.31 (4)
O1	Cu2	O7	97.86 (5)	O1	Cu2	N2	159.39 (6)
O3	Cu2	O1	76.91 (6)	O7	Cu2	Cu1	133.27 (4)
O3	Cu2	O7	165.31 (7)	O7	Cu2	N2	93.95 (6)
O3	Cu2	N2	95.30 (6)	O3	Cu2	Cu1	38.87 (4)
N2	Cu2	Cu1	132.77 (5)	Cu1	O1	Cu2	103.21 (6)
Cu2	O3	Cu1	102.60 (6)				

**Table 4:** Selected bond lengths and angles for complex **5c**.

Atom	Atom	Length/Å	Position	Atom	Atom	Length/Å	Position
Cu1	Cu1 *	3.042 (2)	between	Cu1	O3	1.969 (4)	between
Cu1	O1	1.944 (3)	eq.	Cu1	N1	1.989 (4)	eq.
Cu1	O1 *	1.954 (3)	eq.	Cu1	C16	2.541 (6)	eq.
Atom	Atom	Atom	Angle/°	Atom	Atom	Atom	Angle/°
O1 *	Cu1	Cu1 *	38.60 (10)	O1	Cu1	Cu1 *	38.84 (10)
O1	Cu1	O1 *	77.44 (15)	O1	Cu1	N1	93.64 (16)
O1 *	Cu1	O3	95.65 (14)	O3	Cu1	N1	95.21 (16)
Cu1	O1	Cu1 *	102.56 (15)	N1	Cu1	Cu1 *	131.11 (13)
O3	Cu1	Cu1 *	133.56 (11)	O1 *	Cu1	N1	163.78 (17)
O1	Cu1	O3	168.26 (16)				

Symmetry transformations used to generate equivalent atoms: (*) −x, 2 − y, 1 − z.

**Table 5:** UV-Vis spectral data of the interaction with ct-DNA, calculated DNA-binding constants (K*_b_*), UV band (λ_max_, nm), decrease in intensity (ΔA/A_0_) in %.

Complex	K*_b_* [M^−1^]	λ (nm)	(ΔA/A_0_, %)
**5a**	1.25 (±0.02) × 10^4^	273	23
**5b**	3.90 (±0.10) × 10^4^	273	18
**5c**	3.84 (±0.19) × 10^4^	273	24

**Table 6:** EB-DNA fluorescence (%), calculated Stern–Volmer quenching constant (*K_SV_*), and quenching rate constant *k_q_* of complexes **5a–c**.

Complex	∆ I/I_0_ [%]	*K_SV_* [M^−1^] × 10^3^	*k_q_* [M^−1^ s^−1^] × 10^11^
**5a**	40.3	3.68 (±0.07)	1.60 (±0.07)
**5b**	38.1	3.38 (±0.09)	1.47 (±0.06)
**5c**	43.4	4.34 (±0.12)	1.89 (±0.05)

**Table 7:** Quantitative evaluation of the nuclease activity of copper(II) complexes.

Complex	c (M)	Form I (%)	Form II (%)
**5a**	0	89.8	10.2
	1 × 10^−3^	63.9	36.1
	3 × 10^−3^	75.6	24.4
	5 × 10^−3^	5.5	94.5
**5b**	0	100	0
	1 × 10^−3^	86.1	13.9
	3 × 10^−3^	70.2	30.8
	5 × 10^−3^	62.8	37.2
**5c**	0	100	0
	1 × 10^−3^	73.6	26.4
	3 × 10^−3^	69.8	30.2
	5 × 10^−3^	35.7	64.3

**Table 8:** Values of the dynamic quenching constant (*K_SV_*), quenching constant (*k*_q_), association binding constant (*K_BSA_*), and *n* (number of binding sites for albumin) obtained for the interaction of **5a–c** with bovine serum albumin.

Complex	*K_SV_* (M^−1^)	*k_q_* (M^−1^s^−1^)	*K_BSA_* (M^−1^)	*n*
**5a**	2.98 (±0.12) × 10^4^	2.98 (±0.12) × 10^12^	8.08 (±0.41) × 10^4^	0.60
**5b**	2.37 (±0.30) × 10^4^	2.37 (±0.30) × 10^12^	3.34 (±0.12) × 10^4^	0.87
**5c**	2.72 (±0.12) × 10^4^	2.72 (±0.12) × 10^12^	8.55 (±0.24) × 10^4^	0.85

**Table 9:** Absorbance values at 570 nm and 600 nm, along with the calculated reduction percentage.

Sample	c (M)	Absorbance * 570 nm	Absorbance * 600 nm	Reduction Percentage (%)	Cell Death (%)
Positive control C+		1.10	0.12	88.6	12.4
Negative control C−		0.69	0.93	4.4	95.6
	1 × 10^−4^	0.62	0.83	4.3	95.7
**5a**	3 × 10^−4^	0.61	0.84	2.8	97.2
	5 × 10^−4^	0.60	0.84	2.0	98.0
	1 × 10^−4^	0.55	0.72	4.8	95.2
**5b**	3 × 10^−4^	0.49	0.59	7.3	92.7
	5 × 10^−4^	0.49	0.60	6.8	93.2
	1 × 10^−4^	0.65	0.85	5.7	94.3
**5c**	3 × 10^−4^	0.64	0.86	4.2	95.8
	5 × 10^−4^	0.62	0.86	2.5	97.5

* average of 3 measurements.

**Table 10:** MTT test IC_50_ values in μM ranges.

Compound	IC_50_—A549	IC_50_—HepG2	IC_50_—HEL
**5a**	123.4 ± 16.5	44.7 ± 6.4	136.5 ± 16.8
**5b**	85.8 ± 1.1	33.0 ± 5.3	81.7 ± 3.3
**5c**	91.4 ± 9.2	42.4 ± 4.7	88.4 ± 16.3
cisplatin	26.2 ± 4.1	16.0 ± 3.4	68.4 ± 0.3

**Table 11:** SOD mimetic activity expressed as % of inhibition of radical transfer to INT at concentrations c_1_ = 5.72 × 10^−4^ M and c_2_ = 1.43 × 10^−4^ M and SOD-IC_50_ values.

Compound	Activity at c_1_ (%)	Activity at c_2_ (%)	SOD-IC_50_ (μM)
**5a**	74.55 ± 0.60	12.67 ± 1.98	297 ± 14
**5b**	95.91 ± 0.58	24.01 ± 3.69	152 ± 5
**5c**	80.80 ± 0.22	16.72 ± 5.52	187 ± 4
cystamine	22.10 ± 1.77	2.01 ± 0.03	1700 ± 100

**Table 12:** Molar extinction coefficients for reduced (*Ɛ*_RED_) and oxidized Resazurin (*Ɛ*_OX_) [94].

Wavelength (nm)	*Ɛ*_RED_	*Ɛ*_OX_
570	155,677	80,586
600	14,652	117,216

## Data Availability

The original contributions presented in this study are included in the article/Supplementary Materials. Further inquiries can be directed to the corresponding authors.

## Supplementary Materials

The following supporting information can be downloaded at: https://www.mdpi.com/article/10.3390/ijms27198650/s1.

## Abbreviations

The following abbreviations are used in this manuscript:

A549	adenocarcinoma human alveolar basal epithelial cell line
BSA	bovine serum albumin
ct-DNA	calf thymus DNA
DMSO	dimethyl sulfoxide
EB	ethidium bromide
HepG2	human liver cancer cell line
HEL	fibroblast cell line, non-cancerous
INT	2-(4-Iodophenyl)-3-(4-nitrophenyl)-5-phenyl-2H-tetrazolium chloride
NADH	nicotinamide adenine dinucleotide
MTT	3-[4,5-dimethylthiazol-2-yl]-2,5 diphenyl tetrazolium bromide
pDNA	plasmid DNA
ROS	reactive oxygen species
SOD	superoxide dismutase
ZFS	zero field splitting
