# Supplementary material for: Reduced Schiff Base Copper(II) Complexes Derived from 2-, 3-, and 4-Methoxybenzylamine: DNA Binding and Cleavage, BSA Binding, Cytotoxic and SOD Mimetic Properties
Source: Int J Mol Sci. 2026 Sep 27;27(19):8650. doi: 10.3390/ijms27198650 (PMC13665063; doi:10.3390/ijms27198650)
Supplement: Supplementary file 1 [file ijms-27-08650-s001.zip › ijms-4497073-supplementary.pdf]

Reduced Schiff base Copper(II) complexes derived from 2-, 3-, and 4-methoxybenzylamine: DNA binding and cleavage, BSA binding, cytotoxic and SOD mimetic properties

Supplementary Material

Lucia Lintnerová[a,*], Peter Herich[a,b], Jana Korec[a], Barbora Svitková[c], Flóra Jozefíková[a,d], Karol Lušpai[b], Jindra Valentová[a]

[a]Department of Chemical Theory of Drugs, Faculty of Pharmacy, Comenius University in Bratislava, Odbojárov 10, 832 32, Bratislava, Slovakia

[b]Department of Physical Chemistry, Faculty of Chemical and Food Technology, Slovak University of Technology, Radlinského 9, 812 37, Bratislava, Slovakia

[c]Cancer Research Institute, Biomedical Research Center, Slovak Academy of Sciences, Dúbravská cesta 9, 845 05 Bratislava, Slovakia

[d]Department of Inorganic Chemistry, Faculty of Chemical and Food Technology, Slovak University of Technology, Radlinského 9, 812 37, Bratislava, Slovakia

* Correspondence: lintnerova@fpharm.uniba.sk (L.L.); valentova@fpharm.uniba.sk (J.V.)

S1. Spectral data

S1.1. NMR spectra comparison of Schiff base and reduced Schiff base

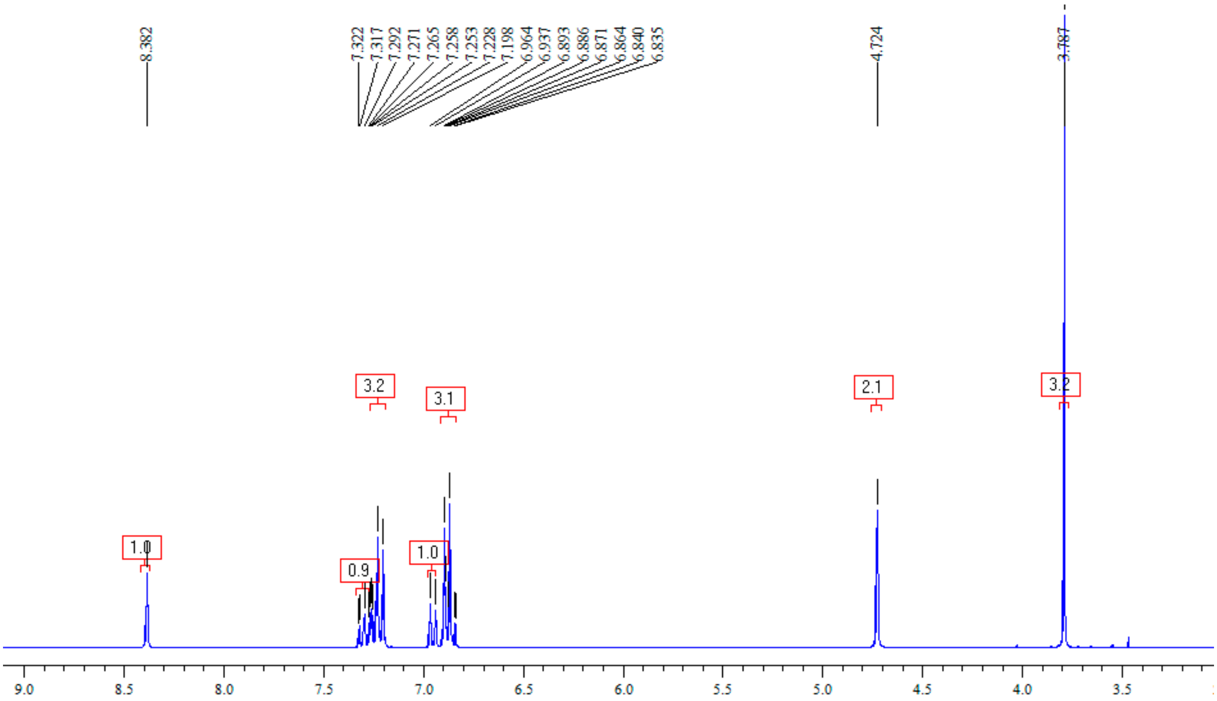

Figure S1-1: ^{1}H NMR spectrum of Schiff base **3c** with hydrogen from CH=N located at 8.382 ppm

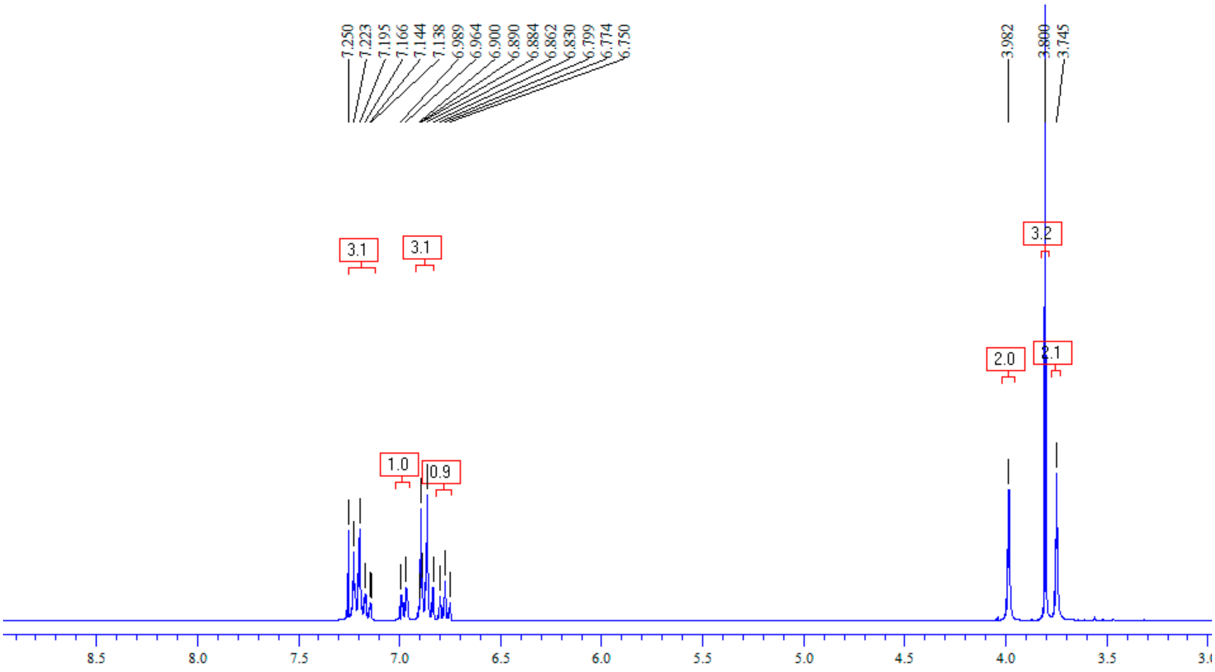

Figure S1-2: ^{1}H NMR spectrum of reduced Schiff base **4c,** which no longer contains the Schiff base signals but has a new peak in the aliphatic part of the spectrum showing two methylenes next to nitrogen (CH_2-N-CH_2) located at 3.982 and 3.745 ppm

S1.2. Solution stability studies

Stability studies were performed by UV-Vis spectroscopy over the 200 – 800 nm wavelength range in citrate buffer solution and DMSO.

A citrate buffer solution (containing 15 mM sodium citrate and 150 mM NaCl at pH = 7.0) was used to dilute the solutions of complexes **5a-c** in DMSO (1×10^{-2} M) to 1×10^{-4} M. The final solution contained about 1% DMSO. Measurements were taken after 24, 48, and 72 hours.

No significant changes were observed, except for a measurement error in the case of complex **5a**. However, the measurements after 24 and 72 hours are almost identical.

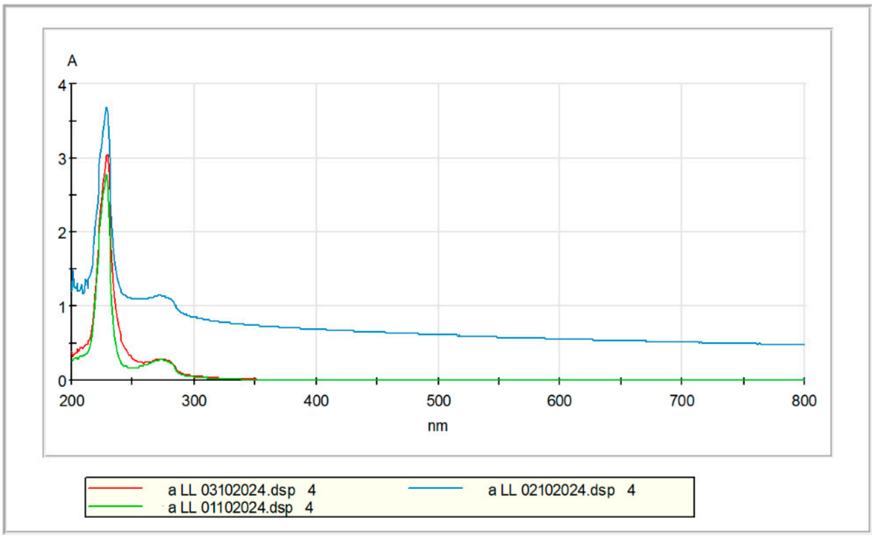

Figure S1-3: UV-Vis spectra of **5a** after 24 hours (green), 48 hours (blue) and 72 hours (red).

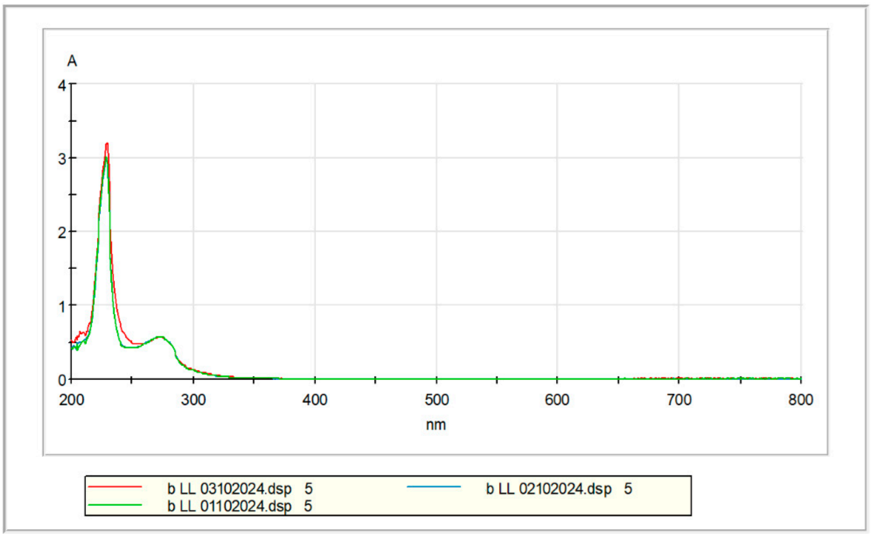

Figure S1-4: UV-Vis spectra of **5b** after 24 hours (green), 48 hours (blue) and 72 hours (red).

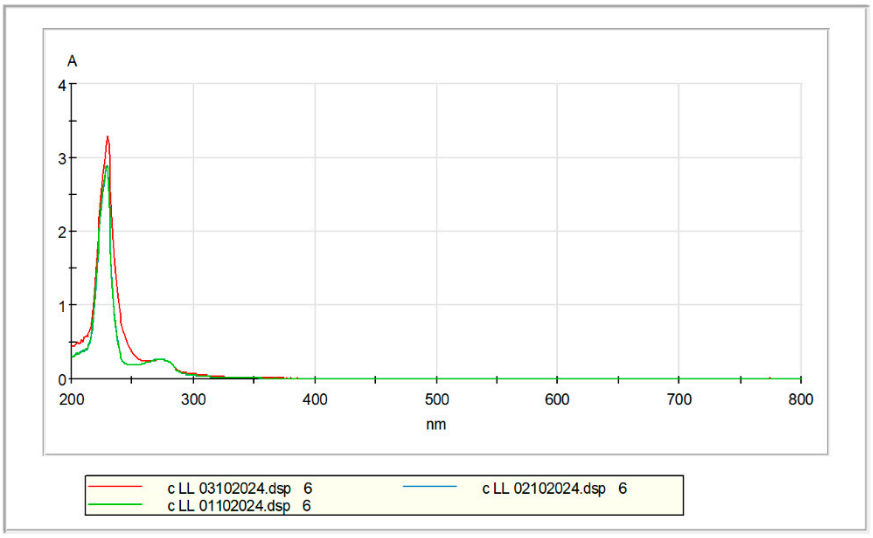

Figure S1-5: UV-Vis spectra of **5c** after 24 hours (green), 48 hours (blue) and 72 hours (red).

Stability studies were performed analogously at $1{\times}10^{-4}$ M in 99+% DMSO, with measurements done after 2 hours (red), 24 hours (blue), 48 hours (green), and 72 hours (purple). After 48 hours, a small shift to lower frequency (about 5 cm^{-1}) with no change in peak shape was observed, with no further change afterward.

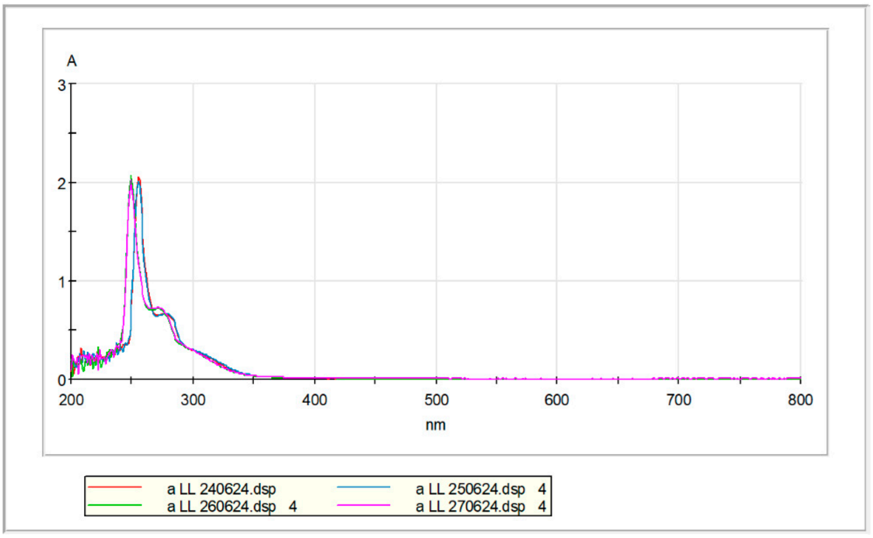

Figure S1-6: UV-Vis spectra of **5a** in DMSO

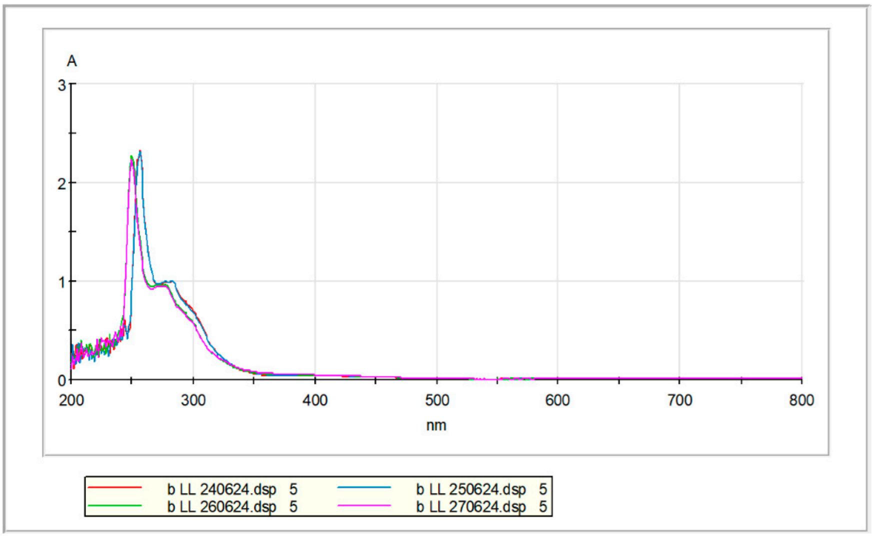

Figure S1-7: UV-Vis spectra of **5b** in DMSO

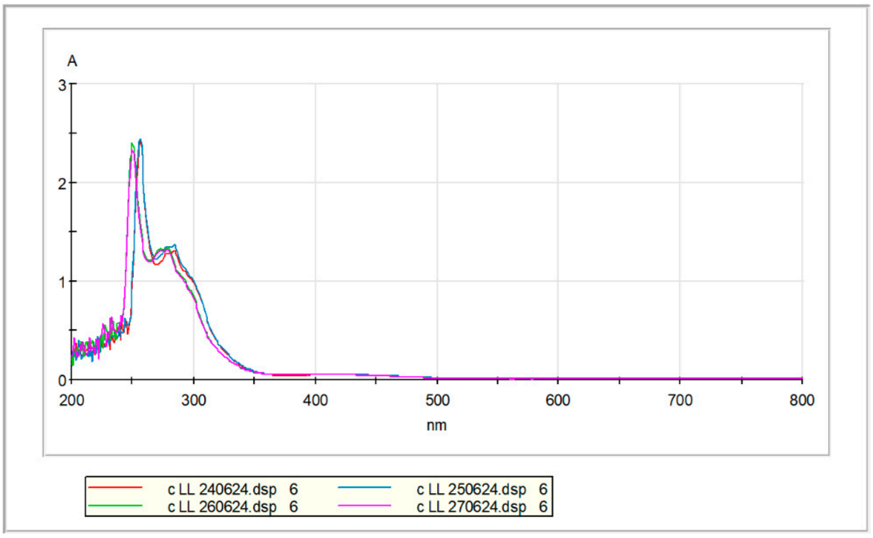

Figure S1-8: UV-Vis spectra of **5c** in DMSO

S1.2. UV-Vis complex formation studies

Comparison of UV-Vis spectra containing separate ligand **4c**, copper acetate and their mixture right mixing the solutions together:

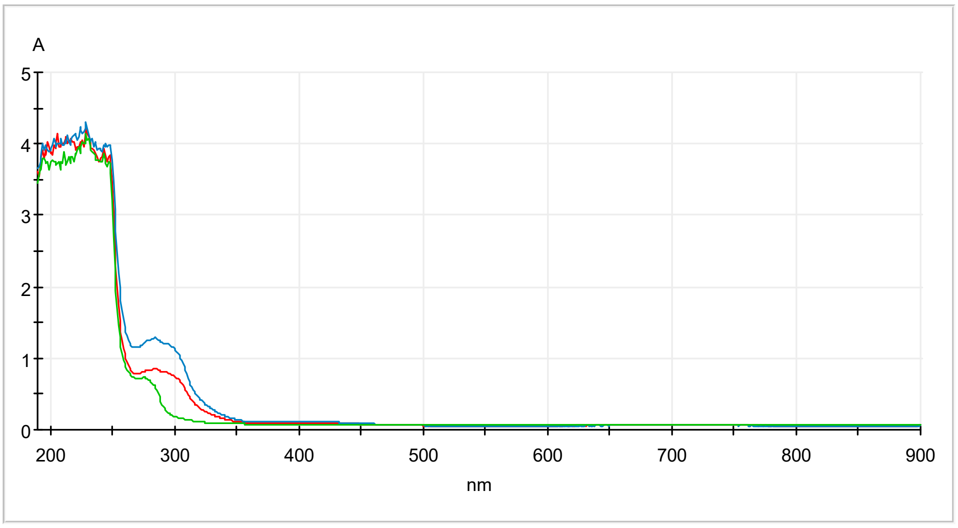

Figure S1-9: UV-Vis spectra comparing separate ligand and copper acetate combination (green), complex **5c** right after dissolution (red), and after 1 hour (blue)

1) Green spectrum: Two solutions of the ligand and of copper acetate in DMSO, each at 1×10^{-3} M. This was measured using two 250 µl cuvettes, each containing one of the components, held together in the detection beam path.

2) Red spectrum: A spectrum measured within 5 minutes after the two solutions were added together.

3) Blue spectrum: The mixture from 2) after 1 hour.

Comparing the ligand + copper salt combination with the complex spectra, we can see a shift in both spectra below. This suggests that the ligand coordinates to copper, as shown by differences between the green, blue, and red

spectra. After 1 hour, the maximum absorbance remains at the same wavelength. Thus, complex formation was also demonstrated in DMSO.

S1.4. MS spectra studies in solvent

To find out the composition of the complexes in DMSO, MS spectra studies were done. The solutions were measured around 24 hours after dissolving. All spectra were measured in positive mode.

In the spectra of complexes **5a-c**, we observed the complex as a mononuclear species with dissociation of the acetate and replacement by water molecules that most likely came from the solvent (peaks 342+344). No other higher forms, such as two or more Cu centers, were visible in solution, indicating that the binuclear complexes dissociate into mononuclear species upon dissolution. Also, no signs of DMSO binding to copper were detected. The results were expected, and the concentrations of the complexes used in the measurements reflected this and were calculated for mononuclear complexes [Cu L AcO].

In Figures S1-10, S1-11 and S1-12, the MS spectra for **5a-c** are shown. The spectra are almost identical; the only major difference is the peak ratios, with a slightly higher free-ligand peak in case **5a**.

All spectra showed the peaks at 244 and two close peaks at 342+344:

The peak at 244 belongs to the protonated ligand molecule $[L+H]^+$: $C_{15}H_{18}NO_2^+$ (calculated at 244.1338)

The peaks at 342+344 both belong to a diaqua mononuclear complex, $[Cu\ L+H\ (H_2O)_2]^+$: $C_{15}H_{21}NO_4^+$ (calculated at 342.0767 and 344.0749). The ratio around 100:45 is due to the two common copper isotopes, Cu-63 and Cu-65.

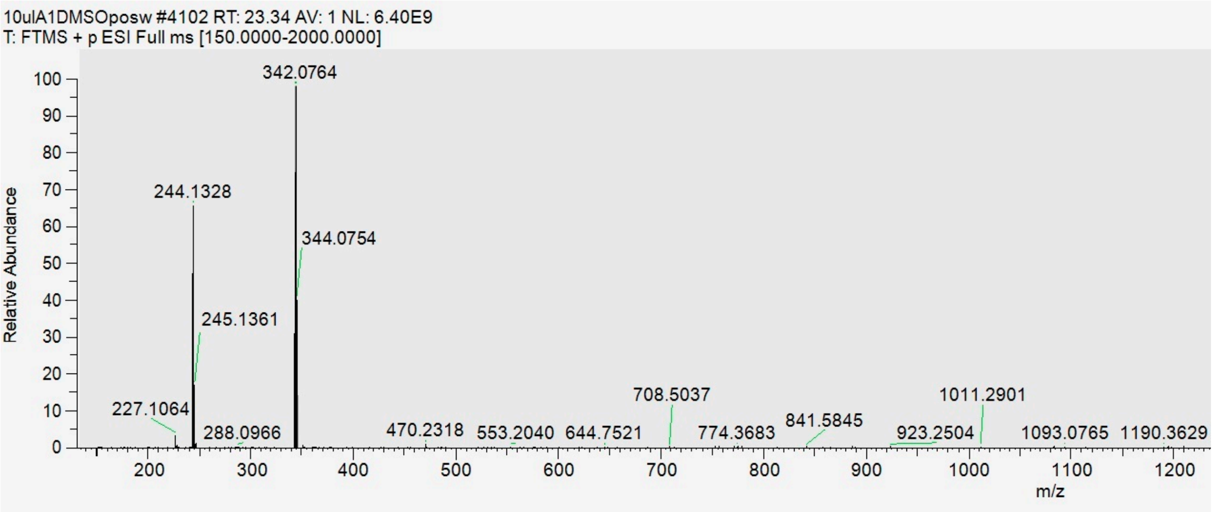

Figure S1-10: FTMS-ESI spectrum of **5a**

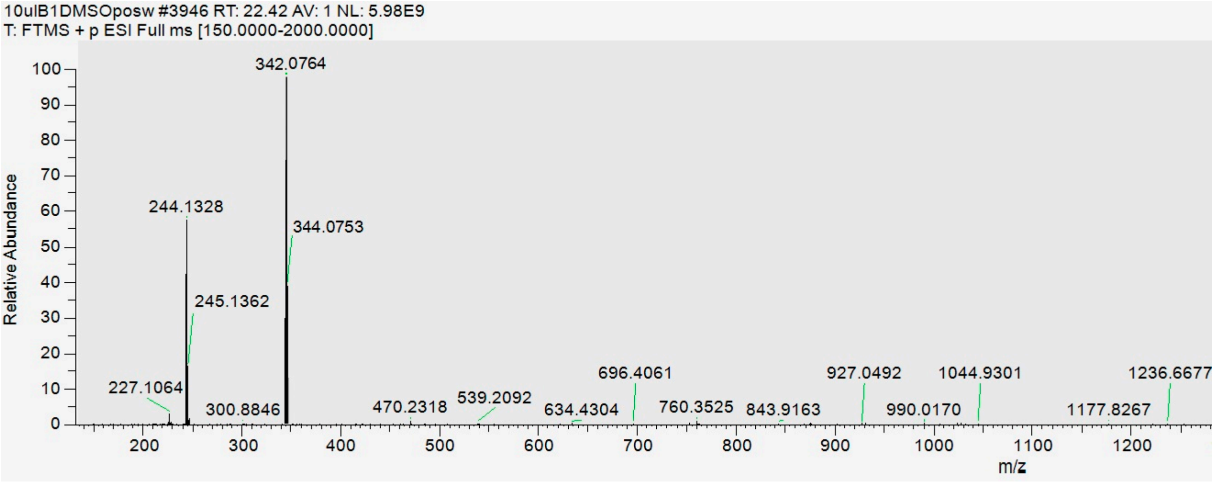

Figure S1-11: FTMS-ESI spectrum of **5b**

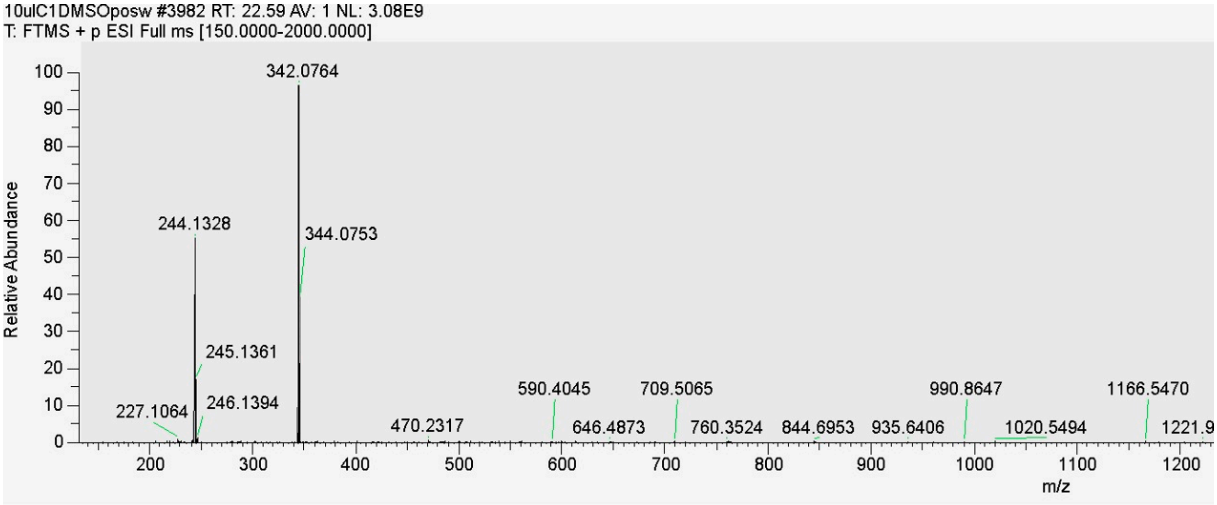

Figure S1-12: FTMS-ESI spectrum of **5c**

S1.5. IR spectra of prepared ligands and complexes

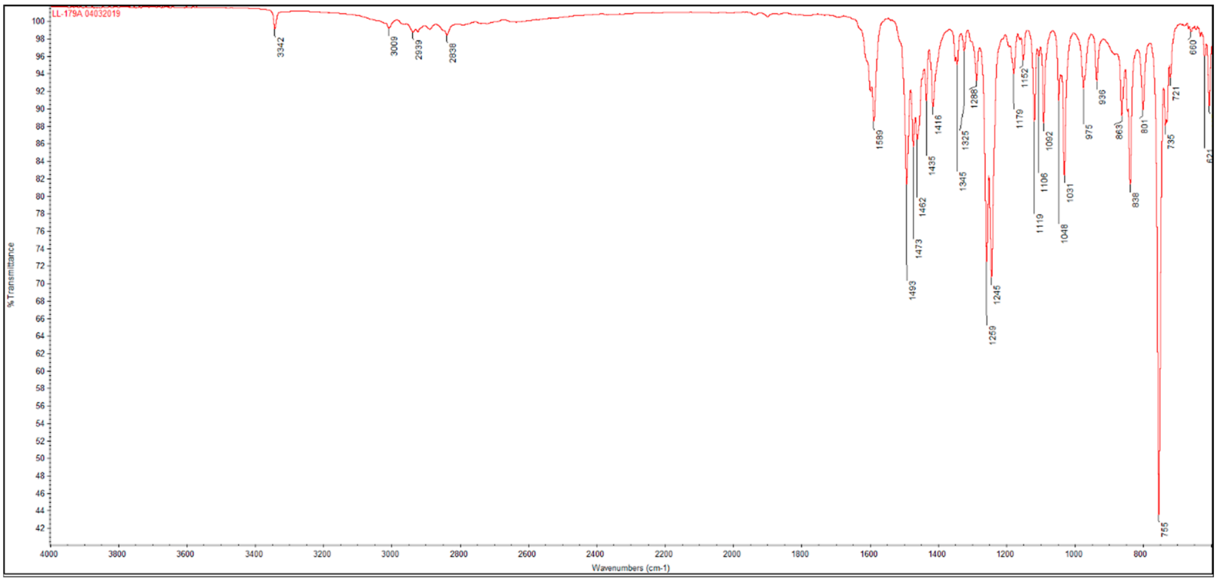

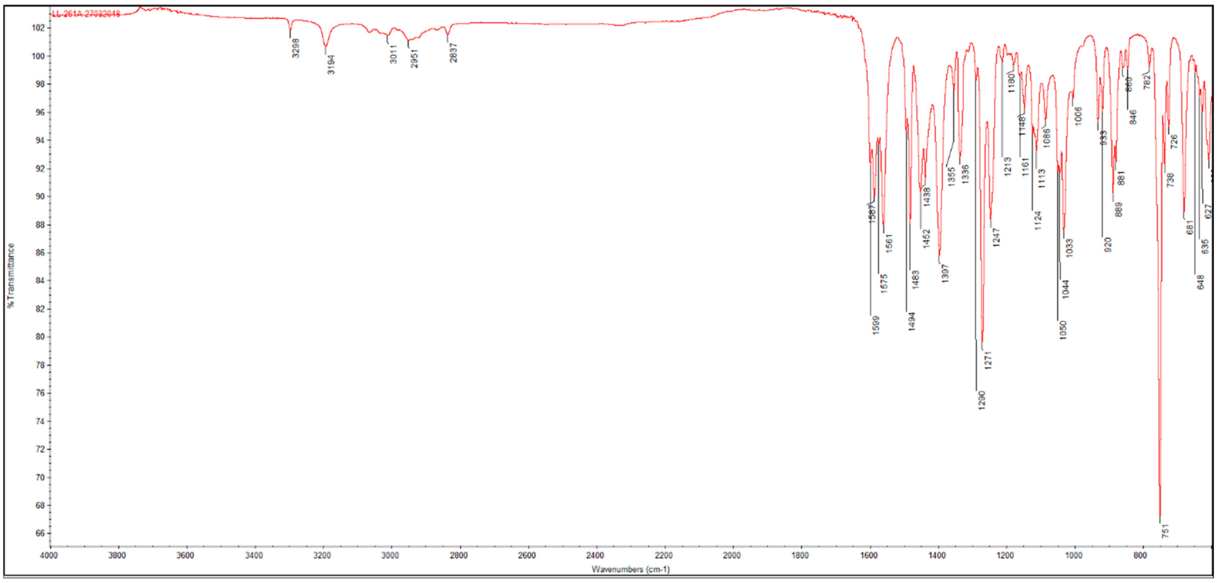

	$\nu_{N\text{-}H}$ stretch	$\nu_{N\text{-}H}$ bend	$\nu_{C\text{-}O}$ asym.	$\nu_{C\text{-}O}$ sym.	ν_{COO} asym.	ν_{COO} sym.
**4a**	3342	1589	1259,1245	1031		
**5a**	3194	1587	1271, 1336	1033	1561	1397

Figure S1-12: IR spectra of reduced Schiff base **4a** (up) and its complex **5a** (down) and the comparison of selected signals

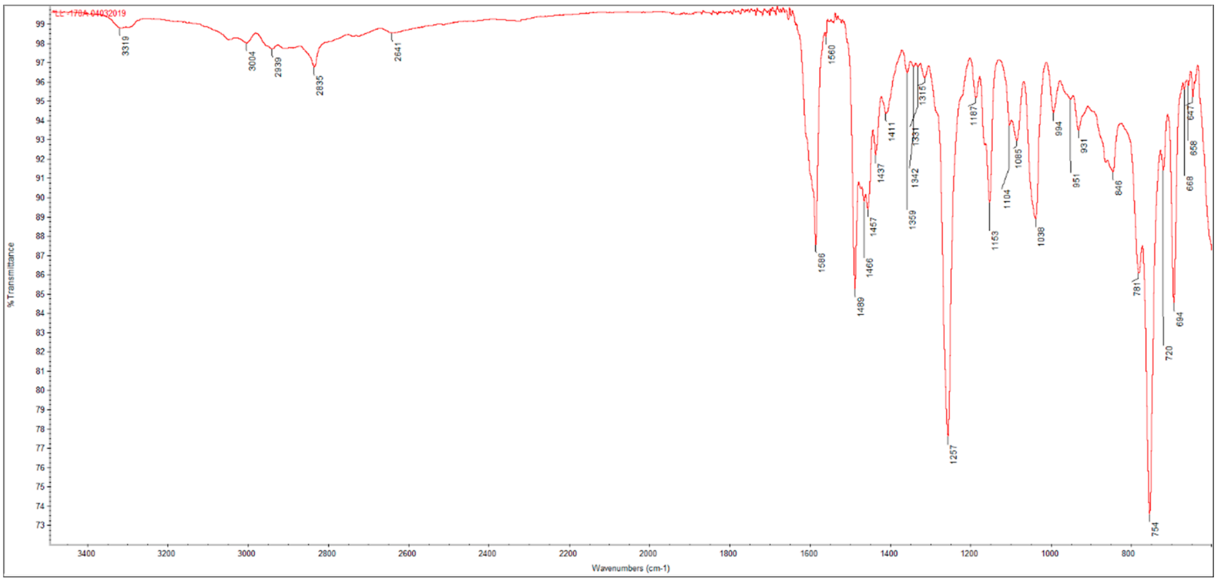

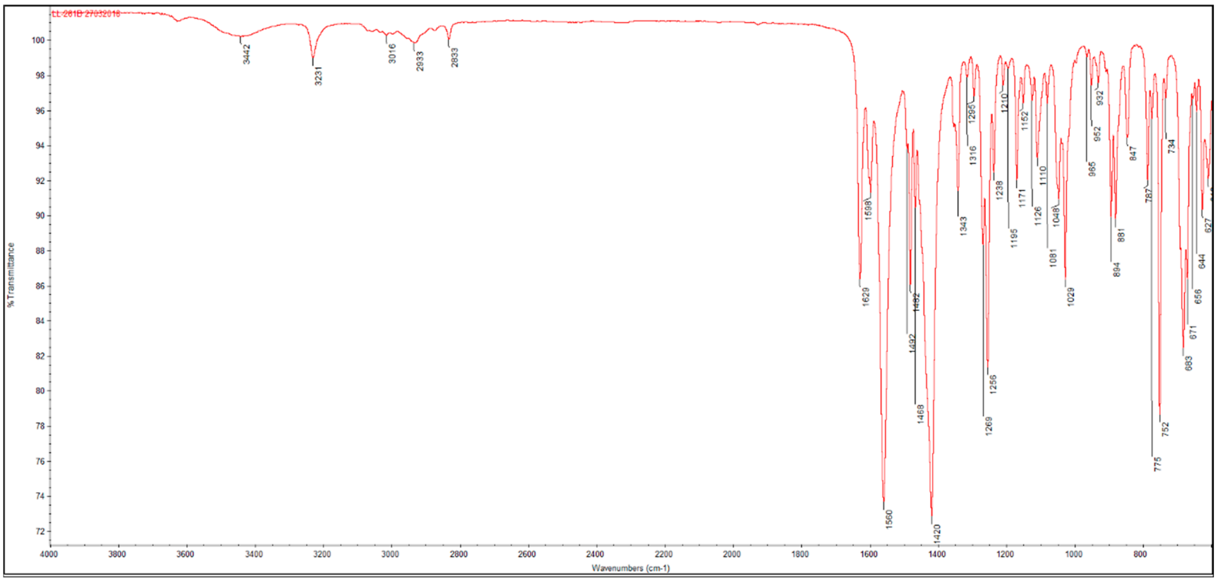

	ν_{N-H} stretch	ν_{N-H} bend	ν_{C-O} asym.	ν_{C-O} sym.	ν_{COO} asym.	ν_{COO} sym.
**4b**	3319	1586	1257	1038		
**5b**	3231	1598	1256, 1343	1029	1560	1420

Figure S1-13: IR spectra of reduced Schiff base **4b** (up) and its complex **5b** (down) and the comparison of selected signals

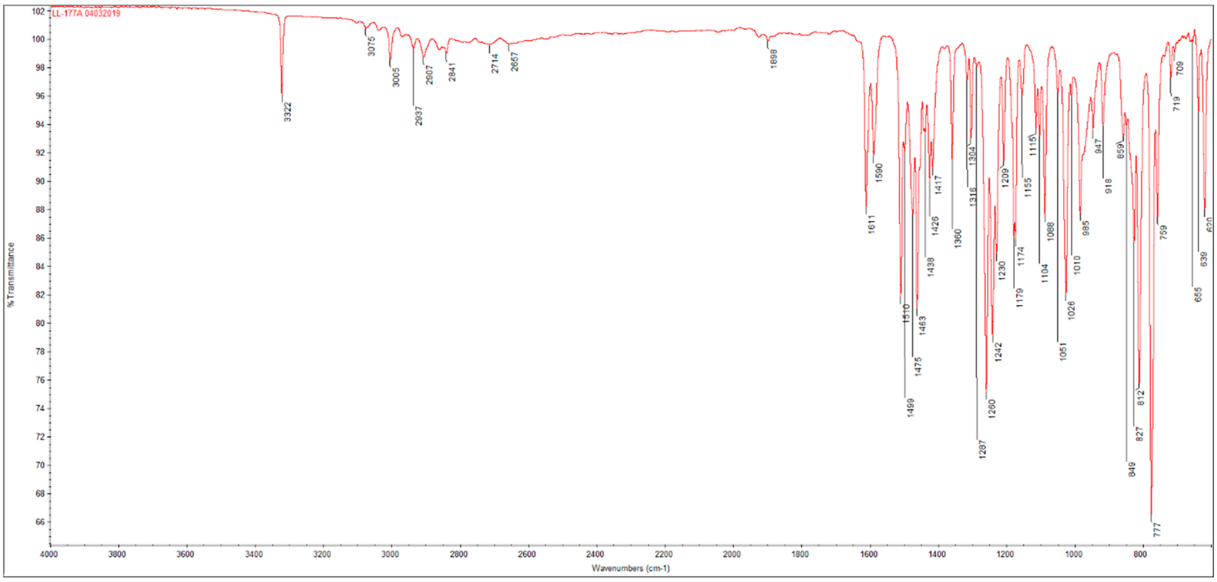

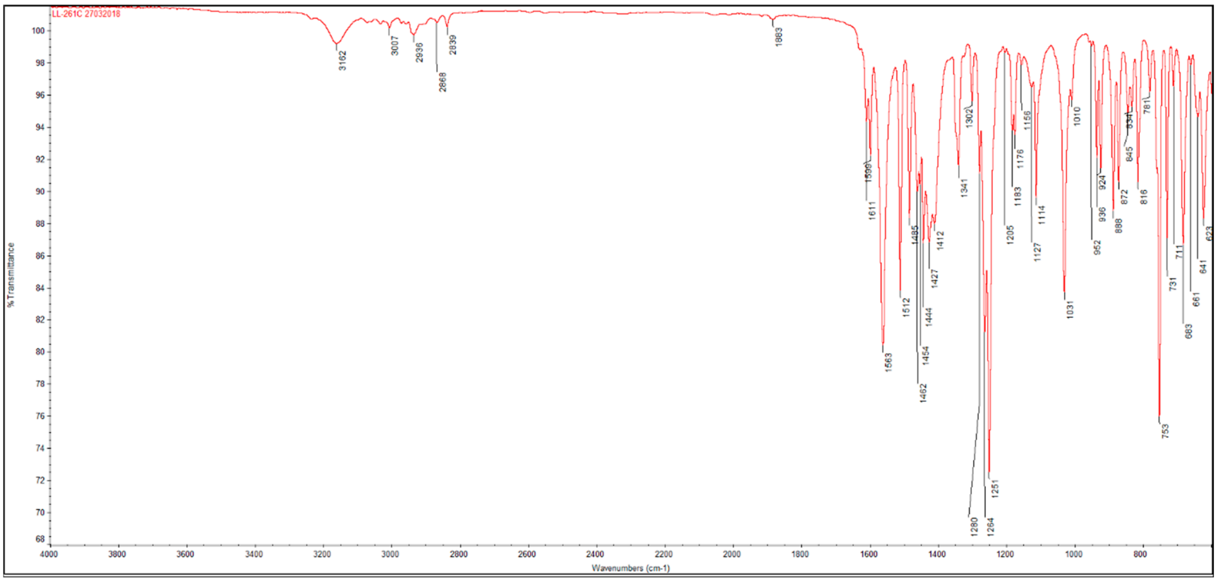

	ν_{N-H} stretch	ν_{N-H} bend	ν_{C-O} asym.	ν_{C-O} sym.	ν_{COO} asym.	ν_{COO} sym.
**4c**	3322	1590	1260, 1242	1026		
**5c**	3162	1599	1341, 1251	1031	1563	1427

Figure S1-14: IR spectra of reduced Schiff base **4c** (up) and its complex **5c** (down) and the comparison of selected signals

S1.5. EPR spectra of prepared complexes

EPR spectra were recorded using an X-band CW EPR spectrometer (Bruker EMX Plus; Bruker, Germany) with an ER4116DM dual-mode resonator (version 2.11; Bruker, Germany) that can operate in both TE102 mode (perpendicular) and TE012 mode (parallel). Most of the measurements were done in TE102 perpendicular mode. The instrument was operated using Bruker Acquisition proprietary software. For elevated-temperature measurements, we used a standard Bruker temperature unit. Measurements at low temperatures were performed in boiling nitrogen (77K) in a Dewar finger, using a nitrogen gas evaporator and temperature unit setup from Bruker. For solid-state measurements, a small amount of solid-state powder was placed into a thin-wall quartz EPR tube (internal diameter 3 mm).

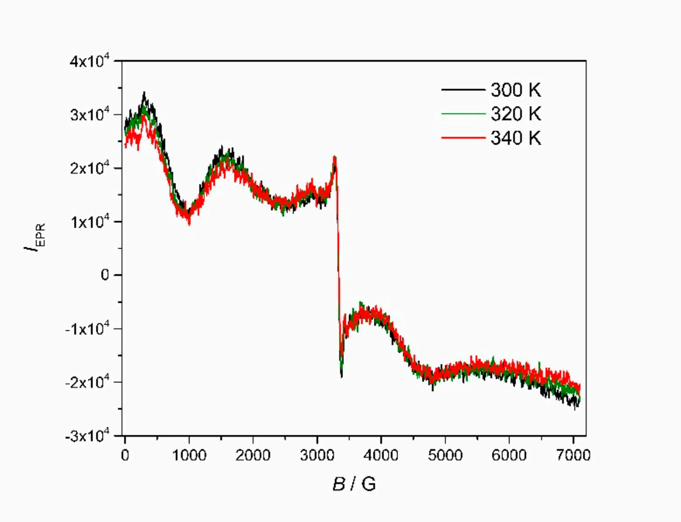

Figure S1-15: EPR spectra of complex **5a** recorded in perpendicular mode at different temperatures

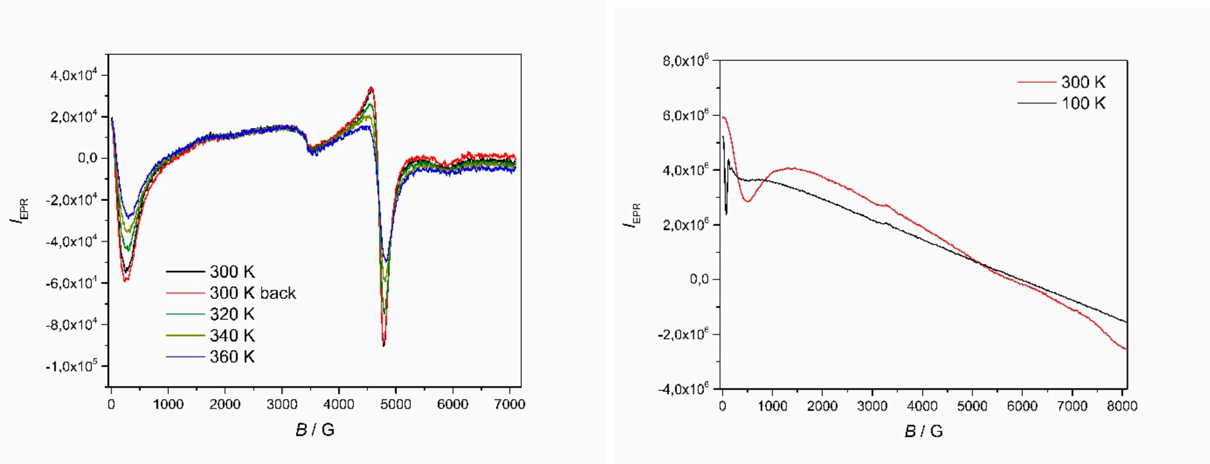

Figure S1-16: EPR spectra of complex **5b**; left: recorded in perpendicular mode at different temperatures, right: in parallel mode at different temperatures.

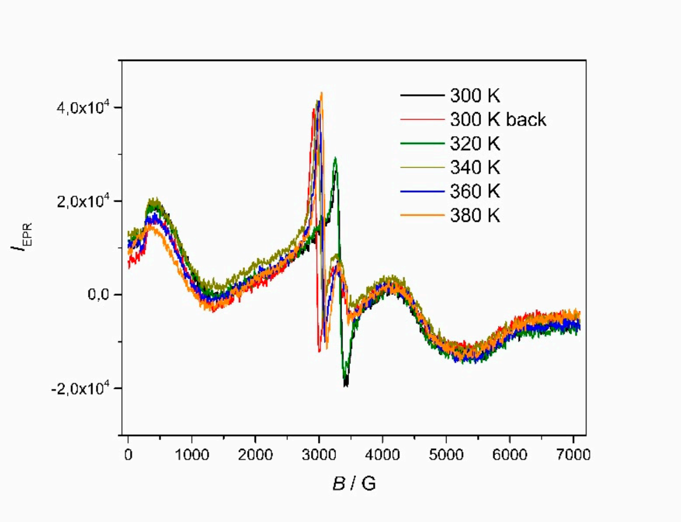

Figure S1-17: EPR spectra of complex **5c** recorded in perpendicular mode at different temperatures

S2. X-Ray Structure Analysis

The crystal structure of complex **5a** (**Figure S2-1)** is stabilized by a system of intermolecular and intramolecular hydrogen bonds and interactions (**Figure S2-2)** (**Table S2-1**).

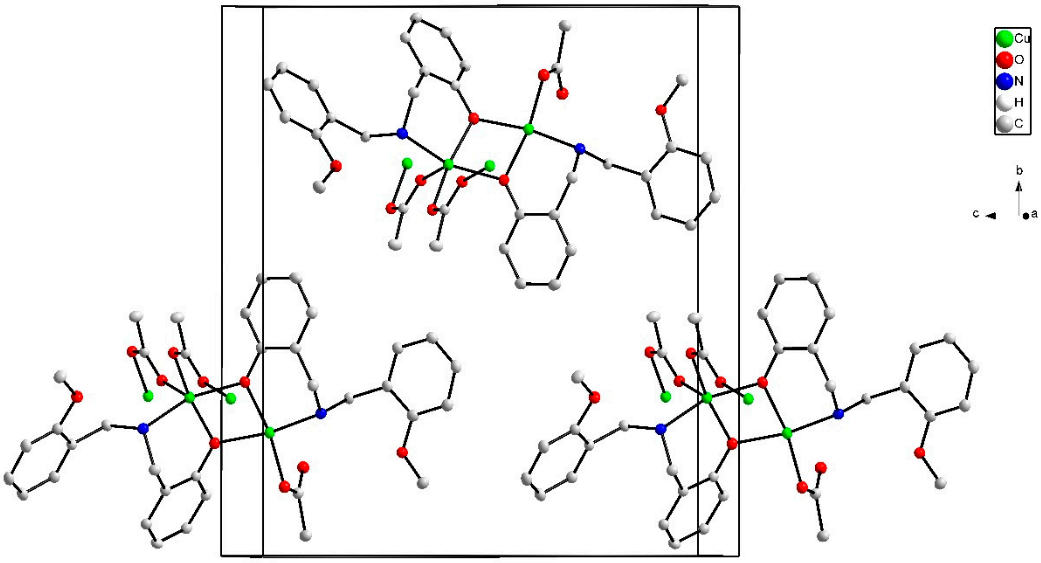

Figure S2-1: The molecular packing of complex **5a** viewed along the **a-**axis.

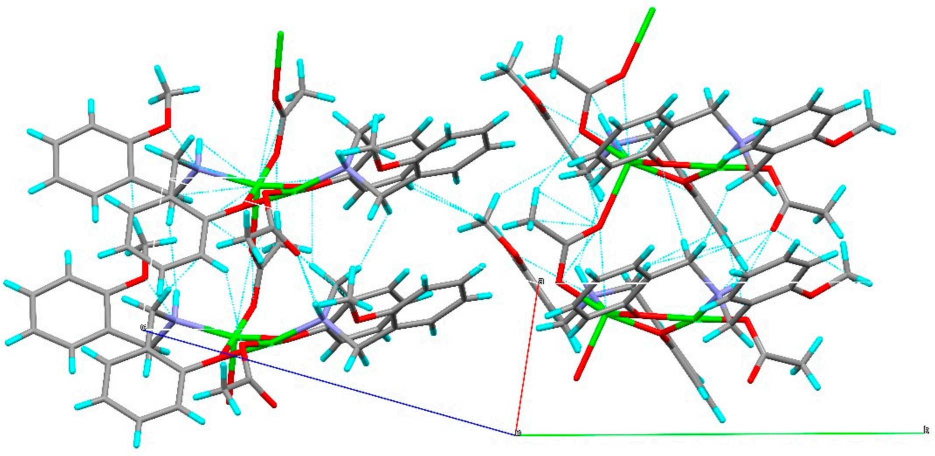

Figure S2-2: The molecular packing of complex **5a** with system hydrogen bonds and interactions viewed between the **a, b, c** axis.

Table S2-1. Hydrogen bonds and interactions for complex **5a**.

**D-H···A**	**D**	**H**	**A**	**d(D-H)/Å**	**d(H-A)/Å**	**d(D-A)/Å**	**D-H-A/°**	**Type of interaction**
N2-H2···O4	N2	H2	O4	1.00	2.28	2.962(11)	124.7	intramolecular
N1-H1···O6	N1	H1	O6*	1.00	1.99	2.896(10)	149.6	intermolecular
N1-H1···O2	N1	H1	O2	1.00	2.51	3.111(11)	118.5	intramolecular
C1-H1A···O6	C1	H1A	O6	0.99	2.63	3.334(12)	128.4	intramolecular
C8-H8B···O1*	C8	H8B	O1*	0.99	2.63	3.343(11)	129.3	intermolecular
C28-H28···O5**	C28	H28	O5**	0.95	2.51	3.263(11)	136.0	intermolecular
C34-H34B···O7*	C34	H34B	O7*	0.98	2.40	3.298(12)	152.1	intramolecular

Symmetry transformations used to generate equivalent atoms: (*)1+x,1+y, +z; (**)-1+x, +y, +z

The crystal structure of complex **5b** (**Figure S2-3)** is stabilized by a system of intermolecular hydrogen bonds and interactions (**Figure S2-4) (Table S2-2**).

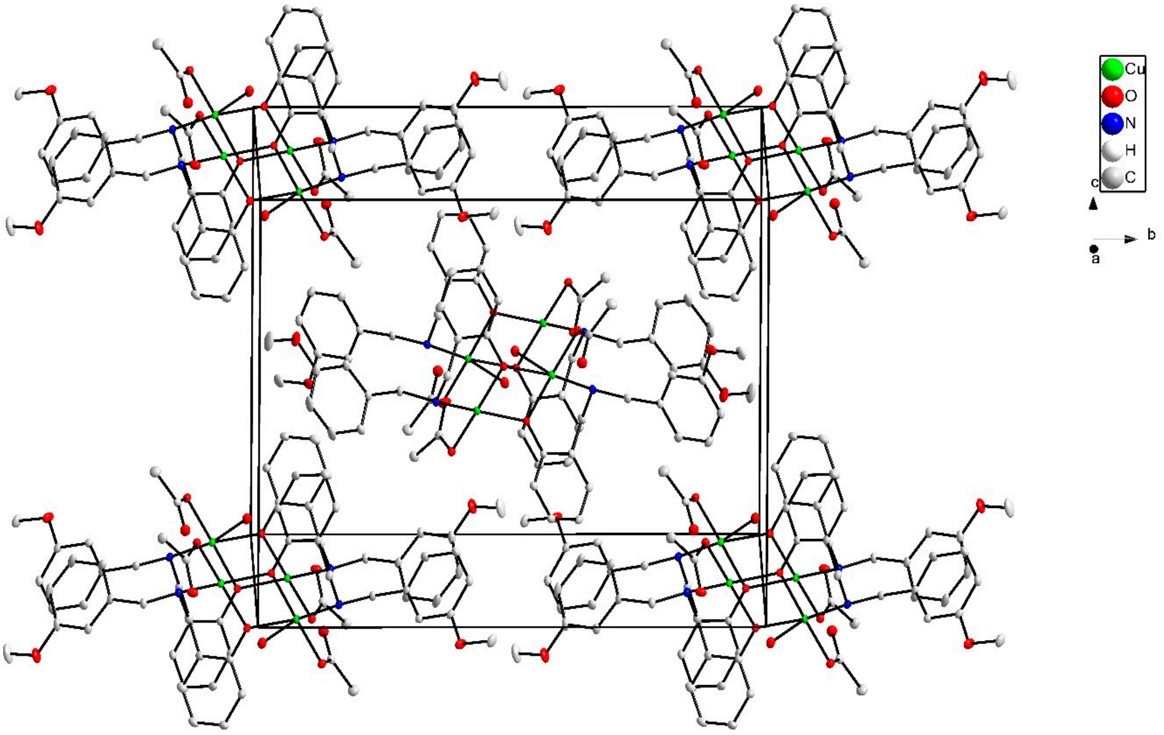

Figure S2-3: The molecular packing of complex **5b** viewed along the **a-**axis.

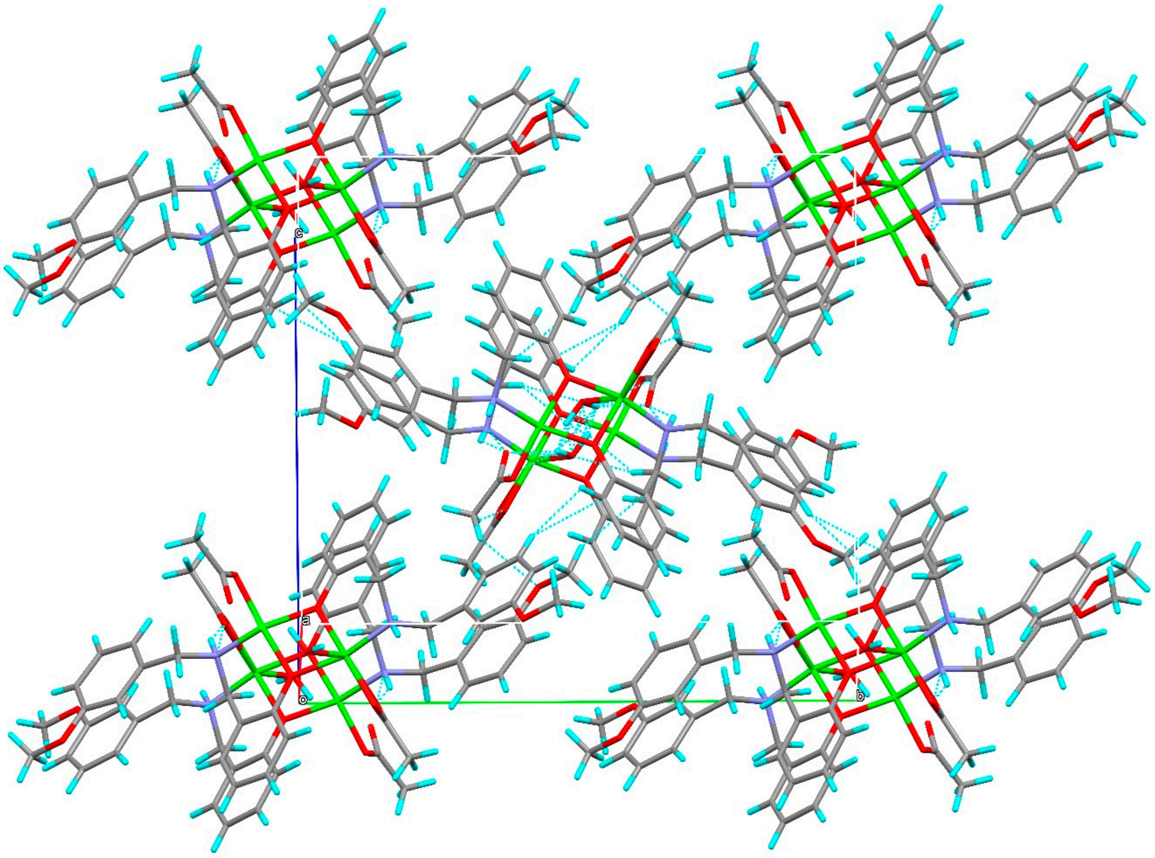

Figure S2-4: The molecular packing of complex **5b** with system hydrogen bonds and interactions viewed between the **a, b, c** axis.

Table S2-2. Hydrogen bonds and interactions for complex **5b**.

D-H···A	D	H	A	d(D-H)/Å	d(H-A)/Å	d(D-A)/Å	D-H-A/°	Type of interactions
N1-H1···O8*	N1	H1	O8*	1.00	2.00	2.848(2)	141.4	intermolecular
N2-H2···O6**	N2	H2	O6**	1.00	1.90	2.821(2)	151.9	intermolecular
C1-H1B···O5	C1	H1B	O5	0.99	2.66	3.202(2)	114.3	intramolecular
C1-H1B···O6	C1	H1B	O6	0.99	2.94	3.530(2)	119.2	intramolecular
C8-H8B···O9	C8	H8B	O9	0.99	2.92	3.492(6)	117.9	intermolecular
C12-H12···O2***	C12	H12	O2***	0.95	2.77	3.384(3)	122.7	intermolecular
C16-H16A···O7	C16	H16A	O7	0.99	2.72	3.283(2)	116.2	intramolecular
C16-H16A···O8	C16	H16A	O8	0.99	2.72	3.282(2)	116.5	intramolecular
C11-H11···O2***	C11	H11	O2***	0.95	2.75	3.368(3)	123.8	intermolecular
C34-H34A···O4****	C34	H34A	O4****	0.98	2.69	3.249(3)	116.4	intermolecular
C34-H34B···O5*	C34	H34B	O5*	0.98	2.63	3.550(3)	156.8	intermolecular
C22-H22···O6**	C22	H22	O6**	0.95	2.74	3.387(3)	125.6	intermolecular
C27-H27···O4*****	C27	H27	O4*****	0.95	2.80	3.471(3)	128.2	intermolecular
C28-H28···O5	C28	H28	O5	0.95	2.50	3.114(2)	122.7	intramolecular
C28-H28···O4*****	C28	H28	O4*****	0.95	2.90	3.514(3)	123.6	intermolecular
O9-H9A···O8	O9	H9A	O8	0.89	2.35	3.135(6)	148.2	intramolecular

Symmetry transformations used to generate equivalent atoms: (*)1-x,1-y,1-z; (**)2-x,1-y,1-z; (***)1/2+x, 3/2-y, -1/2+z; (****)-1/2+x, 1/2-y, -1/2+z; (*****)3/2-x, 1/2+y, 3/2-z

The crystal structure of complex **5c** (**Figure S2-5)** is stabilized by a system of intermolecular hydrogen bonds and interactions (**Figure S2-6)** (**Table S2-3**).

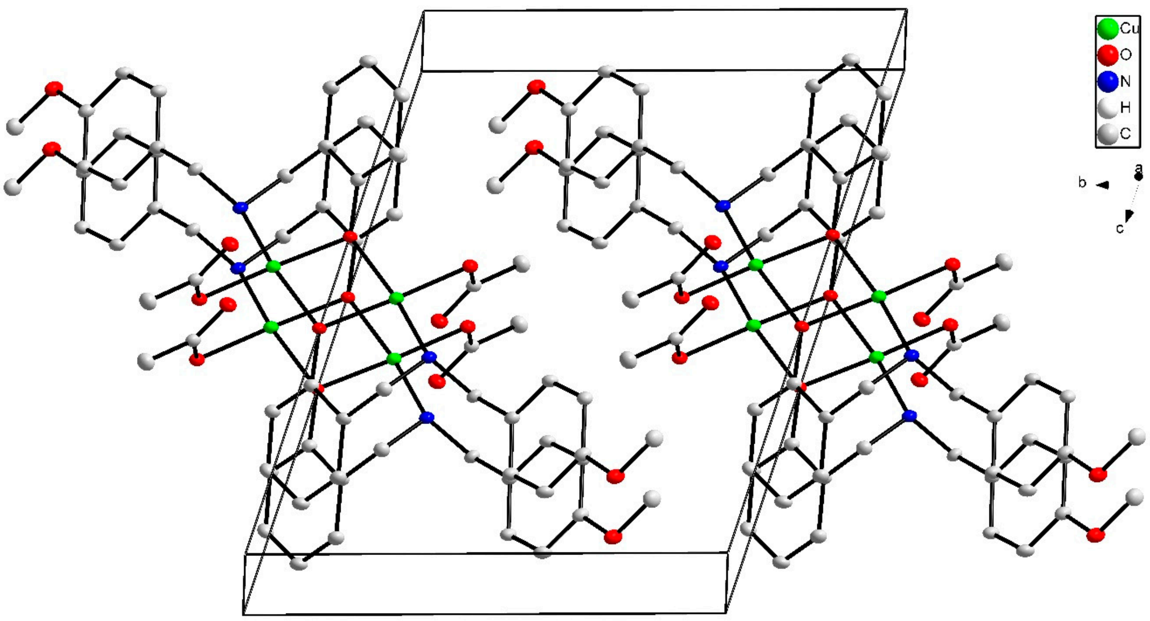

Figure S2-5: The molecular packing of complex **5c** viewed along the **a**-axis.

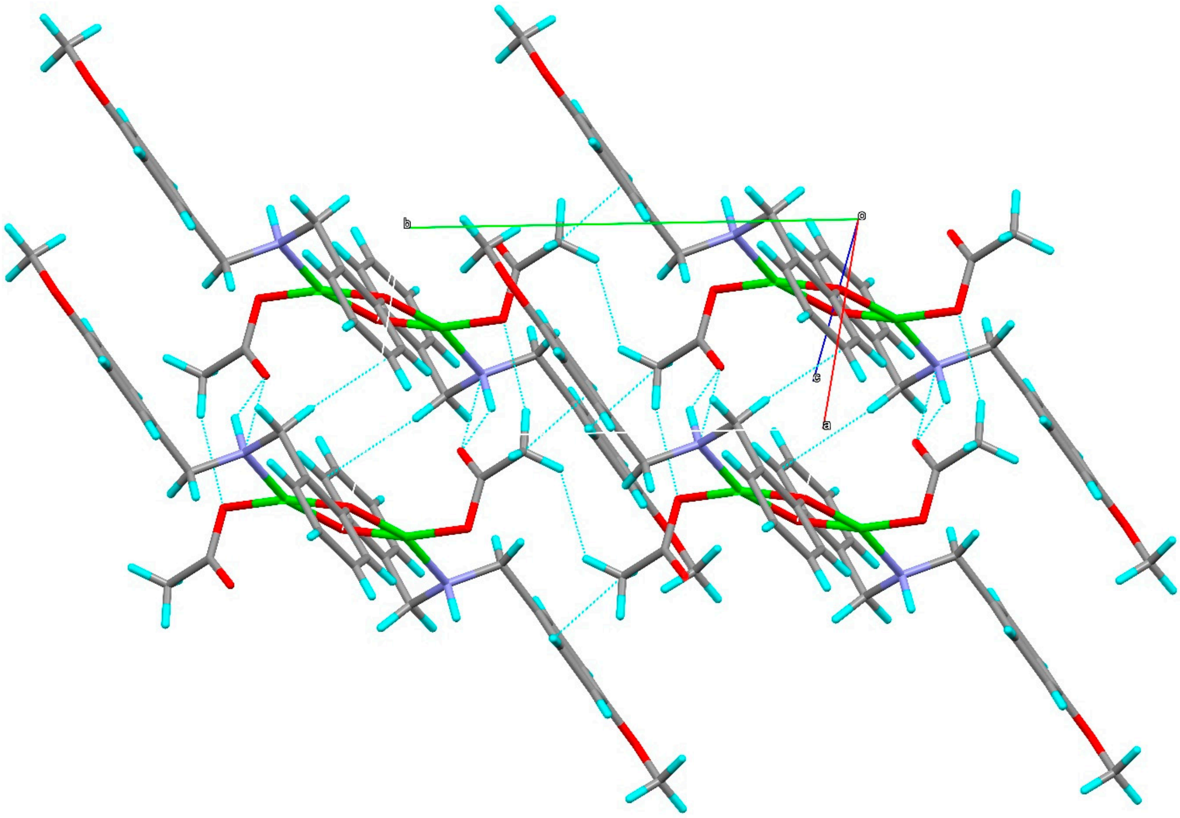

Figure S2-6: The molecular packing of complex **5c** with system hydrogen bonds and interactions viewed between the **a, b, c** axis.

Table S2-3. Hydrogen bonds and interactions for complex **5c**.

**D-H···A**	**D**	**H**	**A**	**d(D-H)/Å**	**d(H-A)/Å**	**d(D-A)/Å**	**D-H-A/°**	**Type of interaction**
N1-H1···O4*	N1	H1	O4*	1.00	1.97	2.797(6)	138.5	intermolecular
C17-H17B···O3**	C17	H17B	O3**	0.98	2.57	3.502(7)	158.7	intermolecular

Symmetry transformations used to generate equivalent atoms: (*)1+x, +y, +z; (**)-1+x, +y, +z;

Table S2-4. Selected bond lengths for complexes **5a-c**

Complex	Atom	Atom	Length/Å	Position	Atom	Atom	Length/Å	Position
	Cu1	O5	1.932(7)	eq.	Cu2	O7	1.941(6)	eq.
	Cu1	O3	1.953(7)	eq.	Cu2	O3	1.943(7)	eq.
**5a**	Cu1	O1	1.952(7)	eq.	Cu2	O1	1.989(7)	eq.
	Cu1	N1	1.996(8)	eq.	Cu2	N2	2.038(8)	eq.
	Cu1	Cu2	3.0421(17)	between	Cu2	O8*	2.291(6)	ax.
	Cu1	Cu2	3.0399(4)	ax.	Cu1	O9	2.262(5)	between
	Cu1	O5	1.9330(13)	eq.	Cu2	O1	1.9428(13)	eq.
**5b**	Cu1	O1	1.9357(13)	eq.	Cu2	O7	1.9593(13)	eq.
	Cu1	O3	1.9549(14)	eq.	Cu2	O3	1.9401(14)	eq.
	Cu1	N1	1.9989(15)	eq.	Cu2	N2	1.9827(15)	eq.
	Cu1	Cu1**	3.042(2)	between	Cu1	O3	1.969(4)	between
**5c**	Cu1	O1	1.944(3)	eq.	Cu1	N1	1.989(4)	eq.
	Cu1	O1**	1.954(3)	eq.	Cu1	C16	2.541(6)	eq.

Symmetry transformations used to generate equivalent atoms: (*)1-x,1-y, -z; (**)-x, 2-y, 1-z

Table S2-5. Selected angles for complexes **5a-c**

Complex	Atom	Atom	Atom	Angle/°	Atom	Atom	Atom	Angle/°
	O5	Cu1	Cu2	129.3(2)	O3	Cu1	N1	166.7(3)
	O5	Cu1	O3	94.2(3)	O1	Cu1	Cu2	39.9(2)
	O5	Cu1	O1	169.1(3)	O1	Cu1	O3	76.1(3)
	O5	Cu1	N1	95.7(3)	O1	Cu1	N1	93.2(3)
	O3	Cu1	Cu2	38.6(2)	N1	Cu1	Cu2	128.8(2)
	O8*	Cu2	Cu1	111.08(17)	O7	Cu2	N2	91.0(3)
5a	O7	Cu2	Cu1	130.2(2)	O3	Cu2	Cu1	38.8(2)
	O7	Cu2	O8*	99.3(2)	O3	Cu2	O8*	93.3(3)
	O7	Cu2	O3	166.5(3)	O3	Cu2	O1	75.5(3)
	O7	Cu2	O1	96.7(3)	O3	Cu2	N2	92.5(3)
	O1	Cu2	Cu1	39.04(19)	N2	Cu2	Cu1	122.7(2)
	O1	Cu2	O8*	104.0(3)	N2	Cu2	O8*	95.6(3)
	O1	Cu2	N2	157.4(3)	Cu2	O3	Cu1	102.7(3)
					Cu1	O1	Cu2	101.0(3)
	O5	Cu1	O1	172.99(6)	O1	Cu1	O3	76.73(6)
	O5	Cu1	O3	101.59(6)	O1	Cu1	O9	87.36(15)
5b	O5	Cu1	O9	98.70(15)	O3	Cu1	O9	72.96(15)
	O5	Cu1	N1	90.44(6)	N1	Cu1	Cu2	128.93(4)
	O1	Cu1	N1	92.46(6)	O1	Cu1	Cu2	38.48(4)

**5b**	N1	Cu1	O9	95.78(15)	O5	Cu1	Cu2	140.10(4)
	O3	Cu1	N1	164.51(7)	O3	Cu1	Cu2	38.52(4)
	O9	Cu1	Cu2	74.06(14)	O1	Cu2	Cu1	38.31(4)
	O1	Cu2	O7	97.86(5)	O1	Cu2	N2	159.39(6)
	O3	Cu2	O1	76.91(6)	O7	Cu2	Cu1	133.27(4)
	O3	Cu2	O7	165.31(7)	O7	Cu2	N2	93.95(6)
	O3	Cu2	N2	95.30(6)	O3	Cu2	Cu1	38.87(4)
	N2	Cu2	Cu1	132.77(5)	Cu1	O1	Cu2	103.21(6)
	Cu2	O3	Cu1	102.60(6)				
**5c**	O1**	Cu1	Cu1**	38.60(10)	O1	Cu1	Cu1**	38.84(10)
	O1	Cu1	O1**	77.44(15)	O1	Cu1	N1	93.64(16)
	O1**	Cu1	O3	95.65(14)	O3	Cu1	N1	95.21(16)
	Cu1	O1	Cu1**	102.56(15)	N1	Cu1	Cu1**	131.11(13)
	O3	Cu1	Cu1**	133.56(11)	O1**	Cu1	N1	163.78(17)
	O1	Cu1	O3	168.26(16)				

Symmetry transformations used to generate equivalent atoms: (*)1-x,1-y, -z; (**)-x, 2-y, 1-z

The CIF files were uploaded to CCDC (Cambridge Crystallographic Database Center) under these deposition numbers: **2367652, 2576586,** and **2576587**:

Summary of Data - **Deposition Number 2367652**

Compound Name:

Data Block Name: **data_lin_02**

Unit Cell Parameters: a 10.99140(10) b 18.4756(2) c 16.1150(2) P21/n

----

Summary of Data - Deposition Number 2576586

Compound Name:

Data Block Name: **data_ll295a2_**

Unit Cell Parameters: a 4.9823(2) b 18.9202(8) c 17.0362(6) Pn

----

Summary of Data - **Deposition Number 2576587**

Compound Name:

Data Block Name: **data_ll295c_**

Unit Cell Parameters: a 5.1854(4) b 11.4767(6) c 14.7309(7) P-1

S3. DNA Cleavage Assay

The ability of copper(II) complexes to cleave DNA was assessed by monitoring the conversion of supercoiled plasmid DNA (Form I) to open-circular DNA (Form II) by agarose gel electrophoresis to separate the cleavage products.

In general, 250 ng of pBBR 322 DNA plasmid (in 2.5×10^{-2} M Tris-HCl buffer solution, pH 7.5) was treated with different concentrations (1×10^{-3}, 3×10^{-3}, 5×10^{-3} M) of the copper(II) complex (dissolved in DMSO) for 3 h at 37 °C. After that, each reaction was quenched by adding 4 µL of a Smart Glow Loading Dye® (loading buffer solution with 0.01% Safe Green dye, 50% glycerol, and 0.25 M EDTA, pH 8.0) and the sample subjected to electrophoresis (MyGel Mini electrophoresis system®, Accuris) on a 0.9 % agarose gel containing a TAE buffer (4×10^{-2} M Tris, 2×10^{-2} M acetic acid, and 1×10^{-3} M EDTA) at a voltage of 55 V/ 40 mA for approximately 1.5 h. As a ”running buffer”, TAE buffer (4×10^{-2} M Tris, 2×10^{-2} M acetic acid, and 1×10^{-3} M EDTA) was used. The resulting gels were visualized under UV light and scanned with a mobile device, an Apple iPhone 11 cam 26 mm f1.8. The DNA cleavage activities were quantified using gelQUANT software (version 4.3.9, Cleaver Scientific, UK).

In the following tables, the percentage (%) expression of the amount of pDNA that has passed through the effect of the nuclease activity of the copper complex from the uncleaved supercoiled form I to the cleaved form II (open circular pDNA). Quantitative evaluation was performed using the "gelQUANT software" (Cleaver Scientific, UK). $V_{\text{(tested sample)}}$ = 20 ul ; $c_{\text{(pDNA)}}$ = 250 ng.

Table S3-1: Quantitative evaluation of nuclease activity of the copper complex **5a**.

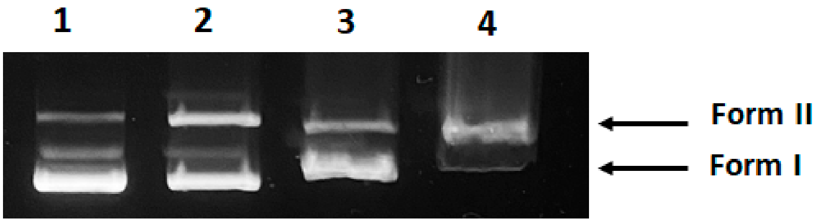

ELFO line	Complex 5a	Incubation time - 3h		
		**Form I. (%)**	**Form II. (%)**	**Form III. (%)**
**1**	**C**	89,8 (224,5 ng)	10,2 (25,5 ng)	0
**2**	**1×10^{-3}**	63,9 (159,75 ng)	36,1 (90,25 ng)	0
**3**	**3×10^{-3}**	75,6 (189 ng)	24,4 (61 ng)	0
**4**	**5×10^{-3}**	5,5 (13,75 ng)	94,5 (236,25 ng)	0

Table S3-2: Quantitative evaluation of nuclease activity of the copper complex **5b**.

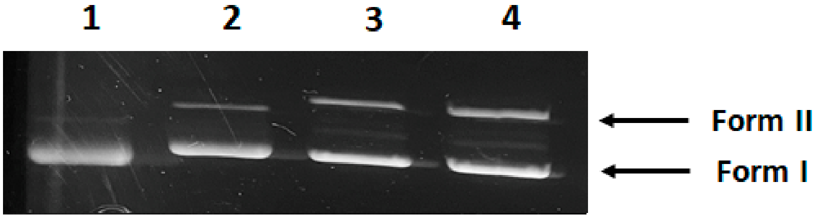

**ELFO line**	**Complex 5b**	**Incubation time - 3h**		
		**Form I. (%)**	**Form II. (%)**	**Form III. (%)**
**1**	**C**	100 (250 ng)	0	0
**2**	**1 × 10^{-3}**	86,1 (215,25 ng)	13,9 (34,75 ng)	0
**3**	**3 × 10^{-3}**	70,2 (175,5 ng)	30,8 (77 ng)	0
**4**	**5 × 10^{-3}**	62,8 (157 ng)	37,2 (93 ng)	0

Table S3-3: Quantitative evaluation of nuclease activity of the copper complex **5c**.

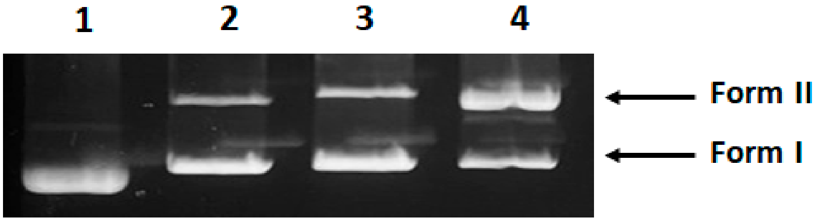

**ELFO line**	**Complex 5c**	**Incubation time - 3h**		
		**Form I. (%)**	**Form II. (%)**	**Form III. (%)**
**1**	**C**	100 (250 ng)	0	0
**2**	**1 × 10^{-3}**	73,6 (184 ng)	26,4 (66 ng)	0
**3**	**3 × 10^{-3}**	69,8 (174,5 ng)	30,2 (75,5 ng)	0
**4**	**5 × 10^{-3}**	35,7 (89,25 ng)	64,3 (160,75 ng)	0

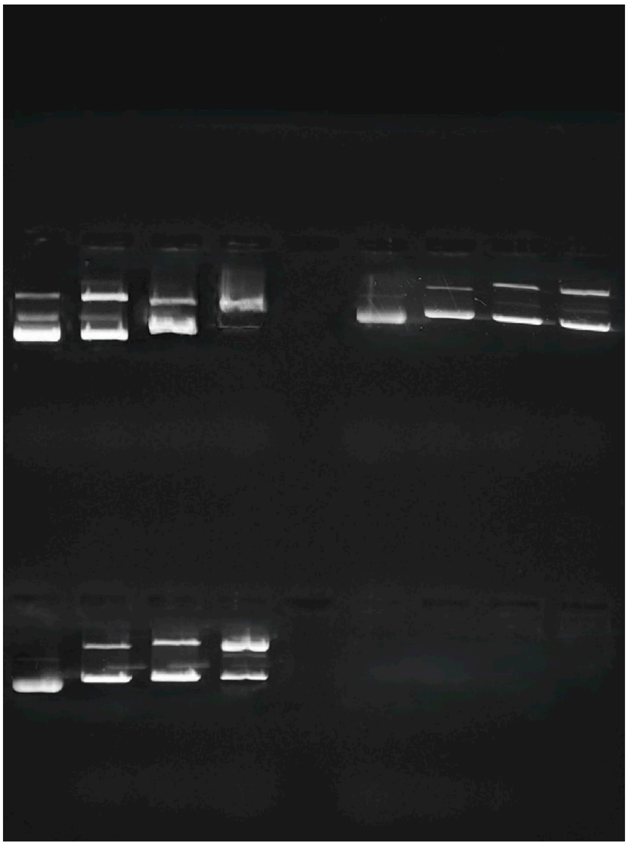

Figure S3-1: Original Western blot gel scan: upper left lines 1 – 4 complex **5a**, upper right lines 5 - 9 complex **5b**, lower left lines 1 – 4 complex **5c**.

S4. DNA Binding Assays

S4.1. DNA binding study by absorption titration

The interactions of prepared complexes **5a-c** with ct-DNA were studied by UV-Vis-monitored titration. Measurements were performed in citrate buffer (1.5×10^{-3} M sodium citrate and 0.15 M NaCl at pH 7.0) at room temperature. The Cu(II) complexes were dissolved in DMSO, from which working solutions were prepared by diluting with citrate buffer at a concentration of 1×10^{-4} M. Increasing concentrations of ct-DNA were then added to a buffer/DMSO solution (<1% DMSO) of the corresponding complex.

For the values of the binding strength of the interaction between the complex and the DNA molecule, binding constants K_b were calculated by the ratio of the slope to the y-intercept in plots of [DNA]/(ε_A-ε_f) versus [DNA], according to the Wolfe-Shimer equation (Eq. 1):

$$\frac{[DNA]}{(\varepsilon_A-\varepsilon_f)} = \frac{[DNA]}{(\varepsilon_b-\varepsilon_f)} + \frac{1}{K_b(\varepsilon_b-\varepsilon_f)} \qquad (1, S4\text{-}1)$$

where [DNA] is the concentration of DNA in base pairs, ε_f is the extinction coefficient for the free complex at the corresponding λ_{max}, $\varepsilon_A = A_{obsd}$/[compound], and ε_b is the extinction coefficient for the complex in the fully bound form.

Control measurements with DMSO showed no changes in the ct-DNA spectra.

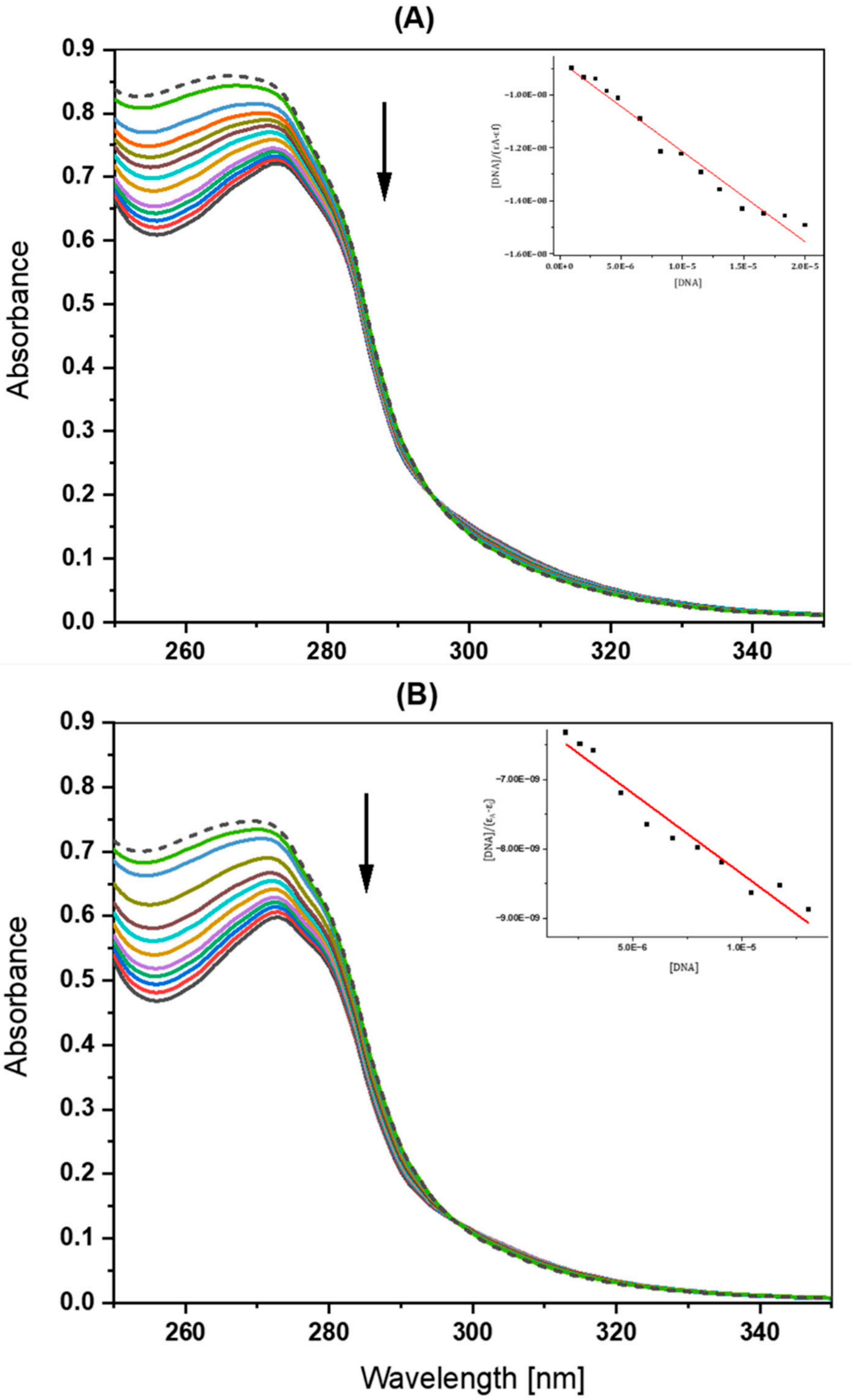

Figure S4-1: UV-Vis spectra of DMSO solution of complex **5b** (A) and **5c** (B) in the absence and presence of increasing amounts of ct-DNA (r = [DNA]/[complex] = 0-2.1). The arrows show changes in intensity upon the addition of increasing amounts of ct-DNA (concentration increment 2×10^{-6} M indicated by different colors of the curves). Insert graph: Plots [DNA]/(ε_A-ε_f) *versus* [DNA] according to the Wolfe- Shimer equation.

S4.2. Competitive studies with EB-DNA

Competitive fluorescence-binding experiments were carried out to assess the displacement of ethidium bromide (EB) from the EB–DNA complex.

The EB-DNA adduct was prepared by adding 20 μM EB and 54 μM ct-DNA in citrate buffer (pH = 7.0). The possible intercalating effect of the compounds was studied by adding increasing concentrations of the corresponding complexes **5a-c** ($c = 1\times10^{-3}$ M) to a solution of the DNA-EB complex. The added complex concentration ranged from 0 to 200 μM. Fluorescence emission spectra were recorded in the range 550-800 nm with an excitation wavelength of 515 nm. The complexes showed no fluorescence emission bands in their own solution or in the presence of ct-DNA or EB. Quenching of the EB-DNA emission band by compounds **5a-c** was calculated via the Stern-Volmer equation (Eq. 2-4).

The extent of the inner-filter effect can be roughly estimated with the following equation:

$$I_{corr} = I_{meas} \times 10^{\frac{\varepsilon(\lambda exc)cd}{2}} \times 10^{\frac{\varepsilon(\lambda em)cd}{2}} \qquad (2, S4\text{-}2)$$

where I_{corr} = corrected intensity, I_{meas} = the measured intensity, c = the concentration of the quencher, d = the cuvette (1 cm), $\varepsilon_{(\lambda exc)}$ and $\varepsilon_{(\lambda em)}$ = the ε of the quencher at the excitation and the emission wavelengths, respectively, as calculated from the UV-Vis spectra of the complexes. The inner filter effect was evaluated with Eq. 2, was found to be negligible and could not affect the measurements.

The quenching of the EB-DNA emission band by compounds was calculated via the Stern-Volmer equation:

$$\frac{Io}{I} = 1 + k_q\tau_0[Q] = 1 + K_{SV}[Q] \qquad (3, S4\text{-}3)$$

where Io = the initial fluorescence intensity of the EB-DNA adduct, and I is the fluorescence intensity of the EB-DNA adduct after the addition of the quencher (studied complexes), k_q= the quenching rate constant, K_{SV} = the Stern-Volmer constant, τ_o = the average fluorescence lifetime of EB-DNA adduct (23×10^{-9} s), [Q] = the concentration of the quencher, respectively, K_{SV} (in M^{-1}) can be obtained by the slope of the Stern-Volmer plot, and k_q (in $M^{-1}\ s^{-1}$) may be calculated from equation 4:

$$K_{SV} = k_q\tau_0 \qquad (4, S4\text{-}4)$$

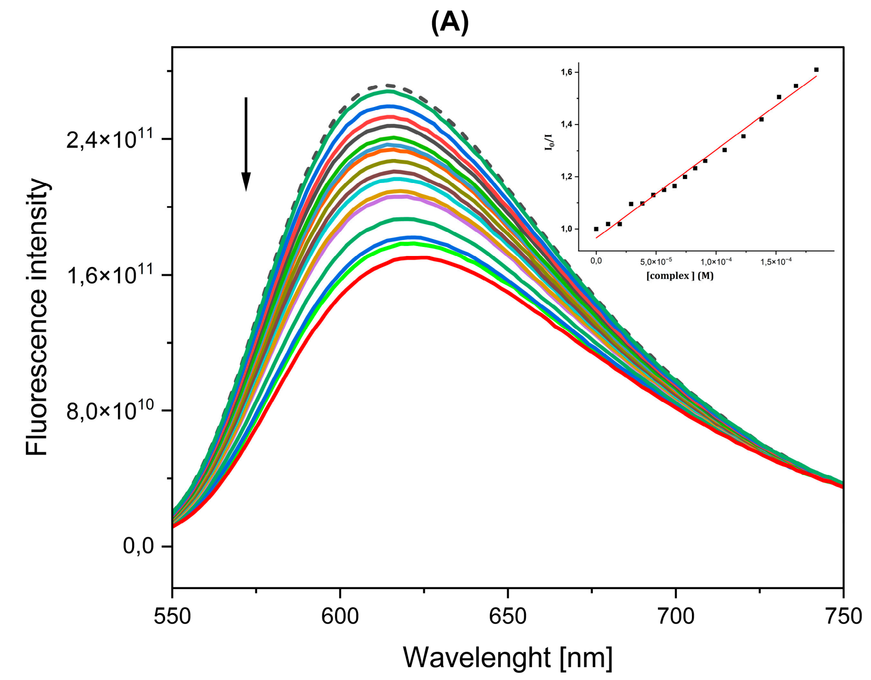

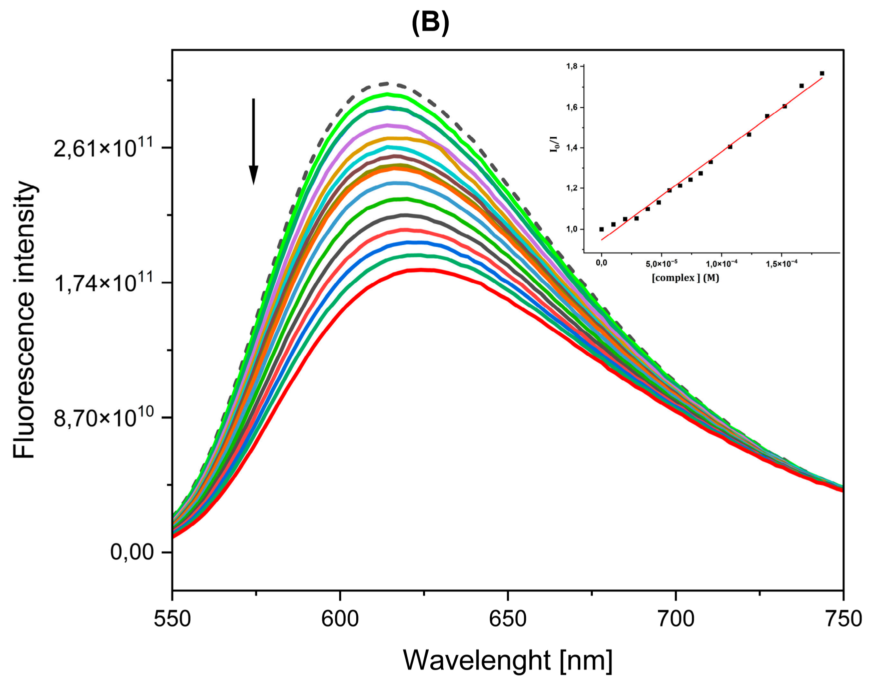

Figure S4-2 Emission fluorescence spectra of EB-DNA in the buffer solution in the presence of increasing amount of complex **5b (A)** and **5c (B)**. The concentration of added complexes ranged from 0 to 2.0×10^{-4} M (concentration increment 1.6×10^{-5} M, indicated by different colors of the curves). The arrow indicated the changes in fluorescence at increasing amounts of the complex. The insert graph shows the plot of I_0/I vs [complex].

S5. Albumin binding studies

Albumin-binding studies of complexes **5a-c** were determined using tryptophan fluorescence quenching assays with BSA (30 μM) in buffer solution containing 15 mM trisodium citrate and 0.15 M NaCl (pH 7.0). The quenching of the emission intensity of tryptophan residues of BSA at 336 nm was monitored using increasing concentrations of complexes **5a-c**. The concentration of added complexes ranged from 0-2×10^{-5} mol mL^{-1}. The fluorescence emission spectra were recorded in the range 300-420 nm with an excitation wavelength of 280 nm. The values of the Stern-Volmer constant K_{SV} (in M^{-1}), the BSA-quenching constant k_q (in M^{-1} s^{-1}), and the BSA-binding constant K (in M^{-1}) for the interaction of the compounds with BSA have been derived from the Stern-Volmer equations (Eqs. 2 -4).

The BSA binding constants K (in M^{-1}) for the interaction of the compounds with BSA were derived from the Scatchard equation:

$$\frac{\Delta I/Io}{[Q]} = nK - K\frac{\Delta I}{Io} \qquad (5, S4\text{-}1)$$

The BSA binding constant K (in M^{-1}) can be calculated from the slope of the Scatchard plots, and the number of binding sites per albumin (n) is given by the ratio of the y-intercept to the slope.

In these experiments (UV-Vis and fluorescence measurements), we applied a dilution correction to the working standard solution to prevent dilution from affecting peak intensity.

.

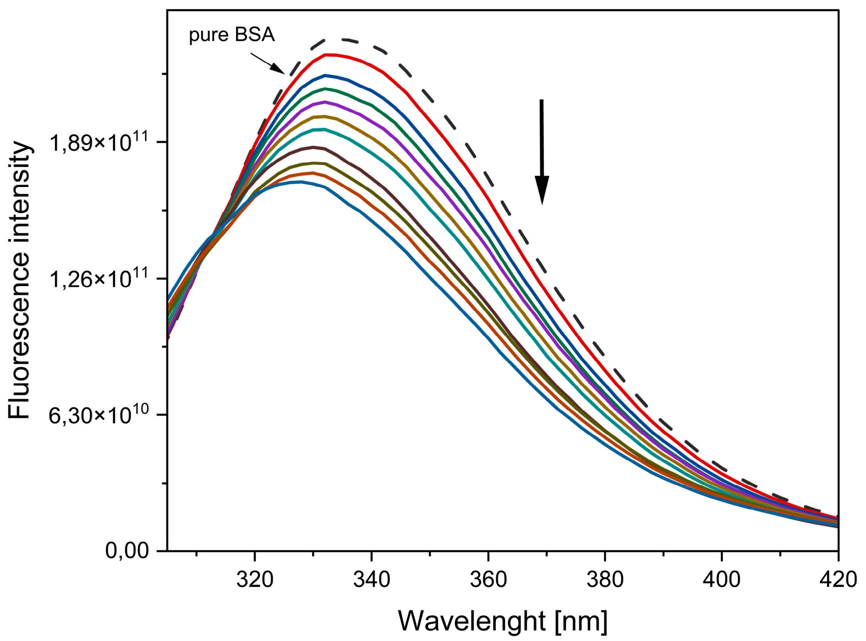

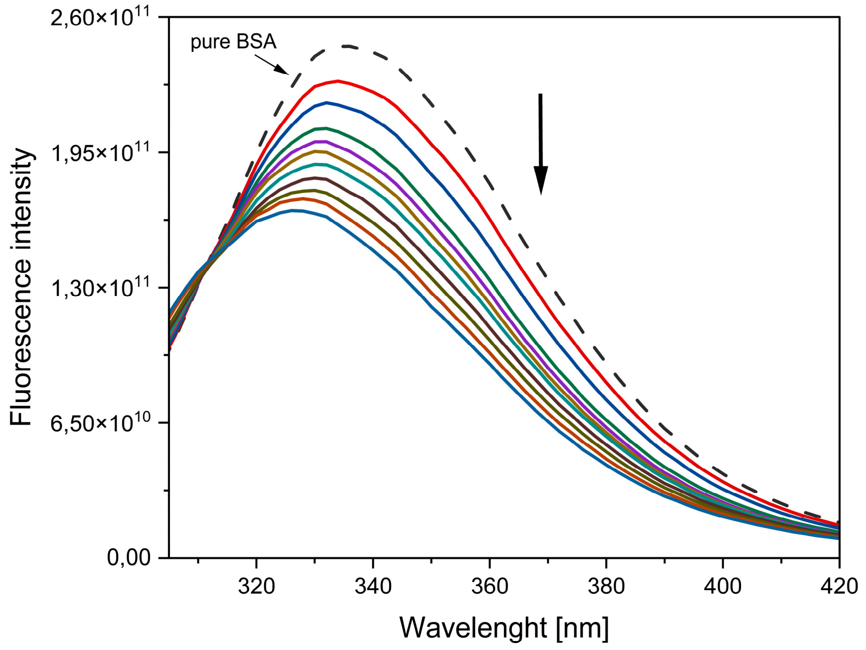

Figure S5-1: Fluorescence emission spectra of BSA in buffer solution in the presence of increasing concentrations of the complexes **5b** (up) and **5c** (down)**.** The arrow indicates the changes in fluorescence with increasing amounts of the complex. The concentration of added complexes ranged from 0 to 1.6×10^{-5} M (concentration increment 1.6×10^{-6} M, indicated by different colors of the curves). The dashed line showed the intensity in the absence of the complex.

The Stern-Volmer and Scatchard equations and graphs have been used to study the interaction of a quencher with serum albumins. The Stern-Volmer constant K_{SV} (in M^{-1}), and the quenching constant, k_q (in $M^{-1}s^{-1}$), may be derived from the Stern-Volmer equation (eq. S4-2, S4-3 and S4-4).

The BSA binding constant K (in M^{-1}) for the interaction of compounds with BSA was derived from the Scatchard equation:

$$\frac{\Delta I/Io}{[Q]} = nK - K\frac{\Delta I}{Io} \qquad \text{(S5-2)}$$

The SA-binding constant K (in M^{-1}) can be calculated from the slope in the Scatchard plots, and the number of binding sites per albumin (n) is given by the ratio of the y-intercept to the slope.

The formation of a complex in the ground state between BSA and the complexes was also confirmed by the analysis of the UV-Vis absorption spectrum. A protein titration curve to study the complex- BSA interaction was done preliminarily for the **5a** complex.

Figure S5-3 shows that the UV–Vis absorbance intensity decreased steadily with increasing complex concentration, indicating that BSA molecules associated with the complex and formed a BSA complex.

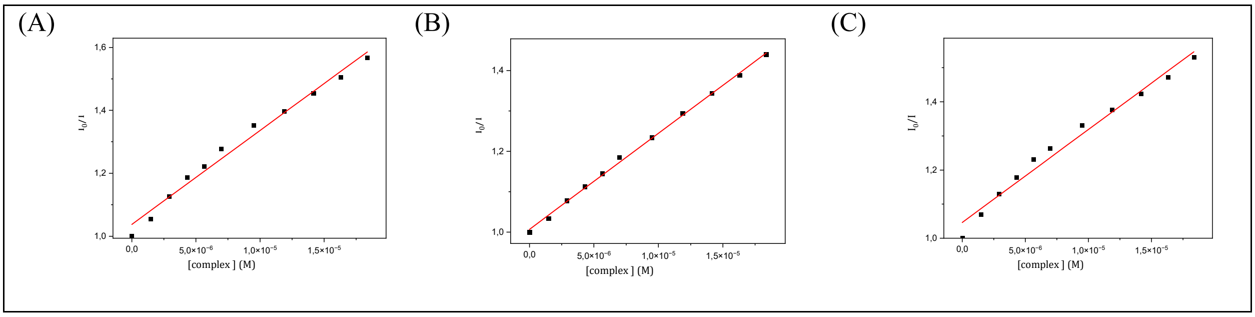

Figure S5-2: Stern-Volmer quenching plot of BSA for complex (A) **5a**, (B) **5b**, (C) **5c**.

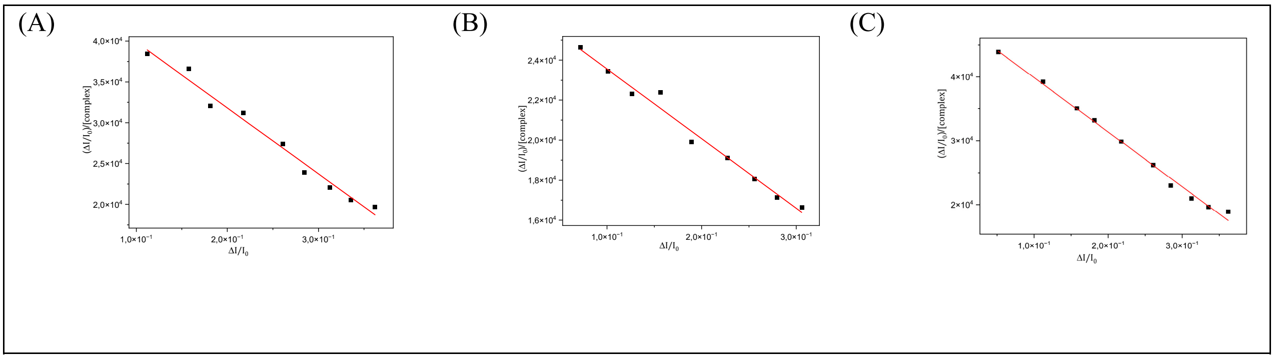

Figure S5-3: Scatchard plot of BSA for complex (A) **5a**, (B) **5b**, (C) **5c**.

S6. Resazurin Cytotoxicity Assay

The assay is based on the ability of living cells to reduce blue resazurin (7-hydroxy-3*H*-phenoxazin-3-one 10-oxide) to pink resorufin (7-hydroxy-3H-phenoxazin-3-one), which can be measured by UV-Vis spectrophotometry or fluorometry (AlamarBlue^{TM}). Living cells have active *N*-methylphenazinium methosulfate that catalyzes the redox reaction in mitochondria of NADH and blue resazurin to produce pink resorufin (Scheme S6-1). This test is used as an oxidation-reduction indicator in cell viability assays for yeast and mammalian cells. Bioreduction of the dye by viable cells reduces the oxidized form (blue) and concomitantly increases the fluorescent intermediate (pink), indicating the degree of cytotoxicity caused by the test material. The resazurin-based assay correlates well with reference viability assays such as formazan-based assays (MTT/XTT) and tritiated thymidine-based techniques.

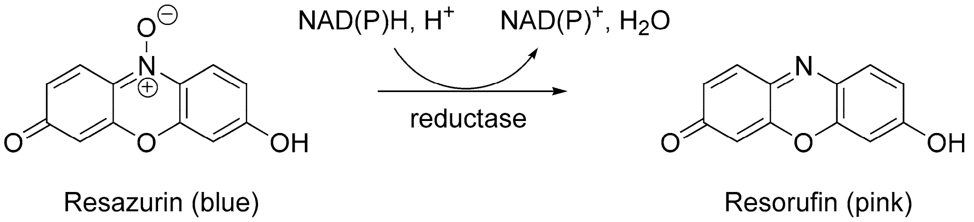

Scheme S6-1: Conversion of resazurin to resorufin in the mitochondria of living cells.

S. cerevisiae cells (CCY 21-4-64; Libáň, Czech Republic) were cultured at 28 °C/24h on Sabouraud's dextrose agar with chloramphenicol (Merck, Germany). After 24 h, *S. cerevisiae* cells were inoculated into sterile liquid YPD medium (Merck, Germany), and cultivation was again carried out at 28°C for 24 h. In the exponential growth phase, cells were centrifuged (8.000 g/3 min), washed twice with sterile physiological solution (0.9% NaCl), and pipetted at a concentration of 1×10^9 cells /mL into sterile microtubes (modified procedure from Bitacura). Sterile physiological solution and copper complexes at a concentration of 1×10^{-3} M in ratios of 0.9:0.1, 0.7:0.3, and 0.5:0.5 were added to the centrifuged biomass to a total volume of 1 mL. Thus, the final complex concentrations in the samples were 1×10^{-4} M, 3×10^{-4} M, and 5×10^{-4} M. The individual samples were cultured in an incubator at 28 °C for 3 h. After 3 h, 0.1 mL of Resazurin$_{OX}$ (resazurin-based *in vitro* toxicology assay kit, Sigma, USA) was added to each sample, and the samples were cultured for 2 h. After incubation, the biomass was separated from the supernatant by centrifugation (8.000 g /5 min). The separated solution was diluted in a 1:1 ratio with physiological solution and subjected to measurement of UV spectra by the spectrophotometric method according to the recommended protocol from the kit manufacturer (Sigma, USA) on a GENESYS 10S UV-Vis spectrophotometer (Thermo Scientific, USA) in the range of 500-800 nm, using a 1 cm long cuvette.

The cell's innate metabolic rate was measured using an established formula for percentage reduction. The percentage was calculated by the following formula:

$$Red\ (\%) = \frac{\varepsilon_{OX\_600\ nm} \times A_{570\ nm\_sample} - \varepsilon_{OX\_570\ nm} \times A_{600\ nm\_sample}}{\varepsilon_{RED\_570\ nm} \times A_{600\ nm\_C-} - \varepsilon_{RED\_600\ nm} \times A_{570\ nm\_C-}} \times 100 \qquad (7, S6\text{-}1)$$

Table S6-1: Molar extinction coefficients for reduced and oxidized Resazurin

Wavelength	Reduced Resazurin (Resorufin) ε_{RED}	Oxidized Resazurin ε_{OX}
570 nm	155,677	80,586
600 nm	14,652	117,216

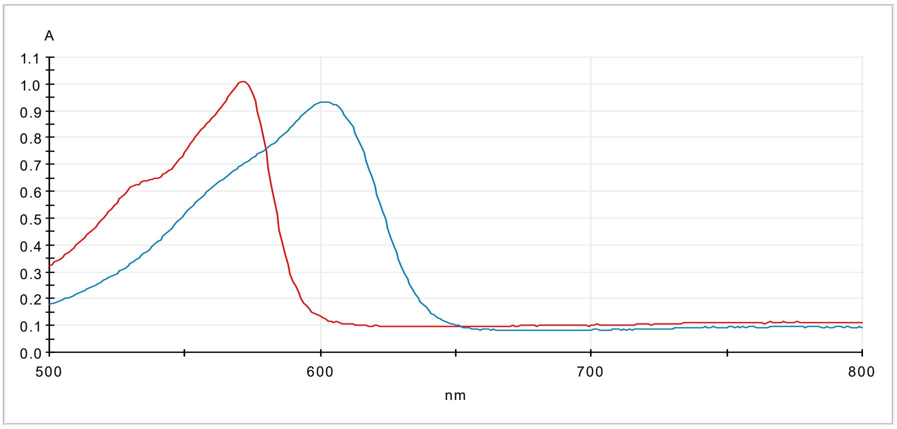

Figure S6-1: Determination of the ability of *S. cerevisiae* live cells to reduce Resazurin$_{ox}$ into Resorufin. Control samples. UV-VIS measurement range λ = 500 – 800 nm.
Blue: C- Negative Control, λ max = 601 nm (*S. cerevisiae* 1×10^9 cells/mL + 1 mL 0.9% NaCl + 0.1 mL Resazurin$_{OX}$) measured in time t_0
Red: C+ Positive Control, λ max = 572 nm (*S. cerevisiae* 1×10^9 cells/mL + 1 mL 0.9% NaCl + 0.1 mL Resazurin$_{OX}$ – which was metabolized in the mitochondria of healthy cells to Resazurin$_{RED}$ - Resorufin) measured at t = 3h

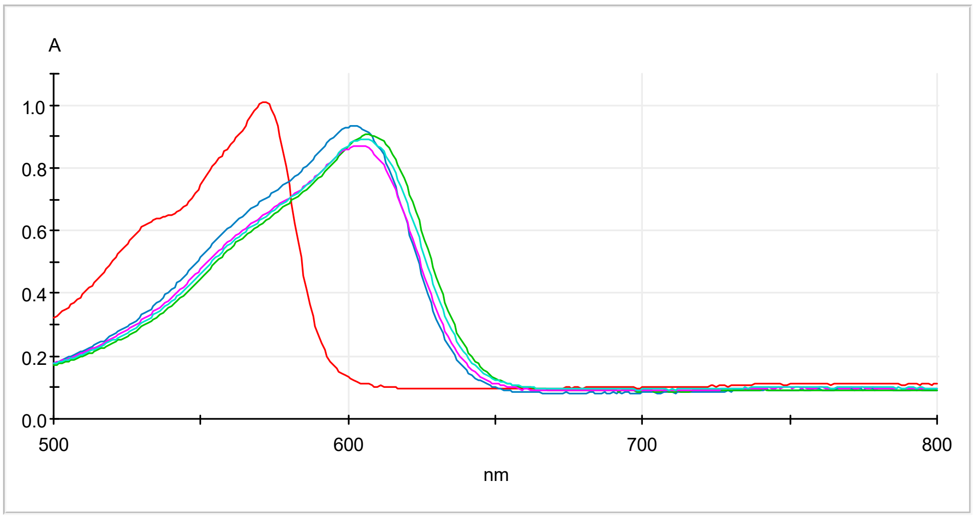

Figure S6-2: Determination of the ability of *S. cerevisiae* live cells to reduce Resazurin$_{ox}$ into Resorufin after 3 h incubation with copper complex **5a** solution.
Blue: C- Negative Control, λ max = 601 nm (*S. cerevisiae* 1*x*10^9 cells/mL + 1 mL 0.9% NaCl + 0.1 mL Resazurin$_{OX}$) measured in time t_0
Red: C+ Positive Control, λ max = 572 nm (*S. cerevisiae* 1*x*10^9 cells/mL + 1 mL 0.9% NaCl + 0.1 mL Resazurin$_{OX}$) measured in time t =3h
Magenta: **5a** 1 × 10^{-3} M (*S. cerevisiae* 1x10^9 cells /mL + 0.9 mL 0.9% NaCl + 0.1 mL **5a** + 0.1 mL Resazurin$_{OX}$) measured in time t =3h
Cyan: **5a** 3 × 10^{-3} M (*S. cerevisiae* 1x10^9 cells /mL + 0.7 mL 0.9% NaCl + 0.3 mL **5a** + 0.1 mL Resazurin$_{OX}$) measured in time t =3h
Green: **5a** 5 × 10^{-3} M (*S. cerevisiae* 1x10^9 cells /ml + 0.5 mL 0.9% NaCl + 0.5 mL **5a** + 0.1 mL Resazurin$_{OX}$) measured in time t =3h

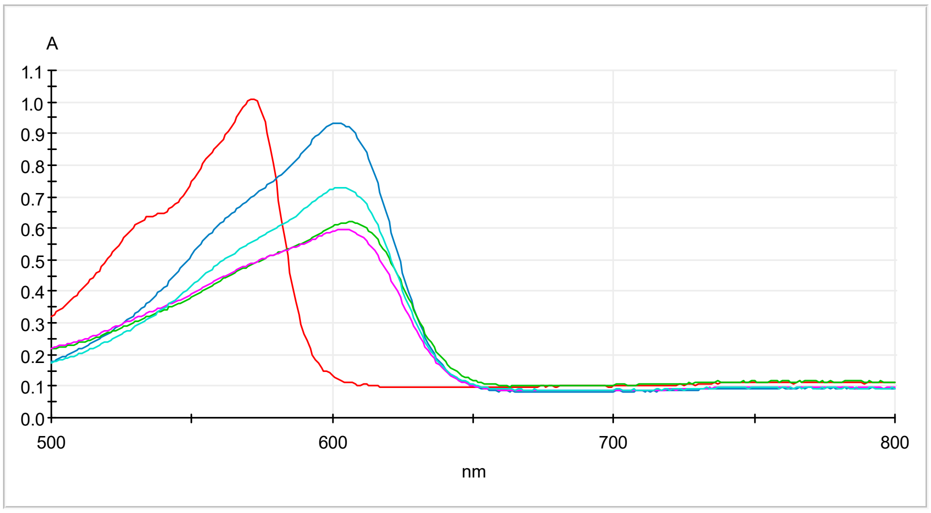

Figure S6-3: Determination of the ability of *S. cerevisiae* live cells to reduce Resazurin$_{ox}$ into Resorufin after 3 h incubation with copper complex **5b** solution.
Blue: C- Negative Control, λ max = 601 nm (*S. cerevisiae* 1*x*10^9 cells/mL + 1 mL 0.9% NaCl + 0.1 mL Resazurin$_{OX}$) measured in time t_0
Red: C+ Positive Control, λ max = 572 nm (*S. cerevisiae* 1*x*10^9 cells/mL + 1 mL 0.9% NaCl + 0.1 mL Resazurin$_{OX}$) measured in time t =3h
Cyan: **5b** 1 × 10^{-3} M (*S. cerevisiae* 1x10^9 cells /mL + 0.9 mL 0.9% NaCl + 0.1 mL **5b** + 0.1 mL Resazurin$_{OX}$) measured in time t =3h
Magenta: **5b** 3 × 10^{-3} M (*S. cerevisiae* 1x10^9 cells /mL + 0.7 mL 0.9% NaCl + 0.3 mL **5b** + 0.1 mL Resazurin$_{OX}$) measured in time t =3h
Green: **5b** 5 × 10^{-3} M (*S. cerevisiae* 1x10^9 cells /mL + 0.5 mL 0.9% NaCl + 0.5 mL **5b** + 0.1 mL Resazurin$_{OX}$) measured in time t =3h

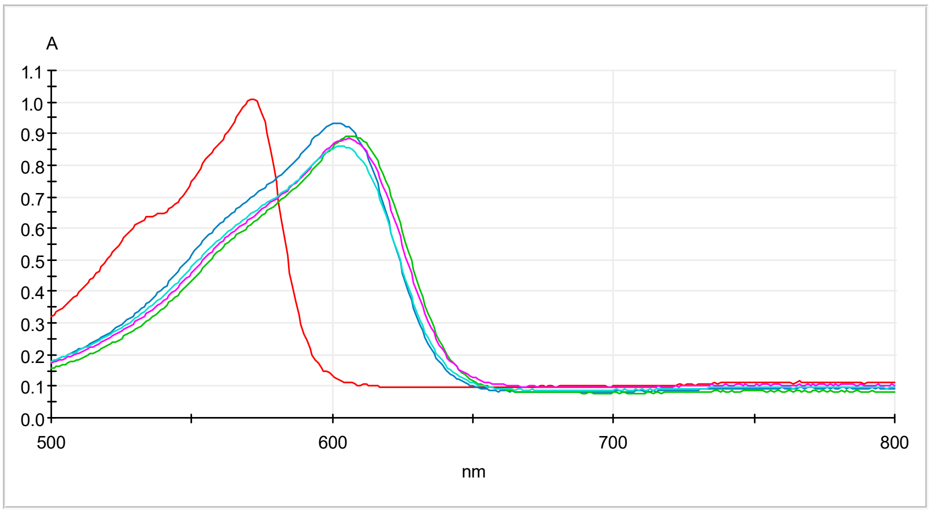

Figure S6-4: Determination of the ability of *S. cerevisiae* live cells to reduce Resazurin$_{ox}$ into Resorufin after 3h incubation with copper complex **5c** solution.
Blue: C- Negative Control, λ max = 601 nm (*S. cerevisiae* 1x10^9 cells/mL + 1 mL 0.9% NaCl + 0.1 mL Resazurin$_{OX}$) measured in time t_0
Red: C+ Positive Control, λ max = 572 nm (*S. cerevisiae* 1x10^9 cells/mL + 1 mL 0.9% NaCl + 0.1 mL Resazurin$_{OX}$) measured in time t =3h
Cyan: **5c** 1 × 10^{-3} M (*S. cerevisiae* 1x10^9 cells /mL + 0.9 mL 0.9% NaCl + 0.1 mL **5c** + 0.1 mL Resazurin$_{OX}$) measured in time t =3h
Magenta: **5c** 3 ×10^{-3} M (*S. cerevisiae* 1x10^9 cells /mL + 0.7 mL 0.9% NaCl + 0.3 mL **5c** + 0.1 mL Resazurin$_{OX}$) measured in time t =3h
Green: **5c** 5 × 10^{-3} M (*S. cerevisiae* 1x10^9 cells /mL + 0.5 mL 0.9% NaCl + 0.5 mL **5c** + 0.1 mL Resazurin$_{OX}$) measured in time t =3h

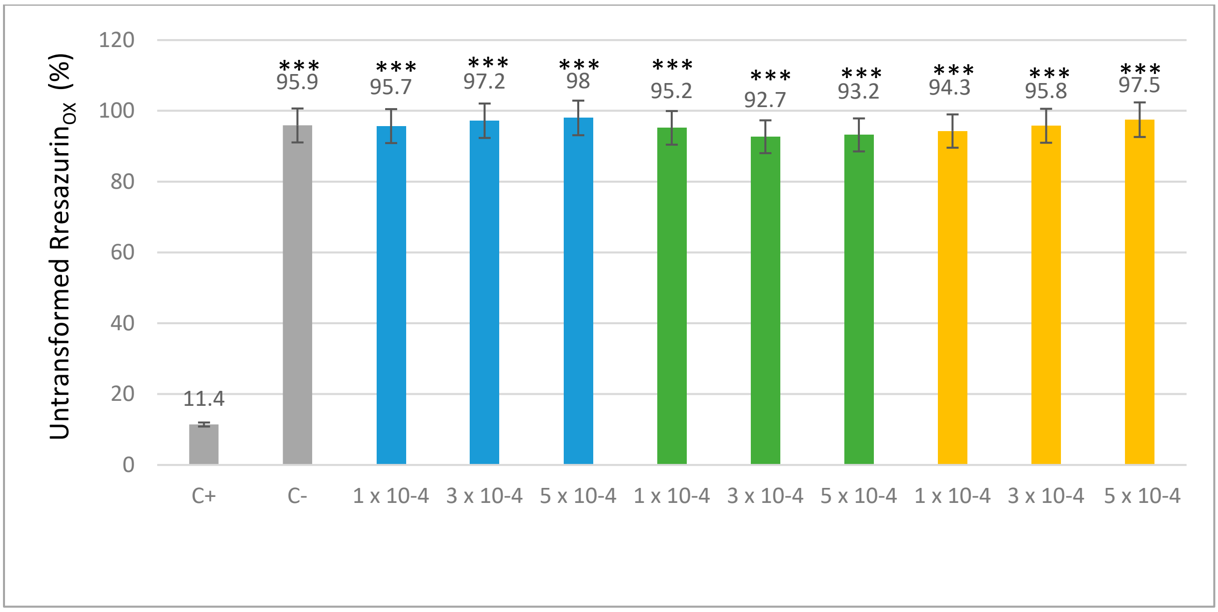

Figure S6-5: Determination of reduced metabolic activity of *S. cerevisiae* cells expressed as a percentage of the amount of untransformed Resazurin$_{OX}$ after 3 h incubation with copper complexes **5a** (blue), **5b** (green), **5c** (yellow).
Comparisons of all tested groups with the C+ control were performed by t-test. The experimental data are expressed as geometric means of double measurements ± SD. Levels of significance: ***,0.000<P<0.001; **,0.001<P<0.01; *,0.01<P<0.05.

S7. SOD mimetic activity assay

The method is based on 2-(4-iodophenyl)-3-(4-nitrophenyl)-5-phenyl-2H-tetrazolium chloride (INT), which can react with an electron from a radical (superoxide radical anion from KO_2 in our case). The reaction produces a blue-green formazan, which is detected as light absorption at 500 nm. Radical scavengers (complexes **5a-c**) compete with INT for radicals and reduce the formation of formazan and light absorption at 500 nm.

Antiradical activity measurements were performed on a Synergy HT BioTek spectrophotometer (Agilent Technologies, USA). DMSO (analytical reagent grade) was used as a solvent**,** 2-(4-Iodophenyl)-3-(4-nitrophenyl)-5-phenyl-2*H*-tetrazolium chloride **(**INT) and KO_2 were purchased from Alfa-Aesar (Thermo Fisher Scientific Inc., USA). The initial INT solution was prepared in a sodium tetraborate buffer solution with pH adjusted to 7.1 using HCl.

For each measurement, an initial solution of the sample at a specific concentration was prepared (4×10^{-3} M and 1×10^{-4} M) in DMSO, as well as initial solutions of INT (4 M) in tetraborate buffer (pH = 7.1) and a saturated solution of KO_2 in DMSO. From these, a set of diluted solutions of volume 3.5 mL was made:

A: blank solution containing only INT dye (0.5 mL of INT solution and 3 mL of DMSO), B: 3-5 control solutions containing INT dye and KO_2 (0.5 mL of INT solution, 0.5 mL of KO_2 solution and 2.5 mL of DMSO), C: 3 sample solutions containing all three compounds - the complex, INT dye and KO_2 (0.5 mL of INT solution, 0.5 mL of initial complex solution of concentration 5×10^{-3} – 3×10^{-5} M, 0.5 mL of KO_2 solution and 2 mL of DMSO), D: sample control solution containing the complex and KO_2 (0.5 mL of complex solution of concentration (5×10^{-3} – 3×10^{-5} M, 0.5 mL of KO_2 solution and 2.5 mL of DMSO).

In the sample and sample control solutions (C and D), the sample was diluted 7-fold (0.5 mL of initial sample solution in 3.5 mL of overall mixture). 200 µl of each solution was transferred into a micro-titration plate and absorbance was measured at 500 nm in parallel for all four solution types. The resulting inhibition percent was calculated based on the formula: 100 – [(absorbance of C – absorbance of D) / average absorbance of B] x 100. Each measurement was averaged across the 3 sample solutions, with standard deviation.

Table S7-1: SOD mimetic data for IC_{50} determination. The concentration of complex **5a-c** in sample solution C.

c	**5a**		**5b**		**5c**	
[M]	%	sd	%	sd	%	sd
7.14×10^{-4}	84.21	1.27	95.91	0.58	90.1	0.44
5.72×10^{-4}	74.55	0.6	90.8	0.73	81.34	0.22
4.29×10^{-4}	55.35	2.63	75.33	2.32	70	0.87
2.86×10^{-4}	41.2	0.9	69.73	0.37	55.39	1.06
1.43×10^{-4}	28.04	0.3	41.86	3.06	39.18	1.82
1.07×10^{-4}	12.45	2.32	32.7	1.26	31.83	3.1
5.72×10^{-5}	6.2	1.68	25.02	1.16	24.55	1.64
4.29×10^{-5}	nd	nd	21.28	1.54	13.28	0.52

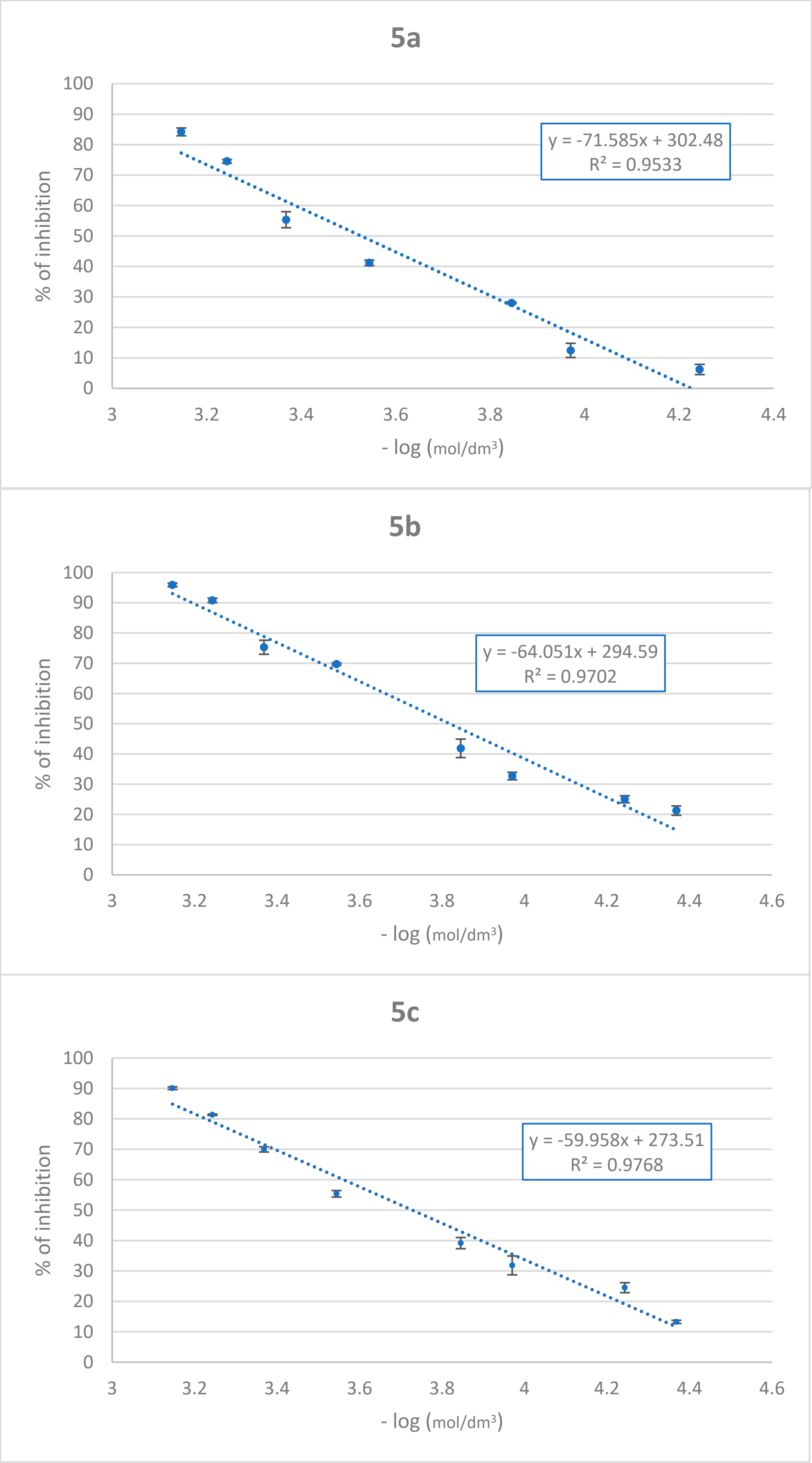

Figure S7-1: IC50 determination of SOD mimetic activity from linear trend function

S8. MTT cytotoxicity assays

The cytotoxicity of the prepared copper(II) complexes in human cell lines was evaluated using the 3-(4,5-dimethylthiazol-2-yl)-2,5-diphenyltetrazolium bromide (MTT) assay following the Mosmann protocol. Briefly, cells (A549, HepG2, and HEL) were seeded in plastic 96-well plates at a density of 1.5-2×10^4 cells per well. Cells were treated for 72 h with **5a-c** and cisplatin dissolved in DMSO and incubated at 37 °C in a 5% CO_2 atmosphere. After treatment, the samples were washed with phosphate-buffered saline (PBS), then incubated with 1 mg/mL MTT for 4 h. The MTT was then removed, and the formazan crystals were dissolved with dimethyl sulfoxide for 30 min. The photometric evaluation (at 540 nm and 690 nm) was carried out using an xMark™ Microplate Absorbance Spectrophotometer (Bio-Rad, USA).

Cell lines

Cells A549 (ATCC® CCL-185), HepG2 (ATCC® HB-8065), and HEL299 (ATCC® CCL-137) were obtained from Lambda Life (Bratislava, Slovakia). A549 cells were kept in Dulbecco's Modified Eagle's Medium (DMEM) supplemented with 10% fetal bovine serum (FBS) and 1% antibiotics (penicillin and streptomycin). HepG2 cells were kept in Williams medium supplemented with 10% fetal bovine serum (FBS) and 1% antibiotics (penicillin and streptomycin). HEL299 cells were kept in Minimum Essential Medium (MEM) supplemented with 10% fetal bovine serum (FBS), 1% antibiotics (penicillin and streptomycin), and 1% non-essential amino acids (NEAA). Cells were cultured in a humidified incubator at 37 °C with 5% CO_2. Once the cells reached approximately 80% confluence, they were harvested and subcultured after trypsin treatment.

Table S8-1: MTT test IC_{50} values in μM ranges and selectivity indexes where SI = IC_{50} HEL/IC_{50} cancer cells

Compound	IC_{50} – A549	IC_{50} – HepG2	IC_{50} - HEL	SI (HEL/ HepG2)	SI (HEL/A549)
**5a**	123.4 ± 16.5	44.7 ± 6.4	136.5 ± 16.8	**3.05**	1.10
**5b**	85.8 ± 1.1	33.0 ± 5.3	81.7 ± 3.3	**2.48**	0.95
**5c**	91.4 ± 9.2	42.4 ± 4.7	88.4 ± 16.3	**2.08**	0.97
cisplatin	26.2 ± 4.1	16.0 ± 3.4	68.4 ± 0.3	**4.28**	2.61
